# Design, Synthesis, and Structure–Activity Relationship Studies of Spirotryprostatin A-Inspired Spirooxindole-Diketopiperazine Derivatives with Antifungal Activity

**DOI:** 10.3390/molecules31213846

**Published:** 2026-10-25

**Authors:** Murong Zhan, Yangmin Ma, Gege Dang, Zhichao Wang, Menghua Shang, Qian Jiao

**Affiliations:** Key Laboratory of Chemical Additives for China National Light Industry, College of Chemistry and Chemical Engineering, Shaanxi University of Science and Technology, Xi’an 710021, China; bs240811027@sust.edu.cn (M.Z.); 220811008@sust.edu.cn (G.D.); 230811053@sust.edu.cn (M.S.); 240811015@sust.edu.cn (Q.J.)

**Keywords:** spirotryprostatin A derivatives, spirooxindole-diketopiperazines, structure–activity relationship (SAR), antifungal activity, molecular docking

## Abstract

To address the escalating challenge of fungal resistance in agriculture and explore structurally diverse antifungal scaffolds, a series of novel spirotryprostatin A-inspired spirooxindole-diketopiperazine derivatives was designed and synthesized. Seven spirooxindole intermediates and fourteen target derivatives were successfully obtained and systematically evaluated for their antifungal activities against twelve phytopathogenic fungi. Structure–activity relationship (SAR) analyses indicated that incorporation of the diketopiperazine framework contributed to improved antifungal activity, while the electronic properties and substitution patterns of aromatic substituents influenced the biological profiles. Generally, compounds with electron-withdrawing groups (EWGs) exhibited relatively improved antifungal activity compared with those bearing electron-donating groups (EDGs), with *ortho*-trifluoromethyl substitution showing a favorable effect. Among the synthesized derivatives, compounds **4e** and **4e′** exhibited promising broad-spectrum antifungal activity, with MIC values of 4 μg/mL observed against some tested phytopathogenic fungi. Isothermal microcalorimetry (IMC) experiments further supported the antifungal potential of **4e**. Additionally, molecular docking studies suggested that the selected active derivative could interact with succinate dehydrogenase (SDH) and myosin through hydrogen-bonding and hydrophobic interactions, providing preliminary insights into possible binding modes. Overall, this study expands the structural diversity of spirotryprostatin A-inspired spirooxindole-diketopiperazines and provides valuable information for the discovery and optimization of novel antifungal compounds.

## 1. Introduction

Ensuring global food security is the core of modern agricultural development [1]. According to statistics from the Food and Agriculture Organization (FAO) of the United Nations, diseases caused by plant pathogenic fungi result in a 10% to 15% loss of global crop yields annually [2]. For a long time, agricultural disease control has primarily relied on traditional chemical pesticides [3,4]. However, their prolonged and unreasonable application has not only exacerbated ecological and environmental pollution but also triggered a sharp increase in the multidrug resistance of pathogenic fungi, posing severe challenges to the efficacy of traditional fungicides [5]. Major fungicide classes used in crop protection include demethylation inhibitors (DMIs), quinone outside inhibitors (QoIs), succinate dehydrogenase inhibitors (SDHIs), and methyl benzimidazole carbamates (MBCs), among others. The emergence of resistance to several widely used fungicide classes has highlighted the need for novel antifungal compounds with diverse chemical scaffolds.

**Figure 1:**
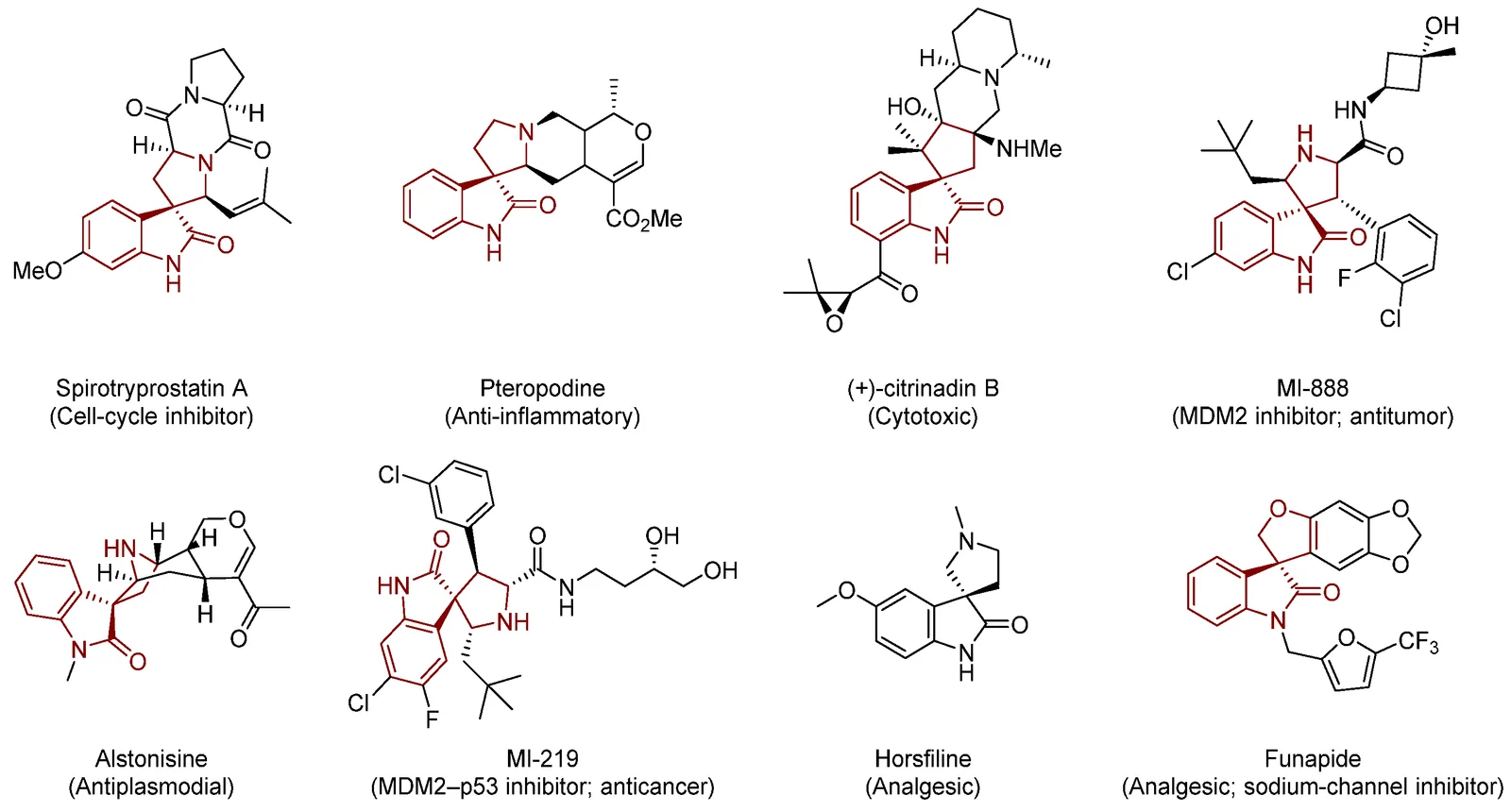
Examples of natural spirooxindole alkaloids and their structurally related derivatives [6,7,8,9,10,11,12,13].

Structural modification and derivatization based on natural products are effective approaches for creating novel pesticide compounds. The spirooxindole framework is a privileged scaffold widely found in structurally diverse natural alkaloids, including spirotryprostatin A, pteropodine, (+)-citrinadin B, alstonisine, and horsfiline, as well as synthetic bioactive molecules such as MI-219, MI-888, and funapide (Figure 1). Spirooxindole-containing compounds have been reported to exhibit diverse biological activities, including cell-cycle inhibitory, anti-inflammatory, cytotoxic, antitumor, antiplasmodial, and analgesic activities [6,7,8,9,10,11,12,13]. Meanwhile, the diketopiperazine unit, with its rigid heterocyclic skeleton and favorable metabolic stability, can significantly enhance the binding affinity between ligands and target proteins through multiple hydrogen-bonding interactions [14,15]. In recent years, the combination of spirooxindole and diketopiperazine pharmacophores through molecular hybridization strategies has emerged as an effective approach for constructing structurally diverse bioactive molecules [16,17,18]. Spirotryprostatin A, a representative spirooxindole alkaloid containing a diketopiperazine framework, has attracted considerable attention because of its unique structural characteristics and biological potential. Inspired by this natural product scaffold, spirooxindole-diketopiperazine derivatives have been explored as promising frameworks for the discovery of novel bioactive compounds [19,20].

However, the biological activity of spirooxindole-diketopiperazine derivatives can be influenced by structural variations, particularly the electronic properties, steric effects, and substitution patterns of aromatic substituents [21,22,23]. These factors can regulate molecular polarity, steric environment, and conformational characteristics, thereby influencing their biological performance [24,25]. Therefore, systematic structural diversification and structure–activity relationship (SAR) investigations are important for exploring the relationship between structural features and biological activity within this scaffold [26,27]. The introduction of substituents with different electronic properties provides an effective strategy for exploring SAR trends. Electron-donating groups (EDGs) and electron-withdrawing groups (EWGs) can alter electron distribution, polarity, and molecular recognition, while different substitution patterns may further influence steric interactions and molecular conformation [28,29,30]. To investigate the influence of electronic effects, methyl (–CH_3_) and trifluoromethyl (–CF_3_) groups were selected as representative EDG and EWG substituents, respectively. These two groups possess distinct electronic properties while exhibiting relatively moderate steric differences, providing suitable models for investigating how electronic properties influence antifungal activity [31,32,33,34]. In addition, the trifluoromethyl group is widely applied in medicinal and agrochemical chemistry because of its ability to regulate lipophilicity, metabolic stability, and physicochemical properties.

In this study, a series of novel Spirotryprostatin A-inspired spirooxindole-diketopiperazine derivatives were designed and synthesized through systematic structural diversification of aromatic substituents. Seven spirooxindole intermediates and fourteen target derivatives were successfully obtained. By introducing electron-donating and electron-withdrawing groups at different positions of the aromatic ring, the influence of electronic properties, substitution patterns, and stereochemical configurations on antifungal activity was investigated. The antifungal activities of these derivatives against twelve major phytopathogenic fungi were evaluated by determining minimum inhibitory concentration (MIC) values, and comprehensive SAR analyses were performed to elucidate the relationship between molecular structure and biological activity. Furthermore, isothermal microcalorimetry (IMC) was employed to investigate the metabolic responses of representative fungal strains under compound treatment, while molecular docking studies were conducted to provide preliminary insights into the possible molecular interactions of highly active derivatives with potential fungal targets. This study provides a structural optimization strategy for Spirotryprostatin A-inspired scaffolds and contributes to the discovery of novel antifungal compounds.

## 2. Results and Discussion

### 2.1. Synthesis of Spirotryprostatin A-Inspired Spirooxindole-Diketopiperazine Derivatives

**Scheme 1:**
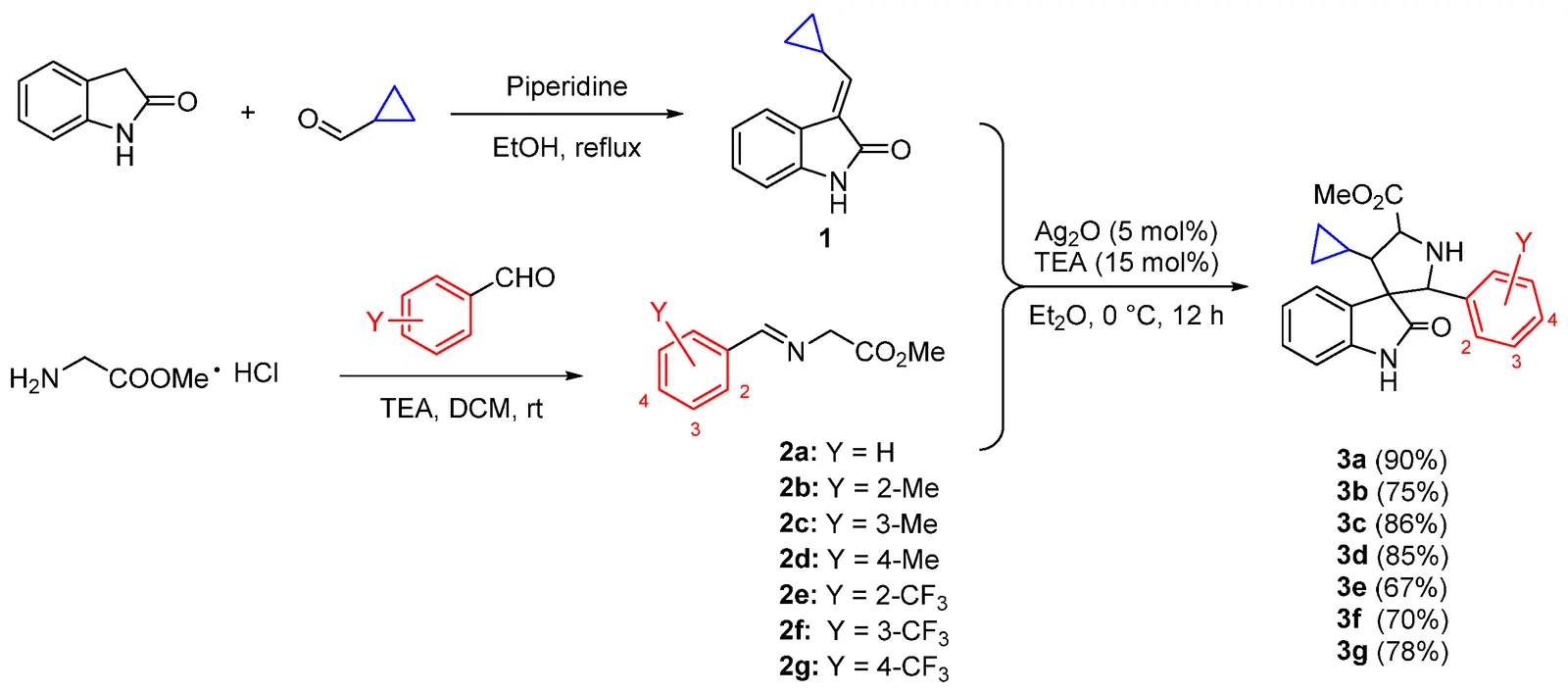
Preparation of spirooxindole intermediates **3a**–**3g**.

The synthetic strategy was designed based on structural diversification of aromatic substituents on the spirotryprostatin A-inspired spirooxindole-diketopiperazine scaffold to investigate the effects of electronic properties and substitution patterns on antifungal activity. The spirooxindole intermediates were initially synthesized via a catalytic [3 + 2] cycloaddition reaction between oxindole 1 and various glycine ester imines (**2a**–**2g**) in the presence of Ag_2_O and Et_3_N (Scheme 1). This transformation provided the key spirooxindole intermediates **3a**–**3g**, which served as versatile precursors for the subsequent construction of the target spirooxindole-diketopiperazine derivatives.

**Scheme 2:**
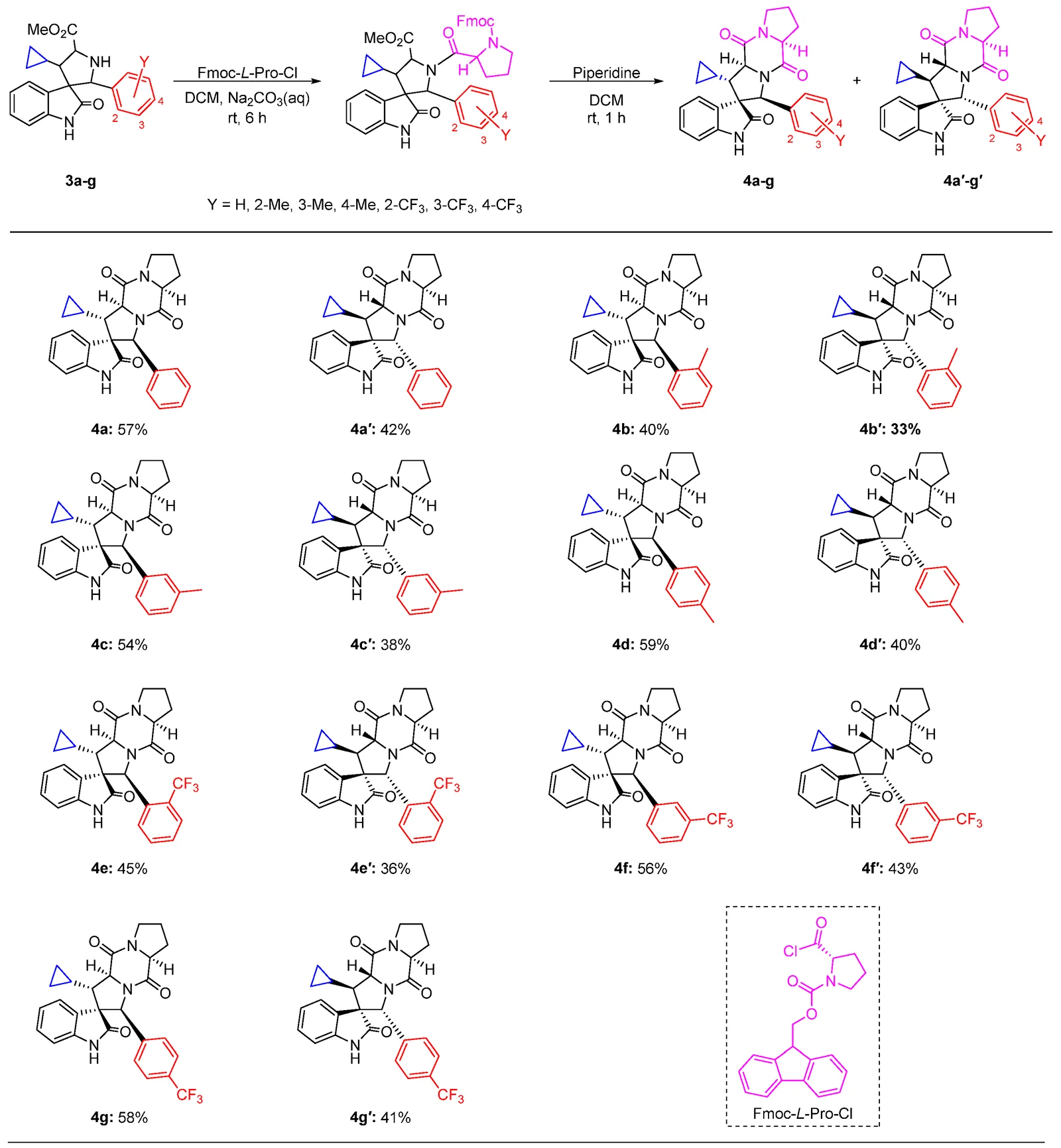
Preparation of spiroindolodione piperazine compounds **4a**–**4g** and **4a′**–**4g′** ^a,b^. ^a^ Reaction conditions: **3a**–**3g** (0.1 mmol) and Fmoc-L-Pro-Cl (0.1 mmol) in DCM (5.0 mL) and Na_2_CO_3_ (5 mL) at room temperature; piperidine (0.12 mmol) at room temperature. ^b^ Isolated yields.

**Figure 2:**
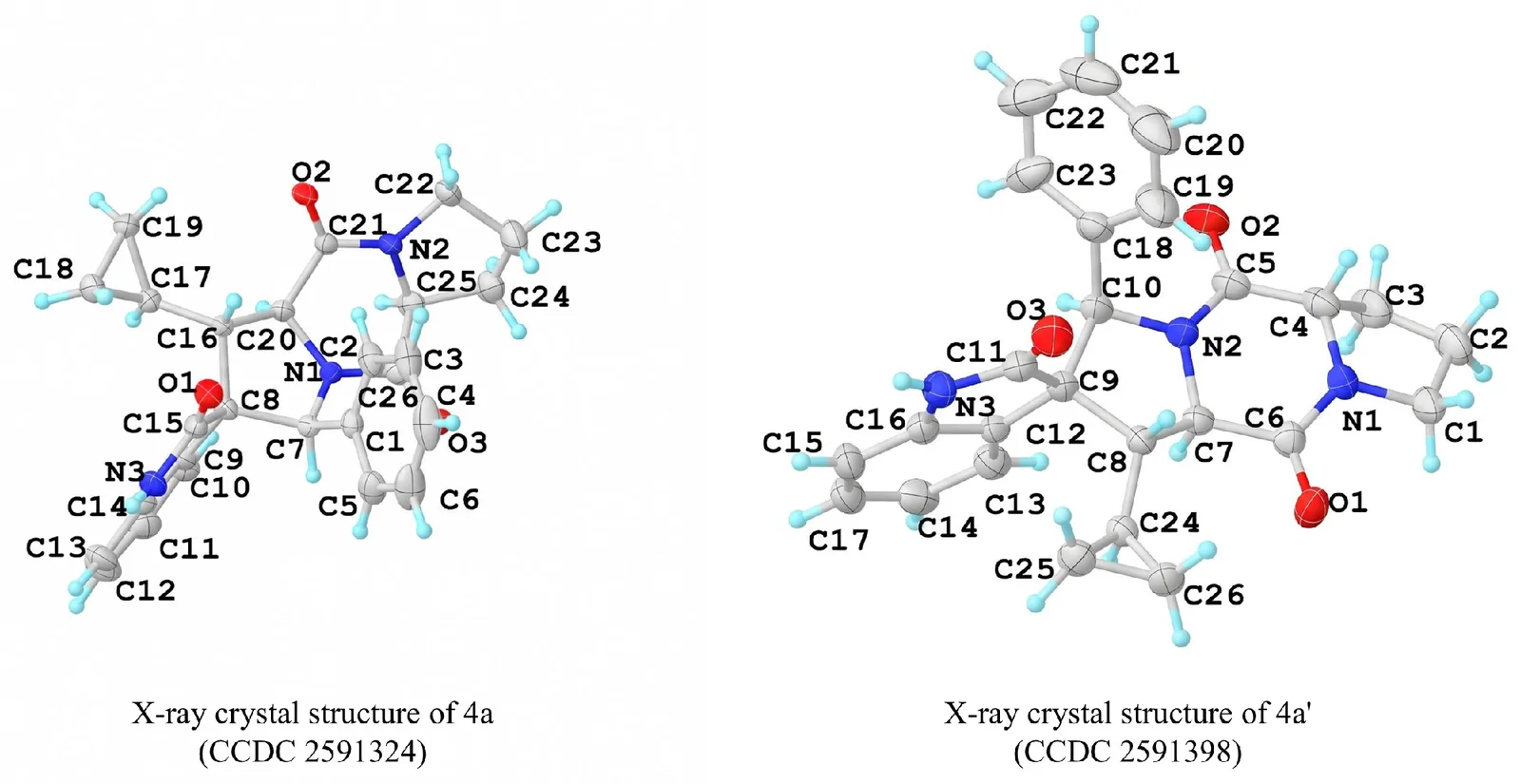
The X-ray crystal structure of **4a** and **4a′**.

During the synthesis of the target spirooxindole-diketopiperazine derivative 4, Fmoc-L-Pro-Cl was employed as the acylating reagent to construct the dipeptide intermediates via a Schotten–Baumann reaction. This step introduced the proline-derived chiral unit into the molecular framework. Subsequently, removal of the Fmoc protecting group was achieved under basic conditions using piperidine, followed by spontaneous intramolecular cyclization to generate the diketopiperazine ring system. The resulting products were purified by silica gel column chromatography to afford the target compounds **4** and **4′** (Scheme 2). The absolute configurations of representative derivatives were confirmed by single-crystal X-ray diffraction analysis (Figure 2). To systematically investigate the effects of electronic properties and substitution patterns on the molecular diversity of the target compounds, electron-withdrawing and electron-donating groups were strategically introduced at the *ortho*-, *meta*-, and *para*-positions of the aromatic ring. The target products **4** and **4′** were generally obtained in moderate to excellent yields (77–99%). However, compounds **4b**, **4b′**, **4e**, and **4e′** were obtained in relatively lower yields compared with other derivatives, which may be associated with increased steric hindrance during the intramolecular cyclization step.

### 2.2. Preliminary Antifungal Evaluation of Spirotryprostatin A-Inspired Spirooxindole-Diketopiperazine Derivatives

In this study, seven spirooxindole intermediates (**3a**–**3g**) and fourteen spirooxindole-diketopiperazine derivatives (**4a**–**4g** and **4a′**–**4g′**) were initially evaluated for their in vitro antifungal activities against twelve phytopathogenic fungi using the agar diffusion method. Ketoconazole (2 mg/mL) was used as the positive control, while DMSO served as the negative control. The antifungal profiles of the tested compounds were visualized by heatmap analysis (Supplementary Materials, Figure S43), in which the color intensity corresponded to the diameter of inhibition zones (mm), allowing an intuitive comparison of inhibitory effects among different compounds and fungal strains.

SAR analysis revealed that the spirooxindole-diketopiperazine derivatives (**4a**–**4g** and **4a′**–**4g′**) generally exhibited enhanced antifungal activity compared with the corresponding spirooxindole intermediates (**3a**–**3g**). The introduction of the diketopiperazine framework significantly improved the inhibitory effects against several tested fungal strains, indicating the importance of this structural motif for antifungal activity. Among the tested phytopathogens, the synthesized compounds displayed particularly pronounced inhibitory effects against *Valsa mali* (*V. m.*) and *Alternaria brassicae* (*A. b.*).

### 2.3. Minimum Inhibitory Concentration (MIC) Evaluation and Structure–Activity Relationship Studies of Spirooxindole-Diketopiperazine Derivatives

The minimum inhibitory concentration (MIC) is a critical parameter for evaluating the antimicrobial efficacy of bioactive compounds and serves as an important indicator in the screening and quantitative assessment of antifungal agents. To systematically evaluate the inhibitory effects of the 21 synthesized compounds against 12 plant pathogenic fungi, the MIC values of all derivatives were determined for each fungal strain, and their antifungal activities were comprehensively analyzed (Table 1).

The MIC results demonstrated that the antifungal activities of the synthesized compounds varied significantly depending on their molecular structures, indicating that structural modifications of the spirooxindole-diketopiperazine scaffold had a substantial influence on antifungal potency. Meanwhile, different fungal strains exhibited distinct sensitivity profiles toward these compounds, suggesting the presence of species-specific variations in susceptibility.

**Figure 3:**
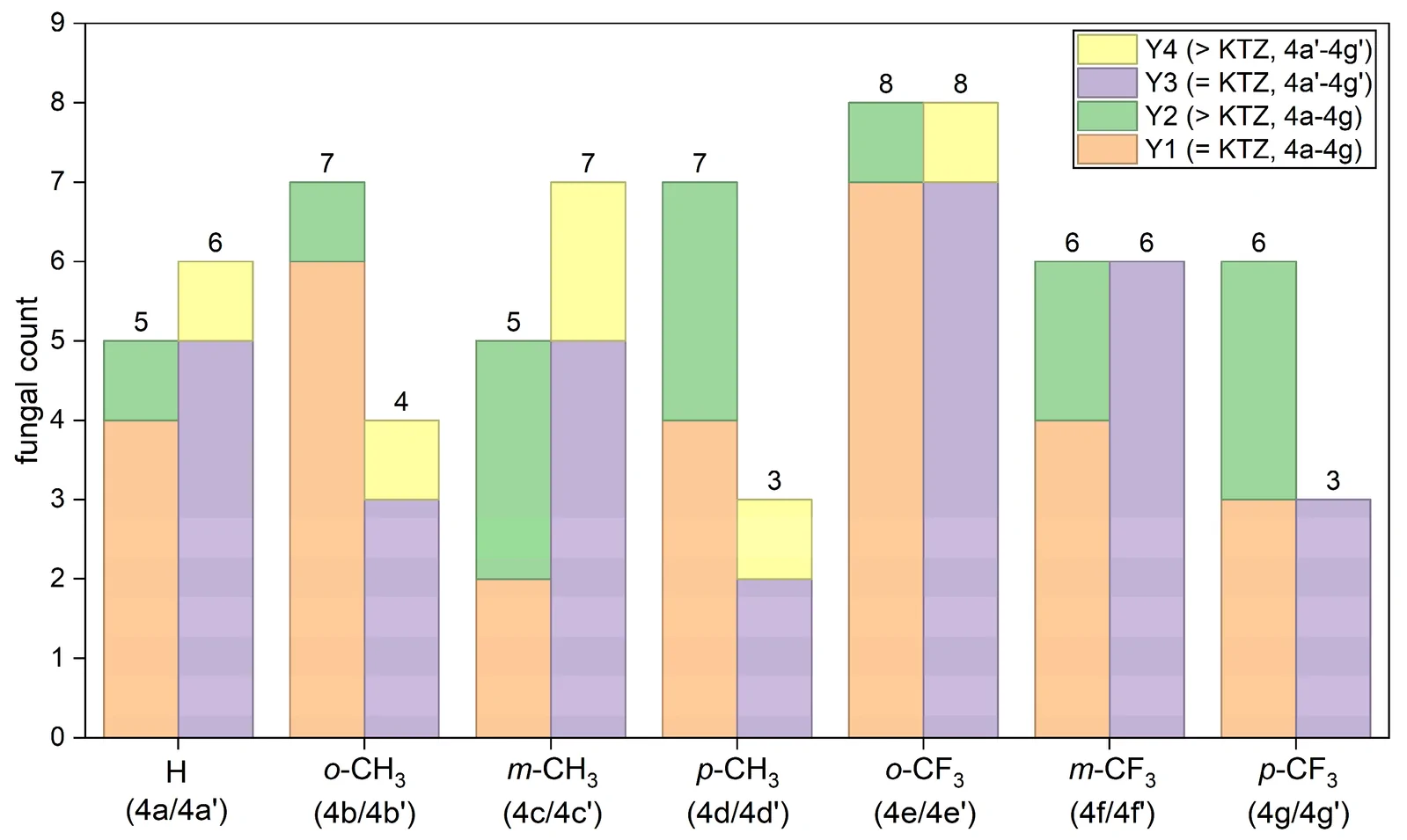
The number of fungal strains in different substituent series that achieved or surpassed the efficacy of ketoconazole.

To further evaluate and quantify the broad-spectrum antifungal potency of the final products in each substituent series (**4a**–**4g**, **4a′**–**4g′**), this study used the broad-spectrum antifungal agent ketoconazole (KTZ) as a positive control, and for the 12 plant pathogenic fungi, counted the frequency of strains for which the compounds in each substituent series (e.g., **4b** and its diastereomer **4b′**) exhibited antifungal activity equivalent to (=KTZ) or superior to (>KTZ) that of ketoconazole (Figure 3).

The in vitro antifungal activity assay demonstrated that, compared with the unsubstituted phenyl ring compounds **4a**/**4a′** of the spirooxindole diketopiperazine final products, the compounds **4b**–**4d** and **4b′**–**4d′** bearing an electron-donating group (-CH_3_) and the compounds **4e**–**4g** and **4e′**–**4g′** bearing a strong electron-withdrawing group (-CF_3_) all exhibited varying degrees of improvement in overall antifungal activity. For the two series with methyl and trifluoromethyl substituents, a horizontal comparison based on the specific MIC values in Table 1 revealed that the influence of the substituent position (-*ortho*, -*meta*, -*para*) on activity exhibited different trends under different electronic effects.

In the unsubstituted (-H) series on the phenyl ring, compound **4a** exhibited MIC values against *C.l.* (16 μg/mL), *P.o.* (16 μg/mL), *P.p.* (8 μg/mL), and *F.o.v.* (32 μg/mL) that were equal to those of the positive control ketoconazole, and its MIC value against *V.m.* (4 μg/mL) was lower than that of ketoconazole. The diastereomer **4a′** showed the same effect as **4a** against *C.l.* and *P.p.*, but exhibited better inhibitory effects than **4a** against *M.m.* (32 μg/mL), *F.g.* (16 μg/mL), and *P.o.* (8 μg/mL).

In the methyl (-CH_3_) series, the ranking of the superiority frequencies by substituent position was *meta*-substitution (m-CH_3_) (**4c**/**4c′**, 12 strains) > *ortho*-substitution (o-CH_3_) (**4b**/**4b′**, 11 strains) > *para*-substitution (p-CH_3_) (**4d**/**4d′**, 10 strains). Among them, the *meta*-substituted compound **4c** exhibited the broadest and most excellent antifungal activity. Specifically, against *F.g.* (16 μg/mL) and *V.m.* (8 μg/mL), **4c** displayed antifungal activity comparable to that of the positive control ketoconazole; against *A.b.* (4 μg/mL), *A.s.* (4 μg/mL), and *C.g.* (16 μg/mL), **4c** exhibited superior antifungal activity to ketoconazole. Interestingly, the diastereomer **4c′** showed excellent antifungal activity different from that of **4c** against *M.m.* (16 μg/mL), *A.a.* (8 μg/mL), and *F.o.v.* (32 μg/mL). The *ortho*-substituted compounds performed excellently on specific strains: **4b** exhibited antifungal activity comparable to ketoconazole against six strains including *M.m.* (32 μg/mL), *F.o.n.* (8 μg/mL), and *A.a.* (8 μg/mL), and showed stronger antifungal activity than ketoconazole against *A.s.* (8 μg/mL). Furthermore, the diastereomer **4b′** also exhibited antifungal activity different from that of **4b** against *P.p.* (8 μg/mL), and its antifungal activity against *A.b.* (4 μg/mL) was superior to that of ketoconazole. Although the para-substituted compounds **4d**/**4d′** had slightly lower superiority frequencies than the *meta*- and *ortho*-substituted ones, they still exhibited excellent inhibitory effects against eight strains. In particular, **4d** showed particularly outstanding activity against *F.g.* (8 μg/mL), *C.l.* (8 μg/mL), and *A.s.* (8 μg/mL). Based on the above broad-spectrum or specific performance, compounds **4c** and **4d** demonstrated potential value for development as highly effective narrow-spectrum fungicides.

In the trifluoromethyl (-CF_3_) series, the pattern of superiority frequencies by substituent position was *ortho* (**4e**/**4e′**, 16 strains) > *meta* (**4f**/**4f′**, 12 strains) > *para* (**4g**/**4g′**, 9 strains). The *ortho*-substituted series showed a favorable overall antifungal profile and promising broad-spectrum activity among all series: **4e** showed excellent inhibitory effects on various strains such as *M.m.* (32 μg/mL), *C.l.* (16 μg/mL), *A.a.* (8 μg/mL), *P.o.* (8 μg/mL), *P.p.* (4 μg/mL), *A.s.* (16 μg/mL), *V.m.* (8 μg/mL), and *F.o.v.* (32 μg/mL); its diastereomer **4e′** exhibited stronger inhibitory effects than **4e** against *F.g.* (16 μg/mL), *A.b.* (8 μg/mL), and *V.m.* (4 μg/mL). The *meta*-substituted compound **4f** exhibited excellent inhibitory effects against *F.g.* (16 μg/mL), *F.o.n.* (8 μg/mL), *C.l.* (8 μg/mL), *A.b.* (8 μg/mL), *P.p.* (8 μg/mL), and *C.g.* (16 μg/mL) and other strains. Its diastereomer **4f′** showed better inhibitory effects than **4f** against *M.m.* (32 μg/mL) and *A.s.* (16 μg/mL). The para-substituted compounds **4g**/**4g′** exhibited excellent inhibitory effects against eight strains. Among them, **4g** demonstrated exceptionally outstanding antifungal effects against three strains, namely *P.o.* (8 μg/mL), *A.s.* (8 μg/mL), and *V.m.* (4 μg/mL), with MIC values lower than that of ketoconazole, while the diastereomer **4g′** exhibited better inhibitory activity than **4g** against *F.o.n.* (8 μg/mL) and *A.b.* (8 μg/mL).

Structure–activity relationship (SAR) analysis indicates that the antifungal activity of this series of compounds is primarily regulated by the substituent’s electronic properties and substitution position, with molecular stereoconfiguration exerting a relatively minor impact. Overall, the electronic effect plays a significant role [35]: electron-withdrawing groups (e.g., -CF_3_), exhibit significantly superior antifungal efficacy compared to electron-donating groups (e.g., -CH_3_) by rendering the phenyl ring electron-deficient, thereby substantially enhancing both overall activity and broad-spectrum efficacy [36]. Regarding positional effects, different trends emerge: for electron-withdrawing groups, the activity follows the order of *ortho* > *meta* > *para*; conversely, for electron-donating groups, the order is *meta* > *ortho* > *para*. In contrast, the stereoisomeric effect acts only as a minor secondary factor that does not fundamentally alter the aforementioned overall SAR trends. In terms of future molecular design strategies based on these findings: for electron-donating *ortho*/*para*-directing groups (such as methyl), *meta*-modification should be prioritized to minimize electronic interference, whereas, for electron-withdrawing *meta*-directing groups (such as trifluoromethyl) [36,37,38], *ortho*- or *meta*-modification should be preferentially considered, aiming to utilize the strong electron-withdrawing inductive effect to strengthen the binding affinity with the receptor while avoiding the steric geometric limitations imposed by *para*-modification [39].

### 2.4. Heat Flow Curve Analysis of Spirooxindole-Diketopiperazine Derivatives

**Figure 4:**
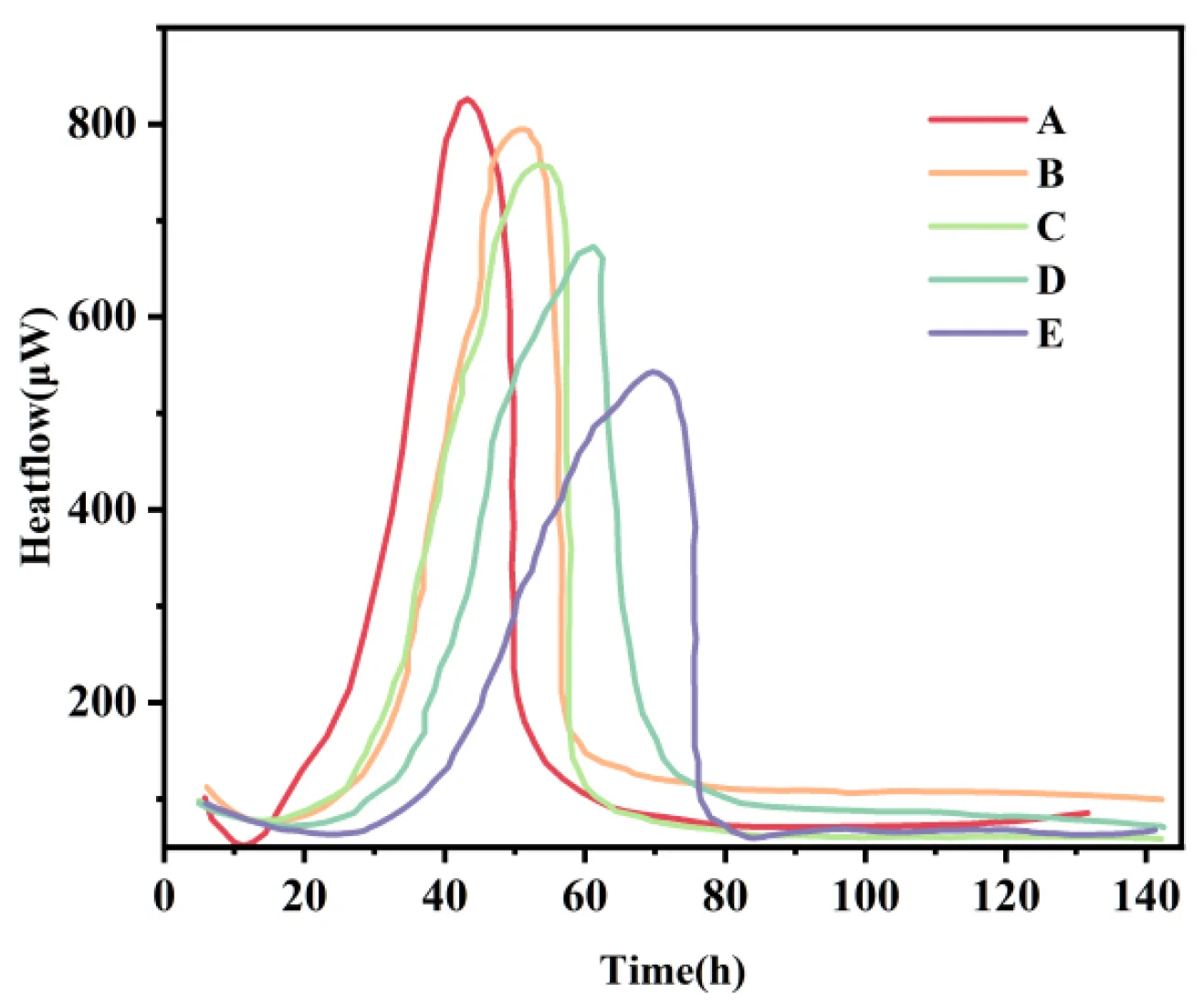
Isothermal microcalorimetry heat flow–time curves of *Valsa mali* in the presence of compound **4e** at various concentrations: (A) 0 μg/mL, (B) 1 μg/mL, (C) 2 μg/mL, (D) 4 μg/mL, and (E) 8 μg/mL.

Isothermal microcalorimetry possesses extremely high sensitivity to the heat released by metabolically active cells, enabling real-time, non-destructive recording of energy changes during microbial metabolic processes. In this study, compound **4e** (which exhibited antifungal activity comparable to that of the positive control ketoconazole and demonstrated broad-spectrum antifungal activity) was selected, and the *Valsa mali*, the most sensitive strain to this series of compounds, was used as the test fungus for heat flow curve analysis by isothermal microcalorimetry [40]. Under isothermal conditions, this experiment monitored in real time the energy and heat changes accompanying the metabolic process of *Valsa mali* under the action of different concentrations (0, 1, 2, 4, 8 μg/mL) of the spirooxindole diketopiperazine compound **4e**, and obtained the corresponding metabolic power–time curves (Figure 4) and related biometabolic kinetic parameters (Table 2).

As illustrated in Figure 4, the heat flow curves of *Valsa mali* exhibited a distinct concentration-dependent response upon treatment with compound **4e**, characterized by a progressive suppression of the exothermic peak intensity and a pronounced rightward shift in the metabolic profiles. Quantitative kinetic analysis (Table 2) revealed that the maximum metabolic power (*P_m_*) of the control group was 835.20 ± 12.8 μW, with a peak time (*t_m_*) of 43.92 ± 1.5 h. Upon increasing the concentration of **4e** to 8 μg/mL, the *P_m_* value step-wisely declined to 549.53 ± 11.5 μW, whereas the *t_m_* was substantially prolonged to 70.48 ± 2.8 h. These findings demonstrate that compound **4e** effectively suppresses the ceiling of energy metabolism during the late log phase of the fungus and drastically extends its lag phase, manifesting a significant kinetic delay in fungal proliferation.

Intriguingly, the specific growth rate constant (*μ*) of *Valsa mali* did not experience a catastrophic collapse across the investigated concentration range, remaining relatively constant between 0.017 and 0.022 h^−1^. This phenomenon indicates that the primary mode of action of compound **4e** is fungistatic rather than strictly fungicidal. The compound impedes the initial physiological preparation and confines the maximum metabolic burst scale, yet the intrinsic reproductive velocity of the surviving fungal cells remains uncompromised once they enter the exponential growth phase. The determination coefficients (*R*^2^) for all kinetic fits ranged from 0.81 to 0.87, underscoring the high accuracy and robustness of the thermodynamic model. This study provides precise quantitative evidence from both thermodynamic and kinetic perspectives, validating the potential of spirooxindole diketopiperazine derivatives as promising candidates for novel agricultural fungicides.

### 2.5. Molecular Docking Analysis of Spirooxindole-Diketopiperazine Derivatives

Succinate dehydrogenase (SDH) and myosin are key proteins involved in fungal energy metabolism and cellular processes, respectively. To obtain preliminary insights into the potential interactions between the synthesized spirooxindole-diketopiperazine derivatives and these proteins, molecular docking studies were performed using SDH (PDB ID: 1ZOY) and myosin (PDB ID: 6UI4) as target models. The calculated binding free energies (ΔG) and docking results are summarized in Table 3.

The docking results revealed that the tested compounds exhibited different predicted binding affinities toward the selected targets, and the interaction profiles were influenced by structural modifications of the spirooxindole-diketopiperazine scaffold. In the myosin docking model (PDB ID: 6UI4), the primed derivatives generally showed more favorable binding energies than their corresponding analogues. Among them, compound **4d′** exhibited the optimal predicted interaction with myosin (ΔG = −6.40 kcal/mol), followed by **4c′** (ΔG = −6.18 kcal/mol) and **4b′** (ΔG =−5.68 kcal/mol). Similarly, in the SDH docking model (PDB ID: 1ZOY), compound **4c′** displayed the most favorable binding energy (ΔG = −6.91 kcal/mol), while **4b′** and **4d′** also showed relatively strong interactions with ΔG values of −6.60 and −6.65 kcal/mol, respectively.

Notably, the improved docking scores of these derivatives showed a broadly consistent trend with their observed antifungal activities, suggesting that the introduction of specific substituents and optimization of the diketopiperazine scaffold may influence ligand–target interactions. Among the tested derivatives, compound **4c′** exhibited the most favorable predicted binding energy toward SDH (ΔG = −6.91 kcal/mol). This result suggests that the structural features of **4c′** may promote favorable interactions within the SDH binding pocket, which may contribute to its observed antifungal activity.

**Figure 5:**
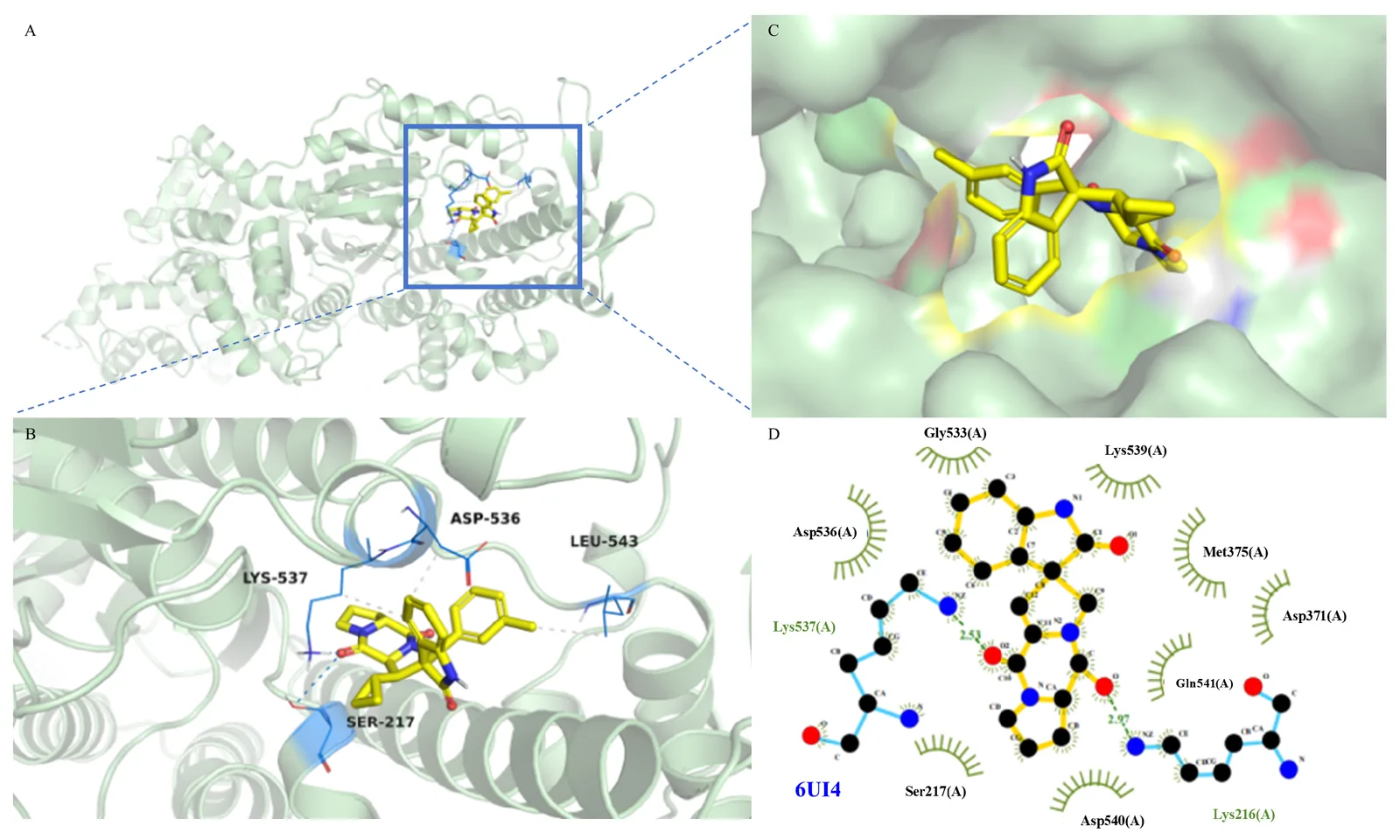
Molecular docking analysis of compound **4c′** with myosin (PDB ID: 6UI4). (**A**) overall docking pose of **4c′** within the myosin binding pocket; (**B**) three-dimensional interaction mode of **4c′** with surrounding amino acid residues; (**C**) surface representation of the **4c′**-myosin complex; (**D**) two-dimensional interaction diagram showing the interactions between **4c′** and myosin residues.

Compound **4c′** was specifically selected for detailed molecular docking visualization because it demonstrated the highest theoretical binding affinity (lowest binding free energy) against both target proteins in the computational evaluation. While compounds **4e** and **4e′** exhibited broader in vitro antifungal activity, it is well established that molecular docking models the interactions with isolated target proteins, whereas in vitro MIC assays reflect the comprehensive whole-cell antifungal efficacy. Therefore, **4c′** was utilized as the optimal structural model to elucidate the specific protein–ligand interactions within the target binding pockets. To further understand the molecular basis underlying this favorable docking performance, the binding mode of **4c′** with myosin was analyzed in detail (Figure 5). Compound **4c′** adopted a favorable orientation within the binding pocket of myosin, where the spirooxindole-diketopiperazine scaffold was accommodated in the hydrophobic region of the pocket. The carbonyl groups of the diketopiperazine moiety were involved in polar interactions with surrounding residues, which may contribute to the stabilization of the ligand–protein complex. Hydrophobic interactions with residues such as Leu543, together with additional polar contacts involving Ser217 and Asp536, were observed.

The two-dimensional interaction analysis further revealed that the carbonyl groups of the diketopiperazine ring formed hydrogen-bond interactions with Lys216 and Lys537, with calculated distances of 2.53 and 2.97 Å, respectively (Figure 5D). These hydrogen-bond interactions, combined with hydrophobic contacts, may contribute to the favorable binding affinity of the **4c′**-myosin complex.

**Figure 6:**
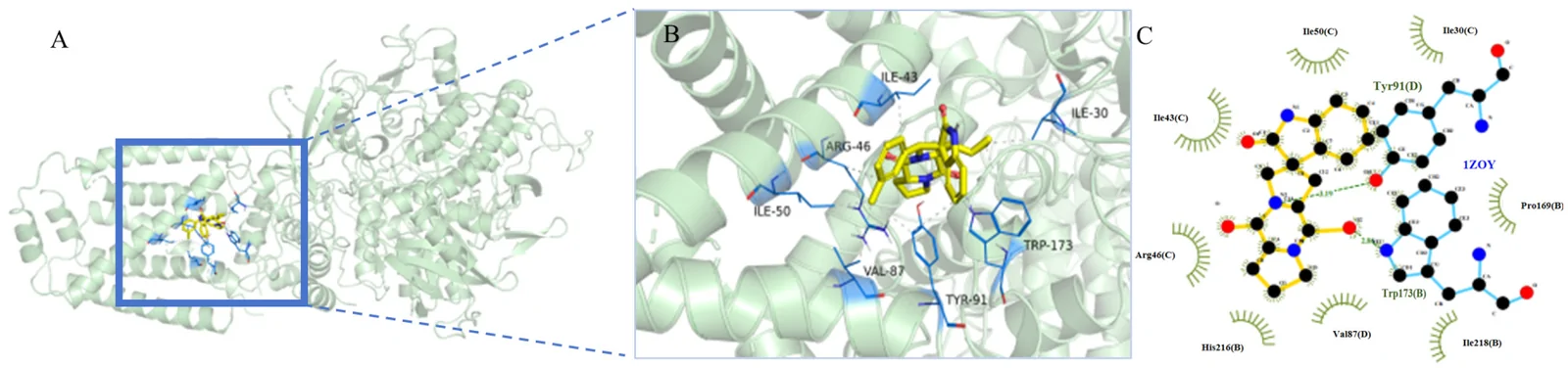
Molecular docking analysis of **4c′** with succinate dehydrogenase (SDH, PDB ID: 1ZOY): (**A**) overall view of the **4c′**–SDH complex; (**B**) close-up view of the binding site; and (**C**) two-dimensional interaction diagram.

Furthermore, the interaction modes of **4c′** with SDH was analyzed in detail (Figure 6). Compound **4c′** exhibited a favorable predicted binding energy toward SDH (ΔG = −6.91 kcal/mol). The docking analysis suggested that **4c′** adopted a favorable orientation within the SDH binding pocket and formed potential intermolecular interactions with surrounding residues. These structural features may contribute to the predicted interaction of **4c′** with SDH.

Overall, the molecular docking results suggested that the spirooxindole-diketopiperazine derivatives, particularly **4c′** and related analogues, exhibited favorable predicted interactions toward SDH and myosin. These findings provide preliminary molecular insights into possible ligand–target binding modes and may contribute to a better understanding of their structure–activity relationships.

It is noteworthy that molecular docking simulations provide theoretical predictions of ligand–target interactions rather than direct experimental evidence. Therefore, the proposed binding modes and potential antifungal mechanisms require further validation through biochemical and genetic investigations. Nevertheless, the docking results offer valuable structural insights into the interaction characteristics of these compounds with their potential targets.

## 3. Materials and Methods

### 3.1. General Information

^1^H and ^13^C NMR spectra were recorded on JEOL ECP 500 (400 MHz) or Bruker AVANCE 600 (Bruker Switzerland AG, Fällanden, Switzerland) NMR spectrometers. High-resolution mass spectrometry (HRMS) data were acquired using a Bruker Impact HD Q-TOF mass spectrometer (Milford, MA, USA). Single-crystal X-ray diffraction (SC-XRD) data were collected on a Bruker D8 VENTURE dual-wavelength (Mo/Cu) diffractometer (Bruker AXS, Karlsruhe, Germany). Melting points were determined with an X-4 digital display microscopic melting point apparatus (Beijing Tek Instruments Co., Ltd., Beijing, China). Unless otherwise specified, all chemical reagents and solvents purchased from commercial sources were used directly without further purification.

### 3.2. General Procedure for the Synthesis of Compound ***1***

To a 100 mL round-bottom flask, 2-oxindole (10 mmol) and anhydrous ethanol (15 mL) were added and stirred until fully dissolved. Subsequently, cyclopropanecarbox-aldehyde (11 mmol) and piperidine (15 mmol) were added. The reaction mixture was stirred and refluxed at 80 °C for 8 h. After completion of the reaction, the solvent was removed under reduced pressure using a rotary evaporator. The residue was purified by silica gel column chromatography to afford compound **1**.

### 3.3. General Procedure for the Synthesis of Compound ***2a***–***2g***

To a 250 mL round-bottom flask, glycine methyl ester hydrochloride (22 mmol), anhydrous MgSO_4_ (40 mmol), DCM (50 mL), and triethylamine (22 mmol) were sequentially added, and the mixture was stirred at room temperature for 1 h. Subsequently, the corresponding aldehyde (20 mmol) was added, and the mixture was kept stirring at room temperature for another 24 h. Upon completion, the mixture was filtered, diluted with water (30 mL), and separated into layers. The aqueous layer was extracted with DCM (3 × 10 mL). The organic phases were combined, washed with brine, and dried over anhydrous MgSO_4_. After filtration, the solvent was evaporated under reduced pressure. The crude products were used directly in the next step without further purification.

### 3.4. General Procedure for the Synthesis of Compound ***3a***–***3g***

Under a nitrogen atmosphere, Ag_2_O (12 mg, 0.05 mmol), compound **1** (0.1 mmol), compound **2a**–**2g** (0.2 mmol), Et_3_N (2.2 μL, 0.015 mmol), and diethyl ether (1 mL) were sequentially charged into a Schlenk tube to obtain a yellow suspension. The reaction mixture was stirred at 0 °C for 12 h, during which the color of the suspension completely shifted from yellow to dark gray. The solvent was removed under reduced pressure, and the crude residue was purified by silica gel column chromatography (petroleum ether/ethyl acetate = 2:1, *v*/*v*) to furnish the desired product **3a**–**3g**.

### 3.5. General Procedure for the Synthesis of Compound ***4a***–***4g***, ***4a′***–***4g′***

To a 25 mL round-bottom flask, DCM (5 mL) and saturated aqueous Na_2_CO_3_ solution (5 mL) were added to form a biphasic system. Compound **3a**–**3g** (0.1 mmol) was added under stirring until the solid was completely dissolved. Subsequently, a solution of Fmoc-L-Pro-Cl (0.1 mmol) in DCM (2 mL) was added dropwise. After stirring at room temperature for 6 h, the mixture was allowed to stand for phase separation. The aqueous phase was extracted with DCM (3 × 5 mL). The combined organic layers were washed with brine, dried over anhydrous MgSO_4_, and filtered. Piperidine (0.12 mmol) was then introduced into the filtrate, and the mixture was stirred at room temperature for an additional 1 h. Finally, the solvent was concentrated, and the residue was purified by column chromatography (petroleum ether/ethyl acetate = 1:1, *v*/*v*) to afford the products **4a**–**4g** and **4a′**–**4g′** as white solids.

### 3.6. Tested Fungal Strains and Medium Preparation

A total of twelve phytopathogenic fungal strains were employed in this study; specific strain information is provided in the Supplementary Materials. All strains were provided by the Laboratory of Natural Product Chemistry at Shaanxi University of Science and Technology.

### 3.7. Primary Antifungal Activity Screening

The antifungal activity screening methods are detailed in the Supplementary Materials.

### 3.8. Determination of Minimum Inhibitory Concentration (MIC) via Two-Fold Serial Dilution

The minimum inhibitory concentration (MIC) of the seven spirooxindole intermediates and fourteen final products against the target fungal strains were determined using the standard two-fold serial dilution method. Detailed experimental procedures are provided in the Supplementary Materials.

### 3.9. Isothermal Microcalorimetry (IMC) Assay

Isothermal microcalorimetry (IMC) was used to monitor the metabolic heat flow of the test strain, *Valsa mali.* Detailed IMC experimental procedures are provided in the Supplementary Materials.

### 3.10. Molecular Docking Simulations

The two target protein structures (PDB codes: 1ZOY and 6UI4) were retrieved from the RCSB Protein Data Bank (https://www.rcsb.org/). The protein structures were preprocessed using PyMOL. The three-dimensional structures of the synthesized small molecules were constructed based on their experimentally determined chemical structures using ChemDraw (version 21.0.0). The generated structures were converted into MOL2 format using OpenBabel (version 3.2.1) and subsequently optimized with AutoDockTools by adding hydrogen atoms and assigning partial charges to generate the ligand PDBQT files. Molecular docking was performed with AutoDock 4.2.6. The estimated binding free energies were obtained from the AutoDock docking calculations, and the lowest-energy conformation from the largest cluster was selected as the optimal binding mode. The three-dimensional visualizations were generated using PyMOL (version 0.99; DeLano Scientific, San Carlos, CA, USA), and the two-dimensional interaction diagrams were produced with LigPlot (version 2.2).

## 4. Conclusions

In summary, a series of novel spirooxindole-diketopiperazine derivatives were synthesized and systematically evaluated as potential antifungal agents for agricultural applications. The experimental results revealed clear structure–activity relationships (SARs), demonstrating that antifungal activity was mainly influenced by the electronic properties and substitution positions of the aromatic substituents, while stereoconfiguration showed a relatively limited effect under the tested conditions. In general, electron-withdrawing groups exhibited enhanced antifungal activity compared with electron-donating groups. Regarding substitution patterns, *ortho*-substitution was favorable for electron-withdrawing groups, whereas *meta*-substitution showed better activity for electron-donating groups. Furthermore, isothermal microcalorimetry and molecular docking analyses provided preliminary insights into the possible interaction mechanisms of these derivatives with myosin and succinate dehydrogenase (SDH), suggesting that hydrogen bonding and hydrophobic interactions may contribute to their antifungal activity. Several highly active derivatives displayed comparable or superior antifungal efficacy to the positive control ketoconazole, highlighting the potential of the spirooxindole-diketopiperazine scaffold as a promising framework for the development of novel agricultural fungicides.

## Figures and Tables

**Table 1:** Minimum inhibitory concentration (MIC) of compounds **3a**–**3g**, **4a**–**4g**, and **4a′**–**4g′**.

MIC (µg/mL)												
**Compound**	Plant Pathogenic Fungi ^a^											
	*M.m.*	*F.g.*	*F.o.n.*	*C.l.*	*A.a.*	*P.o.*	*A.b.*	*P.p*	*A.s.*	*C.g.*	*V.m.*	*F.o.v.*
**3a**	64	128	64	32	32	128	16	8	64	128	128	64
**3b**	64	128	64	64	8	64	64	32	32	32	32	256
**3c**	64	64	8	128	32	32	64	16	128	64	64	128
**3d**	128	128	64	32	32	16	8	16	64	256	32	128
**3e**	128	64	128	32	8	32	32	8	128	128	32	64
**3f**	64	128	32	16	16	8	32	32	64	128	16	128
**3g**	32	256	64	32	64	32	8	16	32	128	64	128
**4a**	64	32	16	16	32	16	16	8	32	64	4	32
**4b**	32	32	8	32	8	16	8	16	8	32	16	128
**4c**	64	16	32	64	16	32	4	16	4	16	8	64
**4d**	128	8	8	8	8	32	16	8	8	64	8	64
**4e**	32	32	32	16	8	8	32	4	16	64	8	32
**4f**	64	16	8	8	16	32	8	8	32	16	16	64
**4g**	64	32	32	32	8	8	32	16	8	32	4	32
**4a′**	32	16	64	16	16	8	16	8	32	64	8	128
**4b′**	64	32	8	32	8	32	4	8	32	128	16	64
**4c′**	16	16	32	32	8	32	8	32	8	64	8	32
**4d′**	64	32	16	8	32	16	16	16	16	64	16	64
**4e′**	64	16	32	16	8	16	8	8	32	64	4	32
**4f′**	32	32	8	16	16	32	16	8	16	32	32	64
**4g′**	64	32	8	32	16	32	8	16	32	64	8	64
ketoconazole	32	16	8	16	8	16	8	8	16	32	8	32

^a^ ***M.m.***, *Mycosphaerella melonis*; ***F.g.***, *Fusarium graminearum*; ***F.o.n***., *Fusarium oxysporum* f. sp. *niveum*; ***C.l.***, *Curvularia lunata*; ***A.a.***, *Alternaria alternata*; ***P.o.***, *Pyricularia oryzae*; ***A.b.***, *Alternaria brassicae*; ***P.p.***, *Physalospora piricola*; ***A.s.***, *Alternaria solani*; ***C.g.***, *Colletotrichum gloeosporioides*; ***V.m.***, *Valsa mali*; ***F.o.v.***, *Fusarium oxysporum* f. sp. *vasinfectum*.

**Table 2:** Fungal inhibition and kinetics parameters obtained by isothermal microcalorimetry.

Fungal Species	*C* (μg /mL)	*µ* (h^−1^)	*R* ^2^	*P_m_* (µW)	*t_m_* (h)
*Valsa mali*	0	0.018 ± 0.001	0.83	835.20 ± 12.8	43.92 ± 1.5
	1	0.022 ± 0.002	0.81	822.31 ± 8.7	51.23 ± 2.1
	2	0.017 ± 0.001	0.84	790.44 ± 10.2	53.38 ± 2.3
	4	0.017 ± 0.001	0.84	694.82 ± 9.8	61.69 ± 2.5
	8	0.018 ± 0.001	0.87	549.53 ± 11.5	70.48 ± 2.8

*µ*: fungal growth rate constant; *R*^2^: coefficient of determination; *P_m_*: maximum metabolic power output; *t_m_*: peak time. Data for *μ*, *P_m_*, and *t_m_* are presented as mean ± standard deviation (SD) from three independent measurements. *R*^2^ values represent the coefficient of determination obtained from curve fitting.

**Table 3:** Molecular docking and binding energy of compounds to different proteins.

ΔG (kcal/mol)							
PDB ID	Compound						
	4a	4b	4c	4d	4e	4f	4g
6UI4	−4.74	−4.08	−5.26	−4.57	−5.00	−4.90	−4.60
1ZOY	−5.41	−5.22	−5.67	−5.06	−5.74	−6.27	−5.37
	**4a′**	**4b′**	**4c′**	**4d′**	**4e′**	**4f′**	**4g′**
6UI4	−4.74	−5.68	−6.18	−6.40	−5.17	−4.50	−5.14
1ZOY	−6.08	−6.60	−6.91	−6.65	−5.79	−5.11	−5.80

## Data Availability

All data supporting the findings of this study are available within the paper and within its Supplementary Materials published online.

## Supplementary Materials

The following supporting information can be downloaded at: https://www.mdpi.com/article/10.3390/molecules31213846/s1.
