# Supplementary material for: Design, Synthesis, and Structure–Activity Relationship Studies of Spirotryprostatin A-Inspired Spirooxindole-Diketopiperazine Derivatives with Antifungal Activity
Source: Molecules. 2026 Oct 25;31(21):3846. doi: 10.3390/molecules31213846 (PMC13765499; doi:10.3390/molecules31213846)
Supplement: Supplementary file 1 [file molecules-31-03846-s001.zip › molecules-4601778-supplementary.pdf]

Supplementary Materials

Design, Synthesis, and Structure-Activity Relationship Studies of Spirotryprostatin A-Inspired Spirooxindole-Diketopiperazine Derivatives with Antifungal Activity

Murong Zhan, Yangmin Ma *, GeGe Dang, Zhichao Wang *, Menghua Shang, Qian Jiao

Key Laboratory of Chemical Additives for China National Light Industry, College of Chemistry and Chemical Engineering, Shaanxi University of Science and Technology, Xi’an, Shaanxi 710021, China
*Corresponding author
E-mail addresses: mayangmin@sust.edu.cn (Y.M.); 5264@sust.edu.cn (Z.W.)

content

1. Tested Fungal Strains and Medium Preparation 2
2. Primary Antifungal Activity Screening..2
2.1. Preparation of Fungal Suspensions 2
2.2. Preparation of Test Solutions 3
2.3. Agar Well Diffusion Assay..3
3. Determination of Minimum Inhibitory Concentration (MIC) via Two-Fold Serial Dilution 4
4. Isothermal Microcalorimetry (IMC) Assay 5
5. Characterization data of compounds 3a-3g..6
6. Characterization data of compounds 4a-4g and 4a'-4g' 9
7. ^{1}H NMR and ^{13}C NMR Spectra of compounds..15
8. Heat map of the antifungal effect of different tested compounds on different pathogens 36
9. X-ray crystallography data for 4a and 4a' 37

1. Tested Fungal Strains and Medium Preparation

A total of twelve phytopathogenic fungal strains were employed in this study, including *Mycosphaerella melonis* (*M. melonis*), *Fusarium graminearum* (*F. graminearum*), *Fusarium oxysporum* f. sp. *niveum* (*F. oxysporum* f. sp. *niveum*), *Curvularia lunata* (*C. lunata*), *Alternaria alternata* (*A. alternata*), *Pyricularia oryzae* (*P. oryzae*), *Alternaria brassicae* (*A. brassicae*), *Physalospora piricola* (*P. piricola*), *Alternaria solani* (*A. solani*), *Colletotrichum gloeosporioides* (*C. gloeosporioides*), *Valsa mali* (*V. mali*), and *Fusarium oxysporum* f. sp. *vasinfectum* (*F. oxysporum* f. sp. *vasinfectum*). All strains were provided by the Laboratory of Natural Product Chemistry at Shaanxi University of Science and Technology.

Medium Preparation: Potato dextrose agar (PDA) medium was prepared by dissolving 24 g of commercial PDA powder in 1000 mL of distilled water. The mixture was heated to boiling under constant stirring until completely dissolved. The resulting solution was dispensed into appropriate containers and autoclaved at 120 °C for 30 min. After cooling to approximately 60 °C, the medium was poured into sterile Petri dishes inside a laminar flow hood.

2. Primary Antifungal Activity Screening

2.1. Preparation of Fungal Suspensions

Under sterile conditions within a laminar flow hood, a small amount of mycelium from each of the twelve fungal strains was inoculated into a liquid medium using a sterile inoculating loop. The flasks were sealed with breathable membranes and incubated in a horizontal shaking incubator at 28 °C and 200 rpm for 24 h. The resulting cultures were filtered to separate the mycelial fragments, and the spore concentration of the filtrate was determined using a hemocytometer. The suspension

was subsequently diluted with sterile physiological saline to achieve a final standardized stock concentration of 1×10^6 CFU/mL.

2.2. Preparation of Test Solutions

The test compounds were accurately weighed and initially dissolved in pure dimethyl sulfoxide (DMSO) to form concentrated stock solutions. These solutions were subsequently diluted with sterile physiological saline to achieve a working concentration of 2 mg/mL. This step ensured that the final DMSO concentration in the test solutions was maintained at exactly 1% (v/v) to avoid any solvent-induced fungal toxicity. Ketoconazole (2 mg/mL) was prepared following the same procedure as the positive control, while sterile saline containing 1% DMSO was utilized as the negative control.

2.3. Agar Well Diffusion Assay

For each strain, 200 μL of the prepared fungal suspension was uniformly inoculated onto the surface of the agar medium using the spread-plate method. Wells with a diameter of 6 mm were punched into the agar layer using a sterile stainless steel borer, ensuring the underlying medium remained intact. The test solutions were then introduced into the wells, ensuring complete absorption into the agar. The Petri dishes were incubated in a constant-temperature incubator at 28 °C for 24 h. Afterward, the formation of inhibition zones was observed, and their diameters were precisely measured.

3. Determination of Minimum Inhibitory Concentration (MIC) via Two-Fold Serial Dilution

The minimum inhibitory concentration (MIC) values of the seven spirooxindole intermediates and fourteen final products against the target fungal strains were determined using the standard two-fold serial dilution method. Briefly, the positive control (ketoconazole) and the test compounds were dissolved in a minimum volume of DMSO. Meanwhile, the 96-well plates were pre-sterilized via ultraviolet (UV) irradiation inside the laminar flow hood. The DMSO stock solutions were then diluted with sterile Potato Dextrose Broth (PDB) to prepare a working solution with a concentration of 1024 μg/mL, ensuring the final DMSO concentration remained below 1% (v/v) to avoid solvent toxicity.

The assay was performed in sterile 96-well microtiter plates. Initially, 100 μL of freshly sterilized PDB medium was pre-dispensed into wells from column 2 to 10 for the dilution gradients. For the controls, column 11 served as the growth control (medium and fungal inoculum only), column 12 acted as the solvent control (medium containing 1% DMSO and fungal inoculum), and a separate set of wells was designated as the blank control (medium and test compounds only, without inoculum) to correct for any background turbidity from the chemicals.

Subsequently, 200 μL of the 1024 μg/mL working solution was introduced into the first well (column 1). After thorough mixing, 100 μL of the solution from column 1 was transferred to column 2. This two-fold serial dilution process was repeated sequentially up to column 10, from which the final 100 μL was discarded to maintain an equal volume of 100 μL in all test wells. Finally, 100 μL of a standardized fungal spore suspension was inoculated into columns 1 through 12 (excluding the blank control). This brought the total working volume to 200 μL per well and perfectly established a final test concentration gradient ranging from 512 to 1 μg/mL. Each compound was tested in triplicate to ensure reproducibility, and each plate was

inoculated with a single fungal strain to eliminate cross-contamination.

The inoculated plates were incubated in a constant-temperature incubator at 28 °C for 48 h. The MIC values were determined through visual inspection, defined as the lowest concentration of the compound that completely inhibited visible macroscopic fungal growth (i.e., the absence of visible mycelial clumps or turbidity) when compared to the untreated growth control.

4. Isothermal Microcalorimetry (IMC) Assay

The phytopathogenic fungus *Valsa mali* was selected as the test strain, which was maintained on PDA slants and stored in a refrigerator at 4 °C until use. Under sterile conditions, an appropriate amount of mycelium from the activated culture was transferred into liquid medium, sealed with a breathable membrane, and incubated in a shaking incubator at 28 °C and 200 rpm for 24 h. The fungal density was determined using a hemocytometer and subsequently diluted with sterile water to a final working concentration of 1×10^6 CFU/mL.

The test compounds were dissolved in DMSO to prepare a range of gradient concentrations (0-8 μg/mL). Thermal kinetic behaviors were monitored using a TAM III isothermal microcalorimeter. Under sterile conditions, the fungal suspension and the test compound solutions were introduced into glass ampoules (experimental groups); an equivalent volume of the corresponding solvent-containing medium without test compounds was used as the control group. All treatments were performed in triplicate. After sealing, the ampoules were placed into the isothermal sample cells at 28 °C to continuously monitor the heat flow signals. Data were collected at a sampling frequency of once every 10 s, and the monitoring was maintained for 3-7 days until the baseline stabilized.

The growth rate constant (μ), maximum heat-flow power (P_m), and time to reach the maximum heat-flow power (t_m) were calculated from the integrated heat-flow curves to evaluate the inhibitory effects of the test compounds. The values of μ, Pm, and *tm* obtained from the replicate experiments were analyzed by one-way analysis of variance using SPSS software, with $p < 0.05$ considered statistically significant. Strict aseptic techniques were maintained throughout the experiment, and control assays

confirmed that the solvent (1% DMSO) exerted no significant interference on fungal growth.

5. Characterization data of compounds 3a-3g

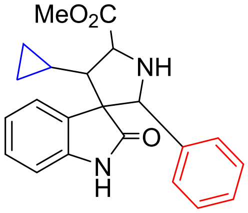

Methyl 4′-cyclopropyl-2-oxo-2′-phenylspiro[indoline-3,3′-pyrrolidine]-5′-carboxylate (3a) (diastereomeric mixture): White solid, 32.6 mg, 90% yield, R_f =0.33 (petroleum ether/ethyl acetate =2/1), m.p.: 190~191°C. **^{1}H NMR** (600 MHz, $CDCl_3$) δ 8.65 (s, 1H), 7.89 (d, J = 7.5 Hz, 1H), 7.66 (t, J = 8.2 Hz, 1H), 7.57 – 7.50 (m, 4H), 7.38 (d, J = 7.4 Hz, 2H), 7.15 (d, J = 7.7 Hz, 1H), 5.04 (s, 1H), 4.51 (d, J = 7.5 Hz, 1H), 4.28 (s, 3H), 3.90 (s, 1H), 2.55 – 2.51 (m, 1H), 1.42 (s, 1H), 1.04 – 0.96 (m, 1H), 0.73 – 0.66 (m, 1H), 0.63 – 0.57 (m, 1H), 0.24 – 0.19 (m, 1H). **^{13}C NMR** (151 MHz, $CDCl_3$) δ 180.3, 172.9, 141.2, 136.2, 129.9, 127.9, 127.7, 126.2, 125.2, 122.1, 109.7, 72.6, 67.0, 63.5, 59.5, 52.4, 13.7, 5.8, 4.2. **HRMS** (ESI, m/z): $C_{22}H_{23}N_2O_3$ $[M+H]^+$, Calculated mass: 363.1703; found: 363.1702.

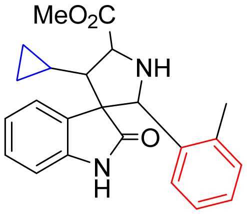

Methyl 4′-cyclopropyl-2-oxo-2′-(2-methylphenyl)spiro[indoline-3,3′-pyrrolidine]-5′-carboxylate (3b) (diastereomeric mixture): White solid, 28.2 mg, 75% yield, R_f =0.30 (petroleum ether/ethyl acetate =2/1), m.p.: 197~198°C. **^{1}H NMR** (400 MHz, $CDCl_3$) δ 8.37 (s, 1H), 7.85 (d, *J* = 7.9 Hz, 1H), 7.64 (d, *J* = 7.5 Hz, 1H), 7.38 – 7.29 (m, 2H), 7.25 – 7.19 (m, 2H), 7.07 (d, *J* = 7.5 Hz, 1H), 6.89 (d, *J* = 7.8 Hz, 1H), 5.16 (s, 1H), 4.37 (d, *J* = 9.0 Hz, 1H), 4.01 (s, 3H), 2.37 – 2.31 (m, 1H), 1.92 (s, 3H), 1.45 – 1.36 (m, 1H), 1.02 – 0.86 (m, 1H), 0.69 – 0.58 (m, 1H), 0.37 – 0.23 (m, 2H), 0.08 – 0.00 (m, 1H). **^{13}C NMR** (101 MHz, $CDCl_3$) δ 181.1, 172.9, 140.3, 136.0, 135.2, 130.5, 130.2, 128.3, 127.6, 127.1, 125.9, 125.1, 122.3, 109.8, 67.8, 66.2, 63.0, 59.2, 52.5, 19.5, 10.8, 4.2, 3.3. **HRMS** (ESI, m/z): $C_{23}H_{25}N_2O_3$ $[M+H]^+$, Calculated mass: 377.1860; found: 377.1868.

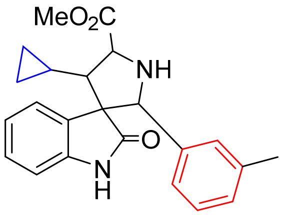

Methyl 4′-cyclopropyl-2-oxo-2′-(3-methylphenyl)spiro[indoline-3,3′-pyrrolidine]-5′-carboxylate (3c) (diastereomeric mixture): White solid, 32.4 mg, 86% yield, R_f =0.32 (petroleum ether/ethyl acetate =2/1), m.p.: 191~192°C. **^{1}H NMR** (600 MHz, $CDCl_3$) δ 7.76 – 7.69 (m, 2H), 7.49 – 7.46 (m, 1H), 7.38 – 7.35 (m, 1H), 7.26 – 7.19 (m, 2H), 7.04 (s, 1H), 7.00 – 6.93 (m, 2H), 4.84 (s, 1H), 4.32 (d, J = 7.8 Hz, 1H), 4.11 (s, 3H), 2.43 (s, 3H), 2.38 – 2.34 (m, 1H), 1.52 (s, 1H), 1.29 – 1.21 (m, 1H), 0.87 – 0.80 (m, 1H), 0.56 – 0.50 (m, 1H), 0.46 – 0.40 (m, 1H), 0.09 – 0.02 (m, 1H). **^{13}C NMR** (101 MHz, $CDCl_3$) δ 180.2, 172.9, 141.2, 137.5, 136.0, 130.0, 128.5, 128.0, 127.8, 126.9, 125.3, 123.2, 122.2, 109.6, 72.6, 67.1, 63.4, 59.6, 52.4, 21.4, 13.0, 4.8, 4.3. **HRMS** (ESI, m/z): $C_{23}H_{25}N_2O_3$ $[M+H]^+$, Calculated mass: 377.1860; found: 377.1864.

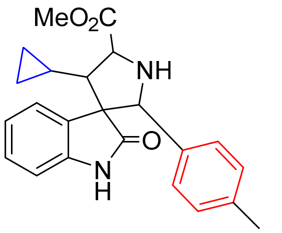

Methyl 4′-cyclopropyl-2-oxo-2′-(4-methylphenyl)spiro[indoline-3,3′-pyrrolidine]-5′-carboxylate (3d) (diastereomeric mixture): White solid, 32.0 mg, 85% yield, R_f =0.35 (petroleum ether/ethyl acetate =2/1), m.p.: 189~190°C. **^{1}H NMR** (600 MHz, $CDCl_3$) δ 8.74 (s, 1H), 7.74 (d, J = 7.4 Hz, 1H), 7.51 (t, J = 7.6 Hz, 1H), 7.39 (t, J = 7.5 Hz, 1H), 7.20 – 7.09 (m, 4H), 7.00 (d, J = 7.0 Hz, 1H), 4.89 (s, 1H), 4.37 (d, J = 7.6 Hz, 1H), 4.14 (s, 3H), 2.47 (s, 3H), 2.41 – 2.35 (m, 1H), 1.26 (s, 1H), 1.19 (t, J = 7.0 Hz, 1H), 0.85 (s, 1H), 0.57 – 0.51 (m, 1H), 0.45 (s, 1H), 0.11 – 0.02 (m, 1H). **^{13}C NMR** (101 MHz, $CDCl_3$) δ 190.5, 172.9, 141.0, 137.4, 133.1, 128.7, 128.7, 128.0, 128.0, 126.1, 125.4, 125.3, 122.2, 109.5, 72.5, 67.1, 63.4, 59.6, 52.4, 21.1, 13.0, 4.8, 4.3. **HRMS** (ESI, m/z): $C_{23}H_{25}N_2O_3$ $[M+H]^+$, Calculated mass: 377.1860; found: 377.1862.

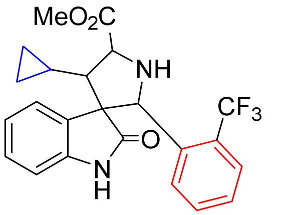

Methyl 4′-cyclopropyl-2-oxo-2′-[2-(trifluoromethyl)phenyl]spiro[indoline-3,3′-pyrrolidine]-5′-carboxylate (3e) (diastereomeric mixture): White solid, 28.8 mg, 67% yield, R_f =0.25

(petroleum ether/ethyl acetate =2/1), m.p.: 191~192°C. **^{1}H NMR** (600 MHz, $CDCl_3$) δ 8.41 (d, J = 7.9 Hz, 1H), 7.86 (s, 1H), 7.69 (t, J = 7.7 Hz, 1H), 7.54 (d, J = 7.8 Hz, 1H), 7.46 – 7.36 (m, 2H), 7.28 (d, J = 7.9 Hz, 1H), 7.14 (t, J = 7.5 Hz, 1H), 6.82 (d, J = 7.7 Hz, 1H), 5.07 (s, 1H), 4.35 (d, J = 10.4 Hz, 1H), 3.89 (s, 3H), 2.33 (t, J = 10.1 Hz, 1H), 0.99 – 0.81 (m, 1H), 0.37 – 0.27 (m, 2H), 0.15 – 0.06 (m, 3H). **^{13}C NMR** (101 MHz, $CDCl_3$) δ 177.6, 174.4, 140.4, 139.9, 132.1, 131.7, 128.3, 127.5, 127.3 (q, J = 39 Hz), 127.1, 126.7 (q, J = 274 Hz), 125.3 (q, J = 6 Hz), 123.7, 122.9, 109.5, 65.5, 64.9, 62.7, 57.7, 52.4, 8.0, 2.8, 1.9. **HRMS** (ESI, m/z): $C_{23}H_{22}F_3N_2O_3$ $[M+H]^+$, Calculated mass: 431.1577; found: 431.1579.

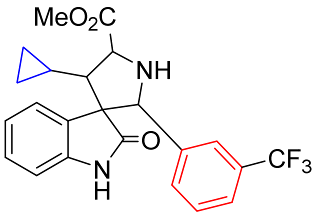

Methyl 4′-cyclopropyl-2-oxo-2′-[3-(trifluoromethyl)phenyl]spiro[indoline-3,3′-pyrrolidine]-5′-carboxylate (3f) (diastereomeric mixture): White solid, 30.1 mg, 70% yield, R_f =0.29 (petroleum ether/ethyl acetate =2/1), m.p.: 163~164°C. **^{1}H NMR** (600 MHz, $CDCl_3$) δ 8.56 (s, 1H), 7.74 (d, J = 7.5 Hz, 1H), 7.67 (d, J = 7.1 Hz, 1H), 7.54 – 7.47 (m, 3H), 7.43 – 7.40 (m, 2H), 7.03 (d, J = 7.8 Hz, 1H), 4.93 (s, 1H), 4.37 (d, J = 7.8 Hz, 1H), 4.14 (s, 3H), 2.42 – 2.37 (m, 1H), 1.27 – 1.20 (m, 2H), 0.89 – 0.83 (m, 1H), 0.58 – 0.53 (m, 1H), 0.49 – 0.42 (m, 1H), 0.09 – 0.04 (m, 1H). **^{13}C NMR** (101 MHz, $CDCl_3$) δ 179.7, 172.7, 141.0, 137.5, 130.2 (q, J = 40 Hz), 129.4 (q, J = 3 Hz), 128.5 (2C), 128.4, 125.2, 124.5 (q, J = 4 Hz), 123.9 (q, J = 273 Hz), 123.3 (q, J = 4 Hz), 122.5, 109.8, 71.9, 67.0, 63.3, 59.2, 52.5, 12.8, 4.8, 4.1. **HRMS** (ESI, m/z): $C_{23}H_{22}F_3N_2O_3$ $[M+H]^+$, Calculated mass: 431.1577; found: 431.1578.

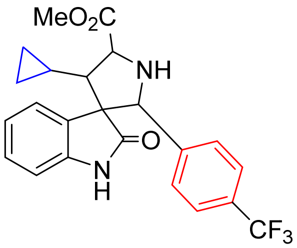

Methyl 4′-cyclopropyl-2-oxo-2′-[4-(trifluoromethyl)phenyl]spiro[indoline-3,3′-pyrrolidine]-5′-carboxylate (3g) (diastereomeric mixture): White solid, 33.6 mg, 78% yield, R_f =0.27 (petroleum ether/ethyl acetate=2/1); m.p.: 178~179°C. **^{1}H NMR** (600 MHz, $CDCl_3$) δ 8.58 (s, 1H), 7.72 (d, J = 7.5 Hz, 1H), 7.62 (d, J = 8.4 Hz, 2H), 7.54 – 7.49 (m, 1H), 7.41 – 7.34 (m, 3H), 7.00 (d, J = 7.7 Hz, 1H), 4.92 (s, 1H), 4.36 (d, J = 7.9 Hz, 1H), 4.11 (s, 3H), 2.41 – 2.35 (m, 1H), 1.52 (d, J = 4.5 Hz, 1H), 1.23 – 1.12 (m, 1H), 0.85 – 0.77 (m, 1H), 0.54 – 0.47 (m, 1H), 0.45 – 0.37 (m, 1H), 0.07 – 0.00 (m, 1H). **^{13}C NMR** (101 MHz, $CDCl_3$) δ 179.7, 172.8, 140.9, 140.7, 129.9 (q, J = 33 Hz), 129.6 (2C), 128.4, 126.7, 125.2, 125.0 (q, J = 4 Hz, 2C), 123.9 (q, J = 276 Hz), 122.5, 109.9, 71.8, 66.9, 63.3, 59.4, 52.5, 12.7, 4.7, 4.1. **HRMS** (ESI, m/z): $C_{23}H_{22}F_3N_2O_3$ $[M+H]^+$, Calculated mass: 431.1577; found: 431.1578.

6. Characterization data of compounds 4a-4g and 4a'-4g'

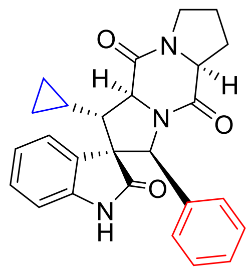

(3*R*,8*S*,9*S*,12*S*,18*S*)-8-cyclopropyl-18-phenyl-8,9,12,13,14,15-hexahydro-11*H*,17*H*,18*H*-spiro[bispyrrolo[1,2-a:1′,2′-d]pyrazine-3,3′-indoline]-2,11,17-trione (4a): White solid, 24.3 mg, 57% yield, R_f =0.33. (petroleum ether/ethyl acetate=1/1), m.p.: 197~198°C. **^{1}H NMR** (400 MHz, $CDCl_3$) δ 8.33 (s, 1H), 7.37 – 7.31 (m, 4H), 7.30 – 7.25 (m, 1H), 7.18 – 7.12 (m, 1H), 7.00 – 6.94 (m, 2H), 6.91 – 6.85 (m, 1H), 5.36 – 5.35 (m, 1H), 4.73 – 4.66 (m, 1H), 4.51 – 4.44 (m, 1H), 3.88 – 3.79 (m, 1H), 3.72 – 3.62 (m, 1H), 2.80 – 2.69 (m, 1H), 2.41 – 2.30 (m, 2H), 2.12 – 1.97 (m, 2H), 1.00 – 0.86 (m, 1H), 0.44 – 0.11 (m, 4H). **^{13}C NMR** (101 MHz, $CDCl_3$) δ 175.5, 167.3, 166.2, 140.3, 136.8, 132.0, 128.8 (2C), 128.5 (2C), 128.1 (2C), 123.2, 122.8, 110.4, 68.8, 64.3, 61.3, 61.0, 49.8, 45.7, 27.3, 24.0, 9.5, 3.6, 3.0. **HRMS** (ESI, m/z): $C_{26}H_{26}N_3O_3$ $[M+H]^+$, Calculated mass: 428.1969; found: 428.1969.

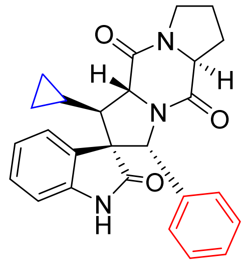

(3*S*,8*R*,9*R*,12*S*,18*R*)-8-cyclopropyl-18-phenyl-8,9,12,13,14,15-hexahydro-11*H*,17*H*,18*H*-spiro[bispyrrolo[1,2-a:1′,2′-d]pyrazine-3,3′-indoline]-2,11,17-trione (4a'): White solid, 17.9 mg, 42% yield, m.p.: 192~193°C, R_f =0.26. (petroleum ether/ethyl acetate=1/1). **^{1}H NMR** (400 MHz, $CDCl_3$) δ 8.61 (s, 1H), 7.35 – 7.29 (m, 4H), 7.25 (d, *J* = 7.5 Hz, 1H), 7.12 (t, *J* = 7.6 Hz, 1H), 6.97 – 6.92 (m, 2H), 6.87 (d, *J* = 7.5 Hz, 1H), 5.25 (s, 1H), 4.67 (d, *J* = 10.0 Hz, 1H), 4.45 (t, *J* = 7.7 Hz, 1H), 3.84 – 3.61 (m, 2H), 2.72 (t, *J* = 9.9 Hz, 1H), 2.35 – 2.29 (m, 2H), 2.05 – 1.97 (m, 2H), 0.89 (s, 1H), 0.42 – 0.12 (m, 4H). **^{13}C NMR** (101 MHz, $CDCl_3$) δ175.4, 167.3, 166.2, 140.2, 136.8, 132.0, 128.8 (2C), 128.5 (2C), 128.1 (2C), 123.2, 122.8, 110.4, 68.8, 64.2, 61.2, 61.0, 49.8, 45.6, 27.3, 24.0, 9.5, 3.6, 3.0. **HRMS** (ESI, m/z): $C_{26}H_{25}N_3NaO_3$ $[M+Na]^+$, Calculated mass: 450.1788; found: 450.1794.

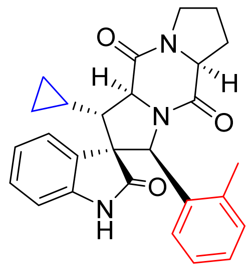

(3*R*,8*S*,9*S*,12*S*,18*S*)-8-cyclopropyl-18-(2-methylphenyl)-8,9,12,13,14,15-hexahydro-11*H*,17*H*,18*H*-spiro[bispyrrolo[1,2-a:1′,2′-d]pyrazine-3,3′-indoline]-2,11,17-trione (4b): White solid, 17.6 mg, 40% yield, m.p.: 184~185°C, R_f =0.32. (petroleum ether/ethyl acetate=1/1). **^{1}H NMR** (400 MHz, $CDCl_3$) δ 7.44 – 7.26 (m, 2H), 7.19 – 7.05 (m, 5H), 6.79 – 6.74 (m, 1H), 5.45 (s, 1H), 4.67 (d, *J* = 10.2 Hz, 1H), 4.44 (t, *J* = 7.7 Hz, 1H), 3.85 – 3.76 (m, 1H), 3.66 – 3.60 (m, 1H), 3.14 (t, J = 5.9 Hz, 1H), 2.71 (t, *J* = 9.9 Hz, 1H), 2.57 – 2.53 (m, 2H), 2.35 – 2.27 (m, 2H), 1.99 (s, 3H), 0.94 (t, *J* = 10.1 Hz, 1H), 0.39 – 0.10 (m, 4H). **^{13}C NMR** (151 MHz, $CDCl_3$) δ 173.2, 166.1, 165.1, 142.3, 133.9, 133.5, 130.2, 129.2, 127.8, 126.8, 125.2, 123.6, 121.8 (2C), 109.2, 64.0, 62.9, 59.9, 59.0, 51.1, 44.6, 26.3, 24.7, 23.0, 18.7, 8.0, 2.4. **HRMS** (ESI, m/z): $C_{27}H_{28}N_3O_3$ $[M+H]^+$, Calculated mass: 442.2125; found:442.2128.

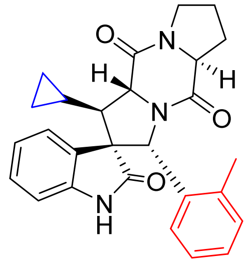

(3*S*,8*R*,9*R*,12*S*,18*R*)-8-cyclopropyl-18-(2-methylphenyl)-8,9,12,13,14,15-hexahydro-11*H*,17*H*,18*H*-spiro[bispyrrolo[1,2-a:1′,2′-d]pyrazine-3,3′-indoline]-2,11,17-trione (4b'): White solid, 14.6 mg, 33% yield, m.p.: 177~178°C, R_f =0.22. (petroleum ether/ethyl acetate=1/1). **^{1}H NMR** (600 MHz, $CDCl_3$) δ 7.28 (t, *J* = 7.7 Hz, 1H), 7.19 (d, *J* = 9.5 Hz, 2H), 7.11 – 7.03 (m, 4H), 7.01 – 6.95 (m, 1H), 6.69 – 6.63 (m, 1H), 4.57 (t, *J* = 9.1 Hz, 1H), 4.35 (t, *J* = 7.8 Hz, 1H), 3.74 – 3.68 (m, 1H), 3.57 – 3.51 (m, 1H), 3.02 (t, *J* = 5.8 Hz, 1H), 2.63 – 2.55 (m, 1H), 2.48 – 2.40 (m, 2H), 2.25 – 2.17 (m, 2H), 1.90 (s, 3H), 0.83 (d, *J* = 10.0 Hz, 1H), 0.29 – 0.01 (m, 4H). **^{13}C NMR**(151 MHz, $CDCl_3$) δ 173.2, 166.1, 165.1, 142.3, 133.9, 133.5, 130.2, 129.2, 127.8, 126.8, 125.2, 123.6, 121.8 (2C), 109.2, 64.0, 62.9, 60.4, 59.0, 51.1, 44.6, 26.3, 24.7, 23.0, 18.7, 8.0, 2.4. **HRMS** (ESI, m/z): $C_{27}H_{27}N_3NaO_3$ $[M+Na]^+$, Calculated mass: 464.1945; found: 464.1954.

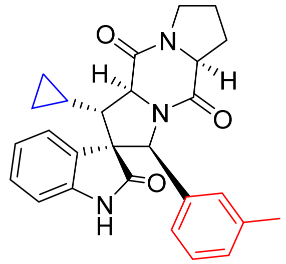

(3*R*,8*S*,9*S*,12*S*,18*S*)-8-cyclopropyl-18-(3-methylphenyl)-8,9,12,13,14,15-hexahydro-11*H*,17*H*,18*H*-spiro[bispyrrolo[1,2-a:1′,2′-d]pyrazine-3,3′-indoline]-2,11,17-trione (4c):

White solid, 23.8 mg, 54% yield, R_f =0.36. (petroleum ether/ethyl acetate=1/1), m.p.: 192~193°C. **^{1}H NMR** (400 MHz, $CDCl_3$) δ 8.56 (s, 1H), 7.33 – 7.08 (m, 6H), 6.89 – 6.85 (m, 1H), 6.72 (s, 1H), 4.66 (d, *J* = 10.3 Hz, 1H), 4.45 (t, *J* = 7.4 Hz, 1H), 3.84 – 3.75 (m, 1H), 3.69 – 3.62 (m, 1H), 2.73 (t, *J* = 9.2 Hz, 1H), 2.36 – 2.30 (m, 2H), 2.24 (s, 3H), 2.04 – 1.98 (m, 2H), 0.92 (s, 2H), 0.45 – 0.15 (m, 4H). **^{13}C NMR** (101 MHz, $CDCl_3$) δ 177.4, 165.4, 165.0, 140.8, 137.5, 132.8, 131.9, 129.0, 128.8, 128.7, 126.7, 124.1, 122.7, 110.4, 69.7, 62.6, 61.0, 54.2, 44.9, 44.6, 31.7, 22.7, 21.3, 14.2, 10.2, 5.9, 2.6. **HRMS** (ESI, m/z): $C_{27}H_{28}N_3O_3$ $[M+H]^+$, Calculated mass: 442.2125; found: 442.2127.

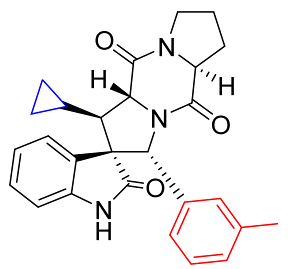

(3*S*,8*R*,9*R*,12*S*,18*R*)-8-cyclopropyl-18-(3-methylphenyl)-8,9,12,13,14,15-hexahydro-11*H*,17*H*,18*H*-spiro[bispyrrolo[1,2-a:1′,2′-d]pyrazine-3,3′-indoline]-2,11,17-trione (4c'): White solid, 16.8 mg, 38% yield, R_f =0.30. (petroleum ether/ethyl acetate=1/1), m.p.: 185~186°C. **^{1}H NMR** (400 MHz, $CDCl_3$) δ 7.54 – 7.32 (m, 2H), 7.30 – 7.07 (m, 5H), 6.76 – 6.67 (m, 2H), 4.73 – 4.65 (m, 1H), 4.51 – 4.46 (m, 1H), 3.88 – 3.78 (m, 1H), 3.71 – 3.62 (m, 1H), 2.86 – 2.73 (m, 1H), 2.59 (s, 1H), 2.39 – 2.34 (m, 2H), 2.33 – 2.28 (m, 3H), 2.09 – 1.97 (m, 2H), 1.03 – 0.87 (m, 1H), 0.45 – 0.25 (m, 2H), 0.22 – 0.10 (m, 2H). **^{13}C NMR** (101 MHz, $CDCl_3$) 173.9, 167.3, 166.3, 137.7, 136.7, 131.4, 129.2, 128.8, 128.5, 128.3, 123.4, 122.9, 115.2, 110.5, 69.1, 64.3, 61.0, 52.0, 50.0, 45.7, 27.3, 25.6, 24.0, 21.6, 9.5, 3.6, 3.3. **HRMS** (ESI, m/z): $C_{27}H_{27}N_3NaO_3$ $[M+Na]^+$, Calculated mass: 464.1945; found: 464.1953.

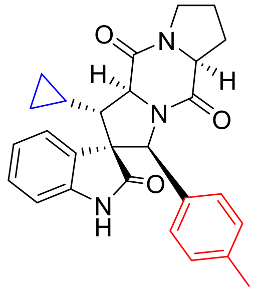

(3*R*,8*S*,9*S*,12*S*,18*S*)-8-cyclopropyl-18-(4-methylphenyl)-8,9,12,13,14,15-hexahydro-11*H*,17*H*,18*H*-spiro[bispyrrolo[1,2-a:1′,2′-d]pyrazine-3,3′-indoline]-2,11,17-trione (4d): White solid, 26.5 mg, 60% yield, R_f =0.34. (petroleum ether/ethyl acetate=1/1), m.p.: 189~190°C. **^{1}H NMR** (400 MHz, $CDCl_3$) δ 7.71 (s, 1H), 7.38 – 7.32 (m, 1H), 7.26 (d, J = 7.3 Hz, 1H), 7.15 – 7.07 (m, 3H), 6.91 (d, J = 7.8 Hz, 1H), 6.85 – 6.78 (m, 2H), 4.71 – 4.61 (m, 1H), 4.44 (t, J = 7.8 Hz, 1H), 3.86 – 3.74 (m, 1H), 3.68 – 3.58 (m, 1H), 2.80 – 2.69 (m, 1H), 2.53 (s, 1H), 2.34 (s, 1H), 2.32 (s, 3H), 2.04 – 1.97 (m, 2H), 0.92 (t, J = 6.9 Hz, 2H), 0.41 – 0.12 (m, 4H). **^{13}C NMR** (101 MHz, $CDCl_3$) δ 174.9, 167.2, 166.3, 142.5, 140.1, 137.7, 133.7, 132.1, 129.3, 128.8, 123.3, 122.8, 115.4, 110.0, 68.7, 64.2, 61.1, 61.0, 49.8, 46.2, 27.2, 25.7, 24.0, 21.2, 9.5, 3.6, 3.0. **HRMS** (ESI,

m/z): $C_{27}H_{28}N_3O_3$ $[M+H]^+$, Calculated mass: 442.2125; found: 442.2128.

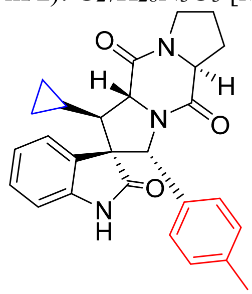

(3*S*,8*R*,9*R*,12*S*,18*R*)-8-cyclopropyl-18-(4-methylphenyl)-8,9,12,13,14,15-hexahydro-11*H*,17*H*,18*H*-spiro[bispyrrolo[1,2-a:1′,2′-d]pyrazine-3,3′-indoline]-2,11,17-trione (4d'): White solid, 20.7 mg, 47% yield, R_f =0.26. (petroleum ether/ethyl acetate=1/1), m.p.: 186~187°C. **^{1}H NMR** (400 MHz, $CDCl_3$) δ 9.40 (s, 1H), 7.61 (s, 1H), 7.46 (t, J = 7.7 Hz, 1H), 7.34 – 7.30 (m, 1H), 7.23 (t, J = 7.5 Hz, 1H), 7.11 – 7.06 (m, 2H), 6.82 (t, J = 9.2 Hz, 2H), 4.71 – 4.60 (m, 1H), 4.44 (t, J = 7.7 Hz, 1H), 3.85 – 3.73 (m, 1H), 3.67 – 3.58 (m, 1H), 2.32 (s, 3H), 2.04 – 1.95 (m, 3H), 1.92 – 1.89 (m, 2H), 1.02 – 0.86 (m, 2H), 0.49 – 0.29 (m, 2H), 0.13 – 0.02 (m, 2H). **^{13}C NMR** (101 MHz, $CDCl_3$) δ 167.3, 166.0, 138.2, 133.5, 130.8, 129.5(2C), 129.3(2C), 128.8, 124.2, 123.3, 122.8, 111.0, 69.2, 64.2, 61.0, 51.6, 50.3, 45.7, 44.6, 27.2, 23.9, 22.6, 21.3, 9.7, 3.7. **HRMS** (ESI, m/z): $C_{27}H_{27}N_3NaO_3$ $[M+Na]^+$, Calculated mass: 464.1945; found: 464.1949.

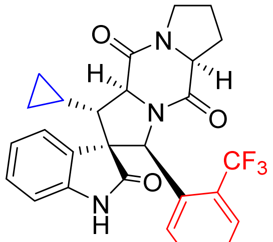

(3*R*,8*S*,9*S*,12*S*,18*S*)-8-cyclopropyl-18-[2-(trifluoromethyl)phenyl]-8,9,12,13,14,15-hexahydro-11*H*,17*H*,18*H*-spiro[bispyrrolo[1,2-a:1′,2′-d]pyrazine-3,3′-indoline]-2,11,17-trione (4e): White solid, 22.3 mg, 45% yield, R_f =0.28. (petroleum ether/ethyl acetate=1/1), m.p.: 198~199°C. **^{1}H NMR** (400 MHz, $CDCl_3$) δ 8.33 (s, 1H), 7.34 (d, *J* = 11.4 Hz, 3H), 7.30 – 7.26 (m, 1H), 7.18 – 7.12 (m, 1H), 6.97 (d, *J* = 3.6 Hz, 2H), 6.91 – 6.86 (m, 1H), 4.72 – 4.66 (m, 1H), 4.51 – 4.43 (m, 1H), 3.89 – 3.79 (m, 1H), 3.72 – 3.62 (m, 1H), 2.80 – 2.70 (m, 1H), 2.40 – 2.31 (m, 2H), 2.09 – 1.99 (m, 2H), 1.70 – 1.64 (m, 1H), 0.98 – 0.88 (m, 1H), 0.46 – 0.09 (m, 4H). **^{13}C NMR** (101 MHz, $CDCl_3$) δ 175.7, 166.9, 166.0, 140.5, 136.1, 132.0, 131.4, 129.0, 128.0, 127.7, 126.9, 126.1 (q, *J* = 5.1 Hz), 125.6, 122.9 (q, *J* = 11.1 Hz), 110.0, 63.9, 63.8, 60.9, 60.5, 53.5, 51.1, 45.7, 27.2, 23.9, 9.0, 3.4, 3.0. **HRMS** (ESI, m/z): $C_{27}H_{25}F_3N_3O_3$ $[M+H]^+$, Calculated mass: 496.1843; found: 496.1847.

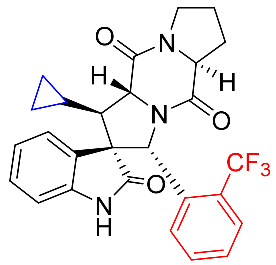

(3*S*,8*R*,9*R*,12*S*,18*R*)-8-cyclopropyl-18-[2-(trifluoromethyl)phenyl]-8,9,12,13,14,15-hexahydro-11*H*,17*H*,18*H*-spiro[bispyrrolo[1,2-a:1′,2′-d]pyrazine-3,3′-indoline]-2,11,17-trione (4e'): White solid, 17.8 mg, 36% yield, R_f =0.23. (petroleum ether/ethyl acetate=1/1), m.p.: 201~202°C. **^{1}H NMR** (400 MHz, $CDCl_3$) δ 8.02 (s, 1H), 7.64 (s, 1H), 7.56 (s, 1H), 7.44 (d, *J* = 7.7 Hz, 1H), 7.33 (d, *J* = 1.4 Hz, 1H), 7.23 (s, 1H), 7.14 (d, *J* = 7.5 Hz, 1H), 7.08 (s, 1H), 6.86 (d, *J* = 7.8 Hz, 1H), 5.33 (s, 1H), 4.66 (s, 1H), 4.45 (s, 1H), 3.78 (s, 1H), 3.66 (s, 1H), 2.70 (s, 1H), 2.29 (d, *J* = 7.8 Hz, 2H), 2.00 (s, 2H), 0.90 (s, 1H), 0.38 (d, *J* = 2.6 Hz, 1H), 0.21 (d, *J* = 22.6 Hz, 3H). **^{13}C NMR** (101 MHz, $CDCl_3$) δ 175.6, 166.9, 166.0, 140.4, 136.1, 132.1, 131.4, 129.0, 128.1, 127.9, 126.8, 126.1 (q, *J* = 6.1 Hz), 125.6, 122.9 (q, *J* = 11.1 Hz), 110.0, 63.9, 63.8, 60.9, 60.5, 53.5, 51.1, 45.7, 27.2, 23.9, 9.0, 3.4, 3.1. **HRMS** (ESI, m/z): $C_{27}H_{24}F_3N_3NaO_3$ $[M+Na]^+$, Calculated mass: 518.1662; found: 518.1669.

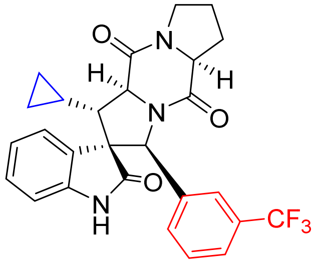

(3*R*,8*S*,9*S*,12*S*,18*S*)-8-cyclopropyl-18-[3-(trifluoromethyl)phenyl]-8,9,12,13,14,15-hexahydro-11*H*,17*H*,18*H*-spiro[bispyrrolo[1,2-a:1′,2′-d]pyrazine-3,3′-indoline]-2,11,17-trione (4f): White solid, 27.7 mg, 56% yield, R_f =0.34. (petroleum ether/ethyl acetate=1/1), m.p.: 178~179°C. **^{1}H NMR** (400 MHz, $CDCl_3$) δ 7.51 (d, *J* = 7.8 Hz, 1H), 7.44 – 7.28 (m, 2H), 7.26 – 7.20 (m, 1H), 7.20 – 7.03 (m, 4H), 4.69 – 4.61 (m, 1H), 4.43 (t, *J* = 7.4 Hz, 1H), 3.84 – 3.73 (m, 1H), 3.64 – 3.55 (m, 1H), 2.69 – 2.59 (m, 1H), 2.33 – 2.25 (m, 2H), 2.01 – 1.94 (m, 2H), 1.57 – 1.52 (m, 2H), 0.88 (s, 1H), 0.38 – 0.02 (m, 4H). **^{13}C NMR** (101 MHz, $CDCl_3$) δ 175.1, 174.0, 167.4, 166.1, 143.3, 140.3, 137.9, 130.6 (q, *J* = 32.3 Hz), 129.0, 128.9, 125.0 (q, *J* = 4.0 Hz), 124.0 (q, *J* = 273.7 Hz), 123.0 (q, *J* = 12.4 Hz), 110.6, 68.3, 64.2, 60.9, 52.0, 50.3, 45.7, 31.6, 27.2, 23.9, 14.1, 9.3, 3.5, 3.2. **HRMS** (ESI, m/z): $C_{27}H_{25}F_3N_3O_3$ $[M+H]^+$, Calculated mass: 496.1843; found: 496.1848.

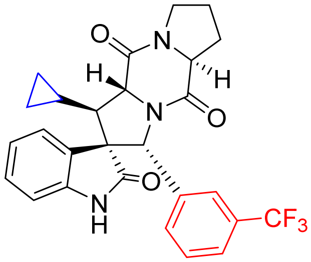

(3*S*,8*R*,9*R*,12*S*,18*R*)-8-cyclopropyl-18-[3-(trifluoromethyl)phenyl]-8,9,12,13,14,15-hexahydro-11*H*,17*H*,18*H*-spiro[bispyrrolo[1,2-a:1′,2′-d]pyrazine-3,3′-indoline]-2,11,17-trione (4f'): White solid, 21.3 mg, 43% yield, R_f =0.28. (petroleum ether/ethyl acetate=1/1), m.p.: 183~184°C. **^{1}H NMR** (400 MHz, $CDCl_3$) δ 8.51 (s, 1H), 7.57 (d, *J* = 7.6 Hz, 1H), 7.47 (t, *J* = 7.7 Hz, 1H), 7.36 (t, *J* = 7.1 Hz, 1H), 7.28 (d, *J* = 7.5 Hz, 1H), 7.21 – 7.12 (m, 3H), 6.92 (d, *J* = 7.8 Hz, 1H), 4.71 (d, *J* = 9.4 Hz, 1H), 4.49 (t, *J* = 7.5 Hz, 1H), 3.89 – 3.80 (m, 1H), 3.71 – 3.61 (m, 1H), 2.68 (t, *J* = 9.5 Hz, 1H), 2.35 (d, *J* = 6.5 Hz,

2H), 2.04 (s, 2H), 1.32 (s, 1H), 0.94 (s, 1H), 0.44 – 0.16 (m, 4H). **^{13}C NMR** (101 MHz, $CDCl_3$) δ 175.3, 164.8, 164.6, 144.1, 135.4, 131.2 (q, J = 30.3 Hz), 130.3, 128.9, 128.1, 128.0, 125.8 (q, J = 5.8 Hz), 124.1 (q, J = 273.7 Hz), 123.3 (q, J = 4.0 Hz), 110.0, 63.6, 62.6, 59.6, 55.6, 51.8, 44.9, 31.6, 25.8, 23.8, 14.2, 8.9, 2.3, 1.1. **HRMS** (ESI, m/z): $C_{27}H_{24}F_3N_3NaO_3$ $[M+Na]^+$, Calculated mass: 518.1662; found: 518.1663.

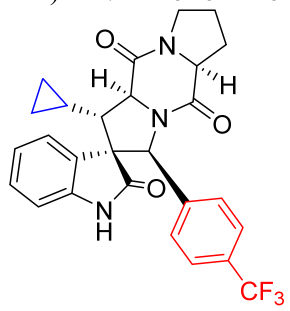

(3*R*,8*S*,9*S*,12*S*,18*S*)-8-cyclopropyl-18-[4-(trifluoromethyl)phenyl]-8,9,12,13,14,15-hexahydro-11*H*,17*H*,18*H*-spiro[bispyrrolo[1,2-a:1′,2′-d]pyrazine-3,3′-indoline]-2,11,17-trione (4g): White solid, 28.7 mg, 58% yield, R_f =0.30. (petroleum ether/ethyl acetate=1/1), m.p.: 203~204°C. **^{1}H NMR** (400 MHz, $CDCl_3$) δ 7.60 – 7.56 (m, 2H), 7.43 (t, J = 6.3 Hz, 1H), 7.25 – 7.17 (m, 2H), 7.11 – 7.03 (m, 3H), 5.27 (s, 1H), 4.73 (d, J = 8.9 Hz, 1H), 4.45 (d, J = 31.9 Hz, 2H), 3.84 (s, 1H), 3.67 (s, 1H), 2.77 (s, 1H), 2.35 (s, 2H), 1.48 (s, 2H), 0.96 (s, 1H), 0.48 – 0.17 (m, 4H). **^{13}C NMR** (101 MHz, $CDCl_3$) δ 174.1, 167.4, 165.9, 143.1, 140.8, 130.6 (q, J = 42.4 Hz), 130.1, 129.4, 129.1, 126.9, 125.5 (q, J = 14.1 Hz), 124.2 (q, J = 282.8 Hz), 123.1 (q, J = 4.0 Hz), 110.6, 68.4, 64.2, 60.9, 60.8, 52.0, 50.4, 45.6, 27.2, 25.4, 23.9, 9.4, 3.6, 3.4. **HRMS** (ESI, m/z): $C_{27}H_{25}F_3N_3O_3$ $[M+H]^+$, Calculated mass: 496.1843; found: 496.1848.

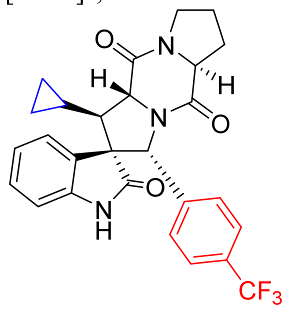

(3*S*,8*R*,9*R*,12*S*,18*R*)-8-cyclopropyl-18-[4-(trifluoromethyl)phenyl]-8,9,12,13,14,15-hexahydro-11*H*,17*H*,18*H*-spiro[bispyrrolo[1,2-a:1′,2′-d]pyrazine-3,3′-indoline]-2,11,17-trione (4g'): White solid, 21.8 mg, 44% yield, R_f =0.26. (petroleum ether/ethyl acetate=1/1), m.p.: 188~189°C. **^{1}H NMR** (400 MHz, $CDCl_3$) δ 7.58 – 7.53 (m, 2H), 7.39 (t, J = 7.8 Hz, 1H), 7.29 – 7.25 (m, 1H), 7.18 – 7.14 (m, 2H), 7.08 – 7.02 (m, 2H), 5.24 (s, 1H), 4.70 (d, J = 10.1 Hz, 1H), 4.45 (d, J = 7.6 Hz, 1H), 4.33 (d, J = 7.4 Hz, 2H), 3.83 – 3.76 (m, 1H), 3.67 – 3.60 (m, 1H), 2.73 (q, J = 9.5 Hz, 1H), 2.35 – 2.28 (m, 2H), 1.46 – 1.41 (m, 2H), 0.43 – 0.12 (m, 4H). **^{13}C NMR** (101 MHz, $CDCl_3$) δ 174.0, 167.4, 166.0, 143.4, 140.9, 130.9, 130.4, 130.0, 129.0, 127.0, 125.4 (q, J = 3.0 Hz), 124.1 (q, J = 277.8 Hz), 122.9 (q, J = 4.0 Hz), 110.5, 68.5, 64.3, 63.3, 60.9, 52.1, 50.3, 45.7, 27.2, 25.7, 23.9, 9.4, 3.6, 3.4. **HRMS** (ESI, m/z): $C_{27}H_{24}F_3N_3NaO_3$ $[M+Na]^+$, Calculated mass: 518.1662; found: 518.1671.

7. ^{1}H NMR and ^{13}C NMR Spectra of compounds

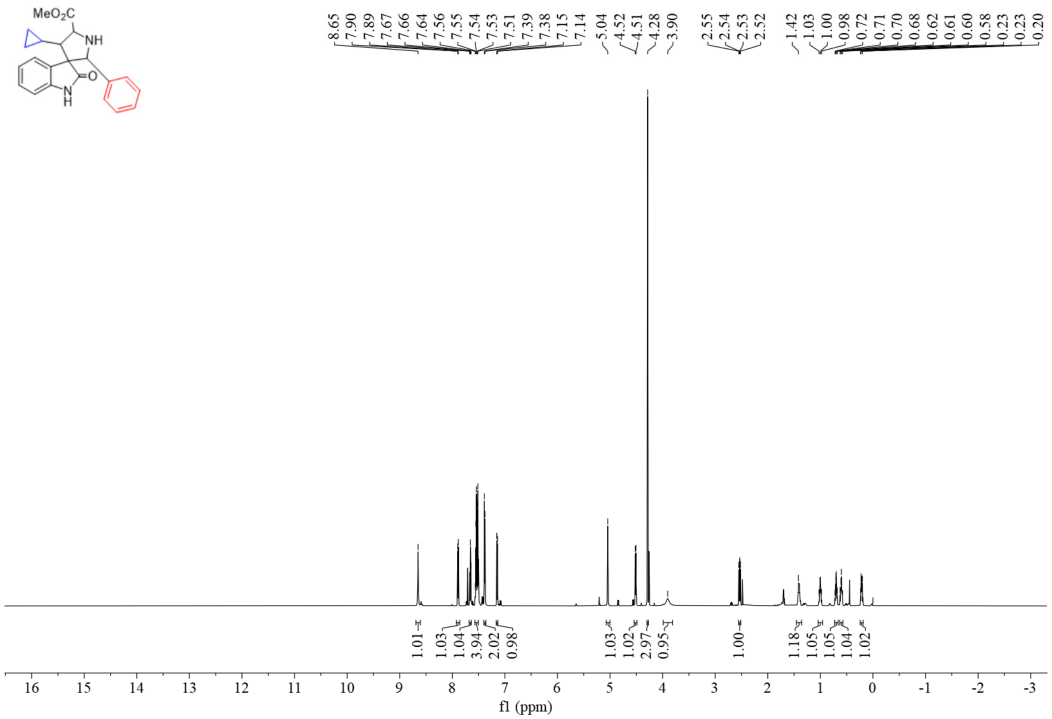

Figure S1. ^{1}H NMR spectrum of compound **3a**

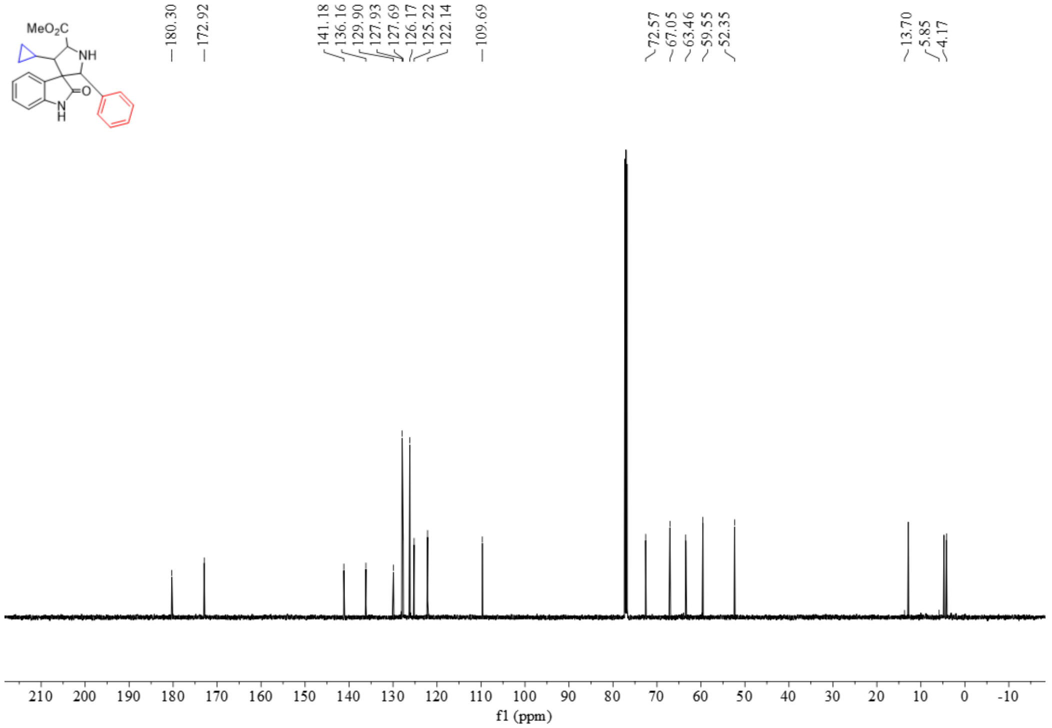

Figure S2. ^{13}C NMR spectrum of compound **3a**

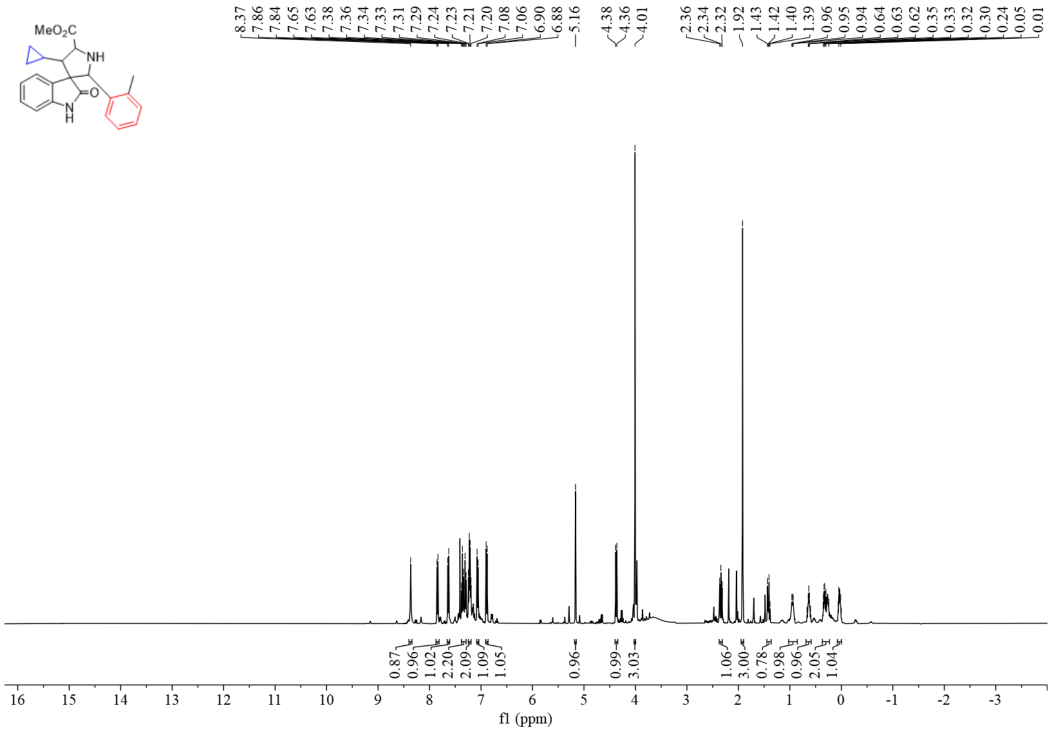

Figure S3. ^{1}H NMR spectrum of compound **3b**

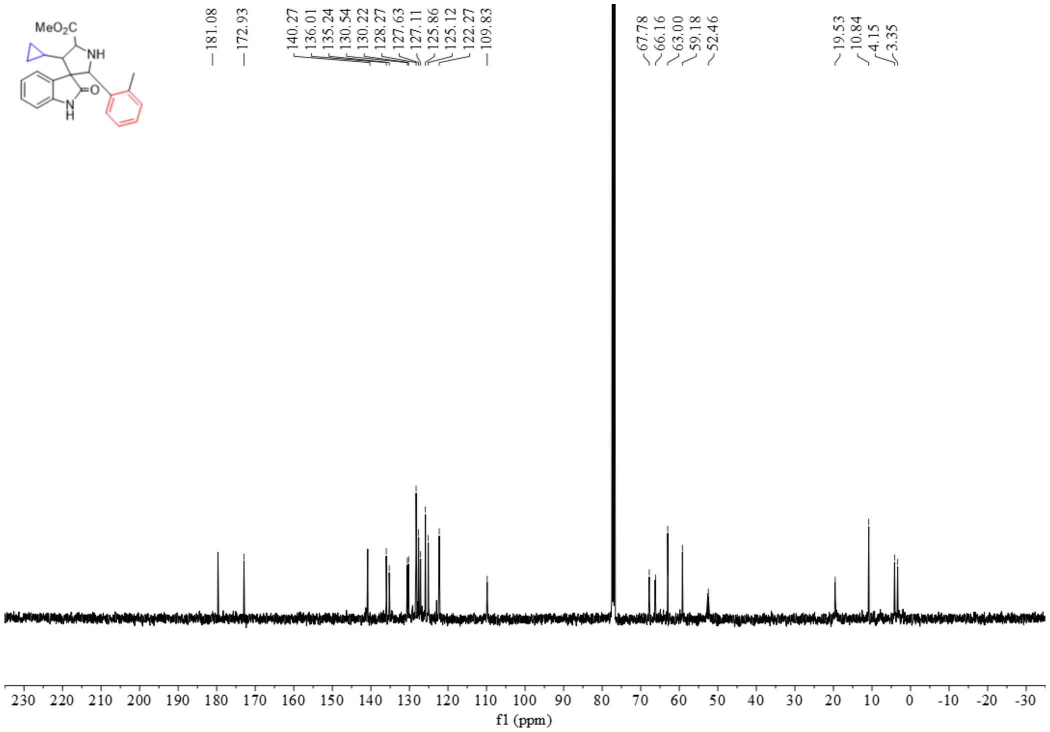

Figure S4. ^{13}C NMR spectrum of compound **3b**

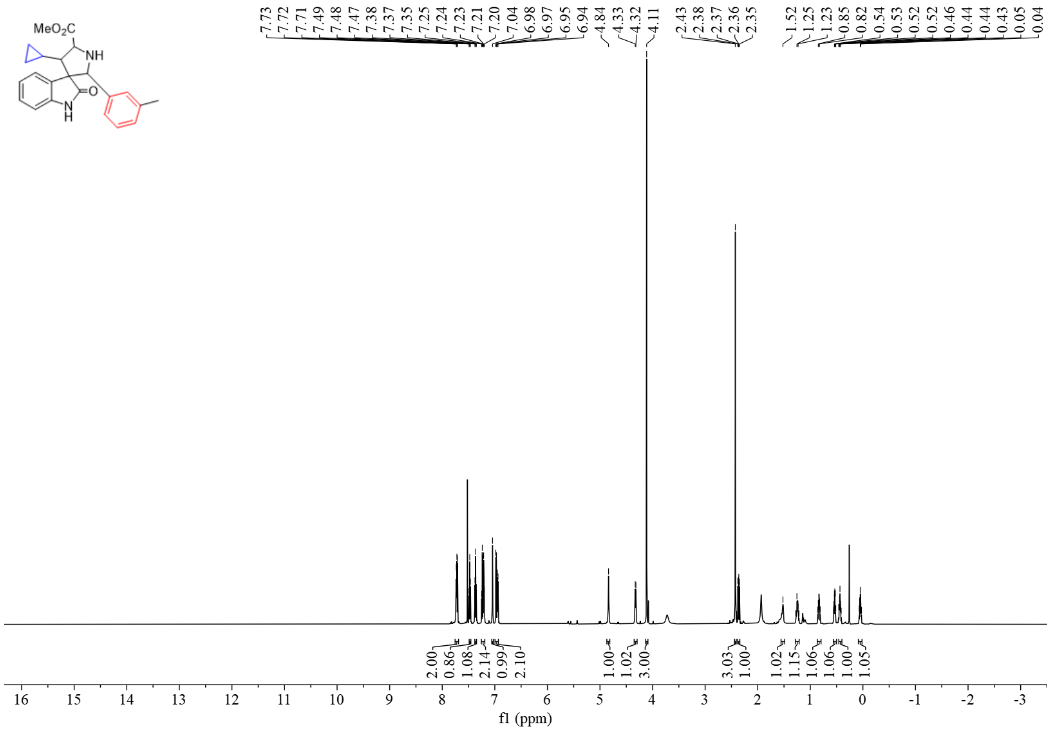

Figure S5. ^{1}H NMR spectrum of compound **3c**

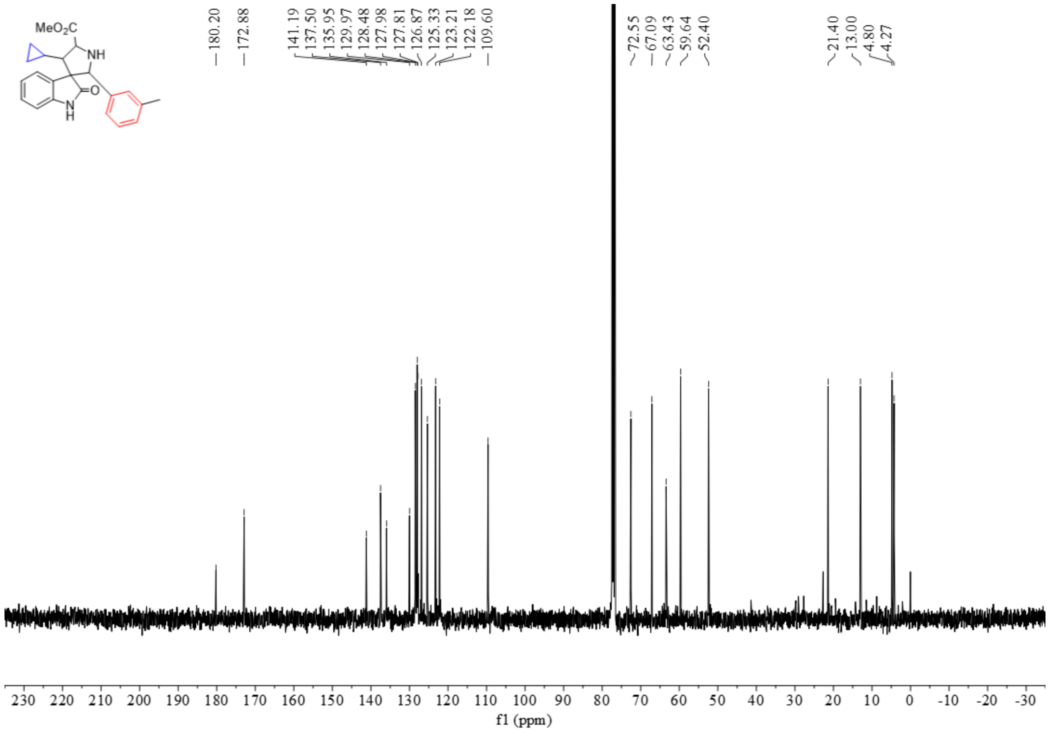

Figure S6. ^{13}C NMR spectrum of compound **3c**

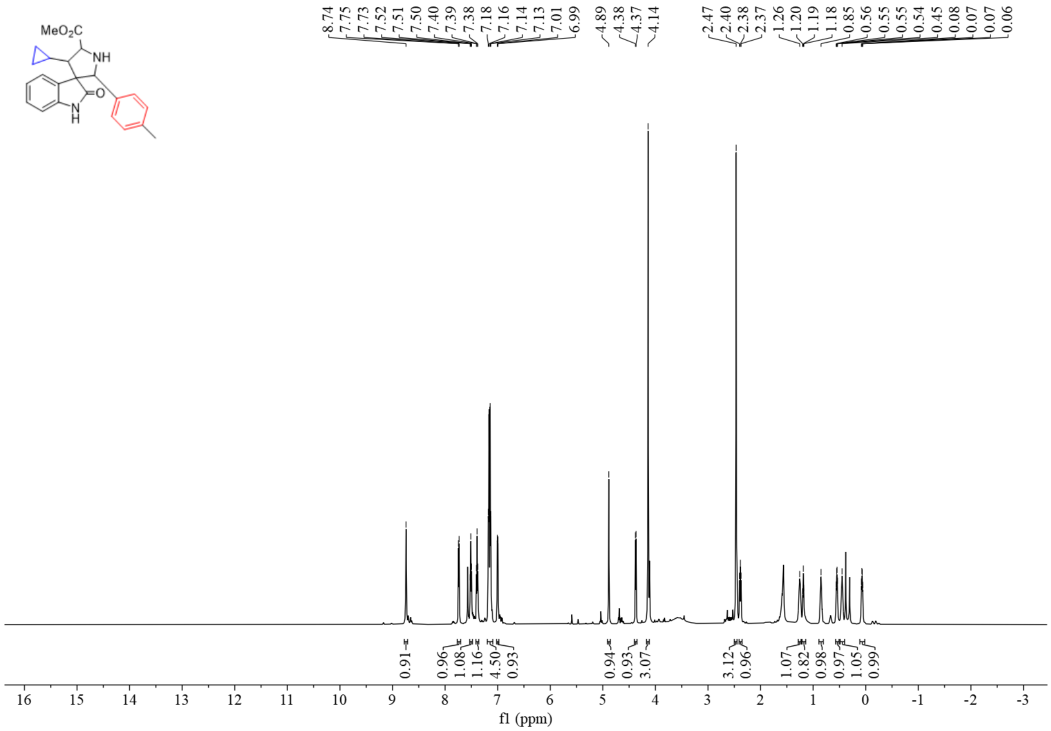

Figure S7. ^{1}H NMR spectrum of compound **3d**

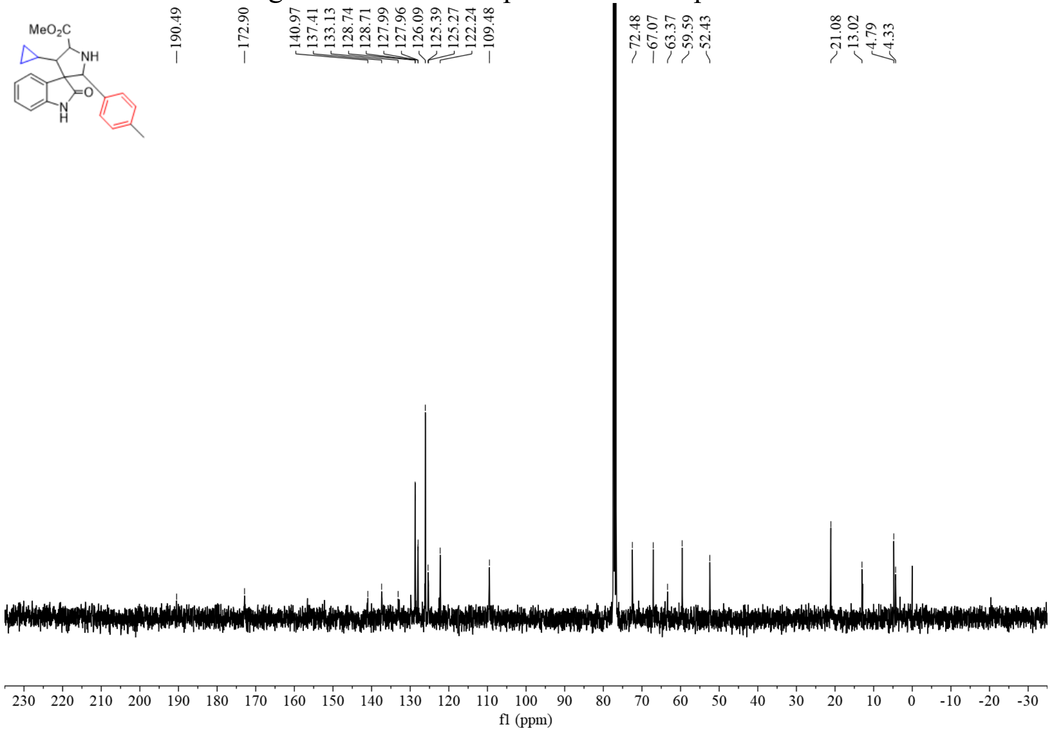

Figure S8. ^{13}C NMR spectrum of compound **3d**

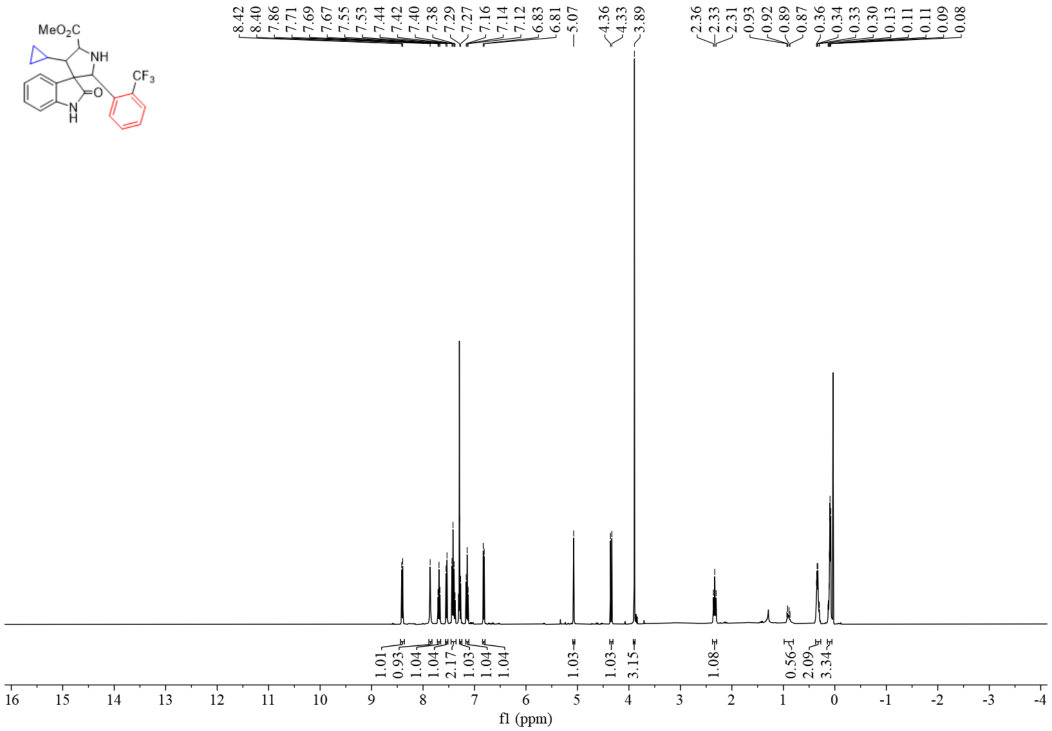

Figure S9. ^{1}H NMR spectrum of compound **3e**

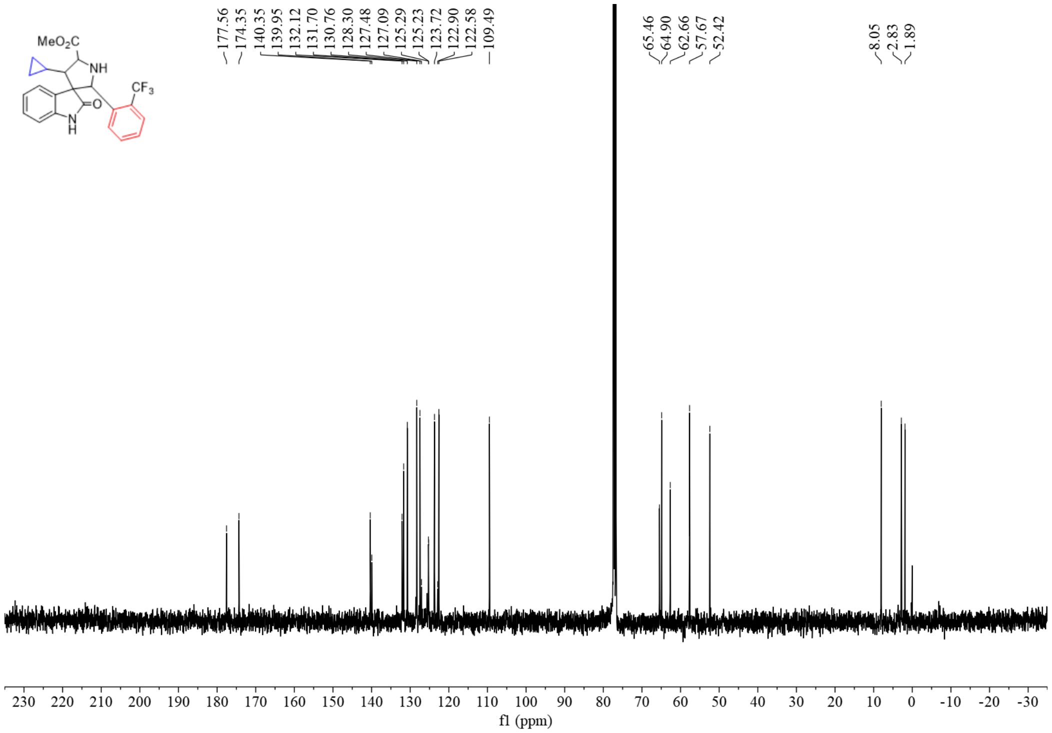

Figure S10. ^{13}C NMR spectrum of compound **3e**

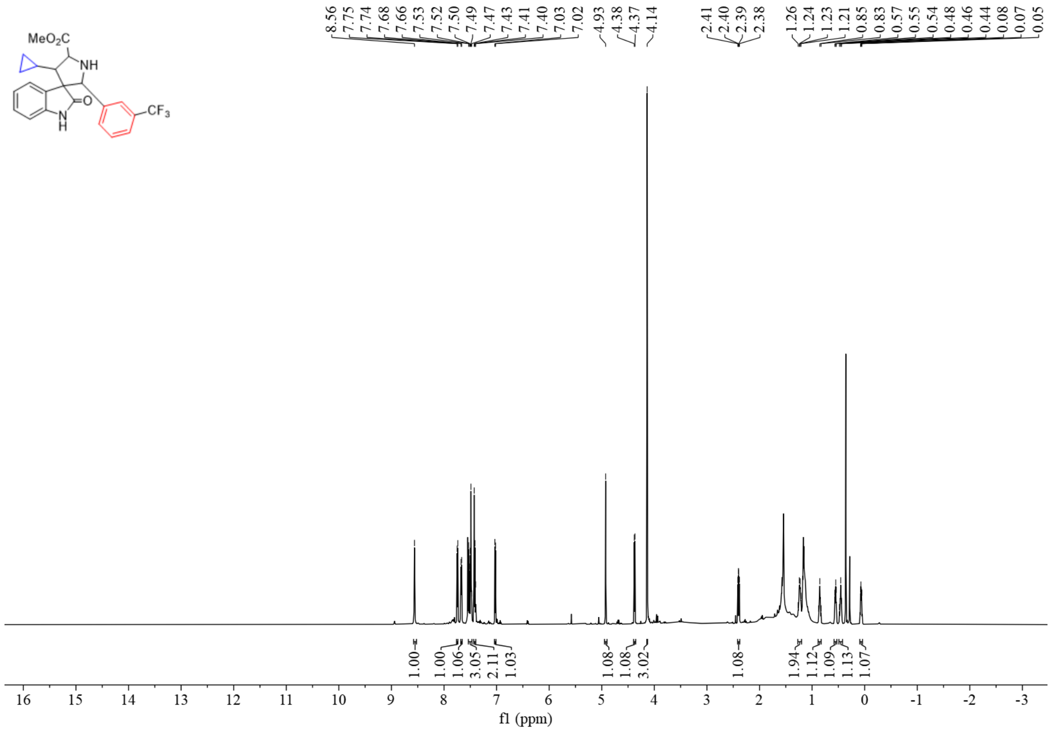

Figure S11. ^{1}H NMR spectrum of compound **3f**

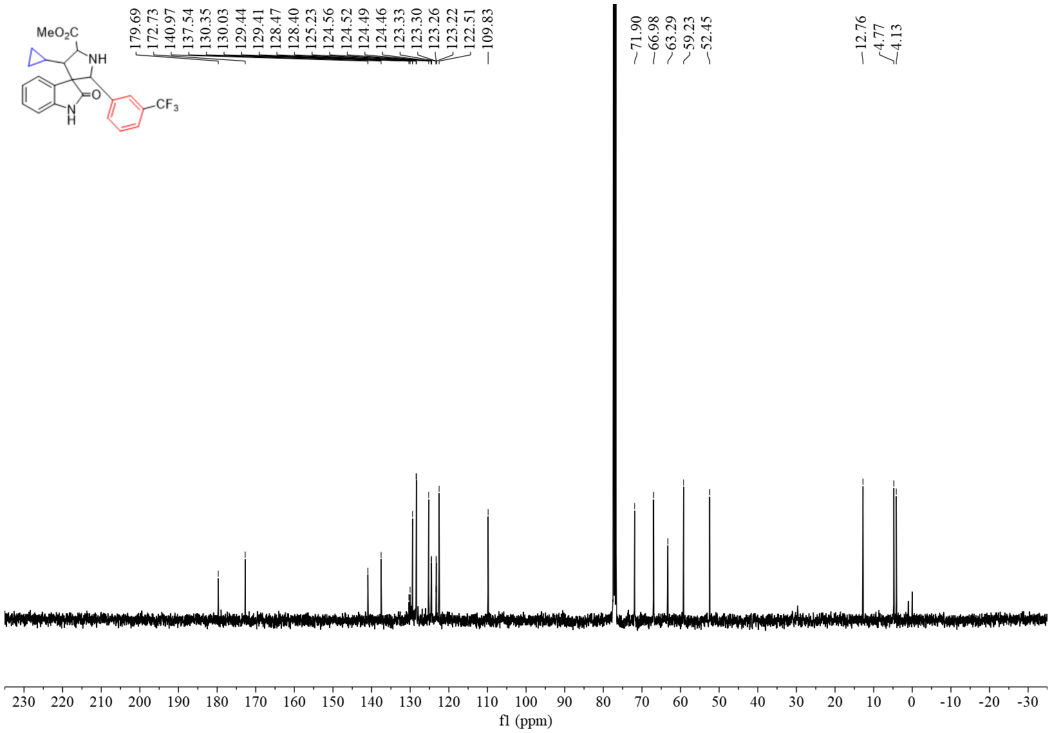

Figure S12. ^{13}C NMR spectrum of compound **3f**

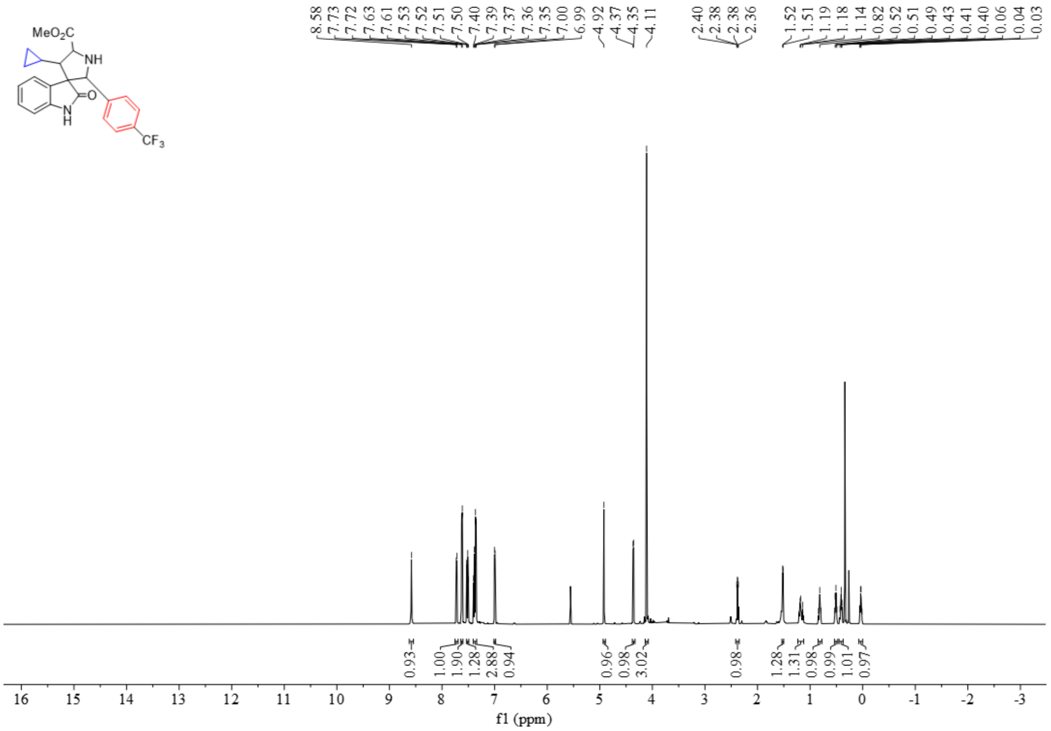

Figure S13. ^{1}H NMR spectrum of compound **3g**

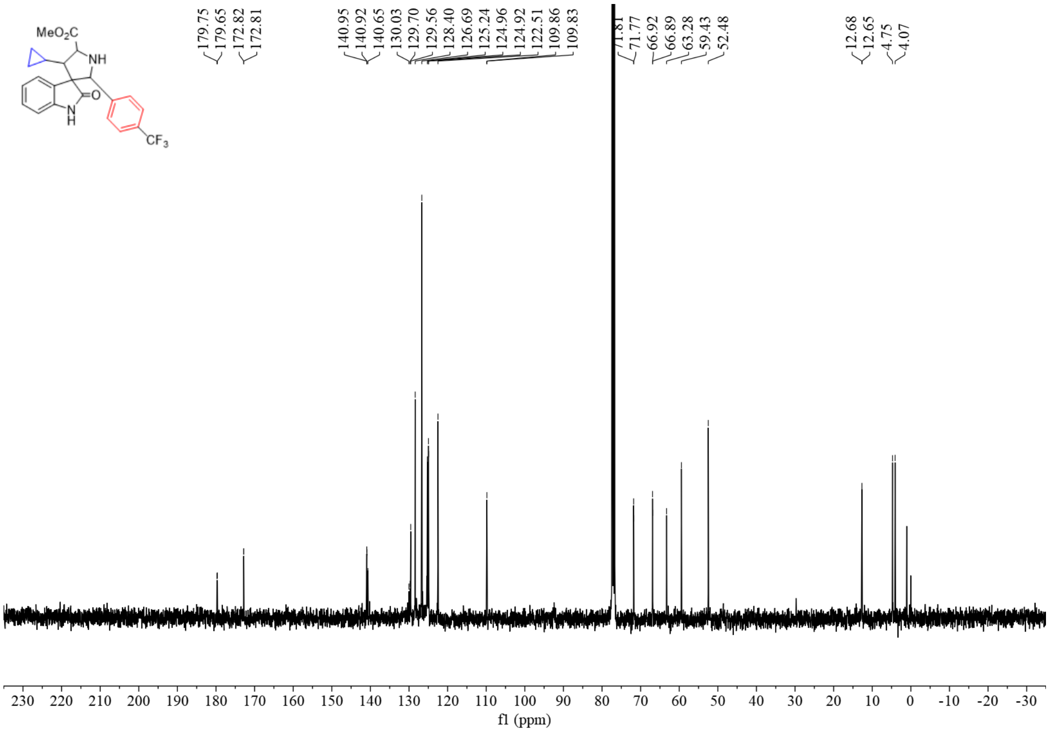

Figure S14. ^{13}C NMR spectrum of compound **3g**

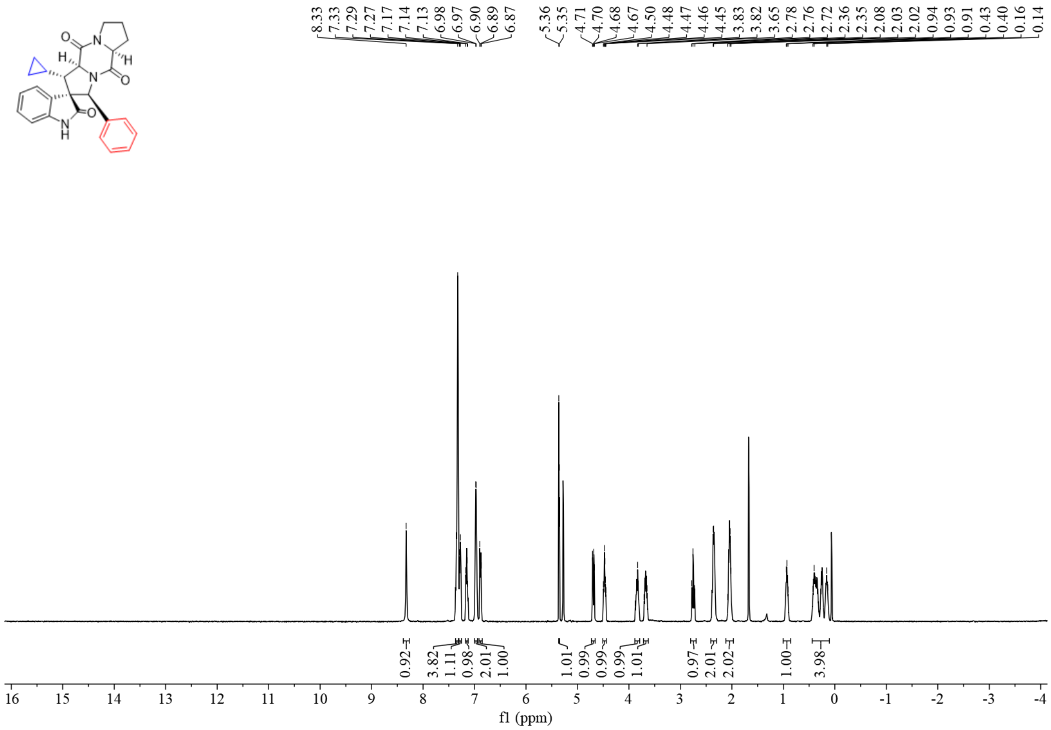

Figure S15. ^{1}H NMR spectrum of compound **4a**

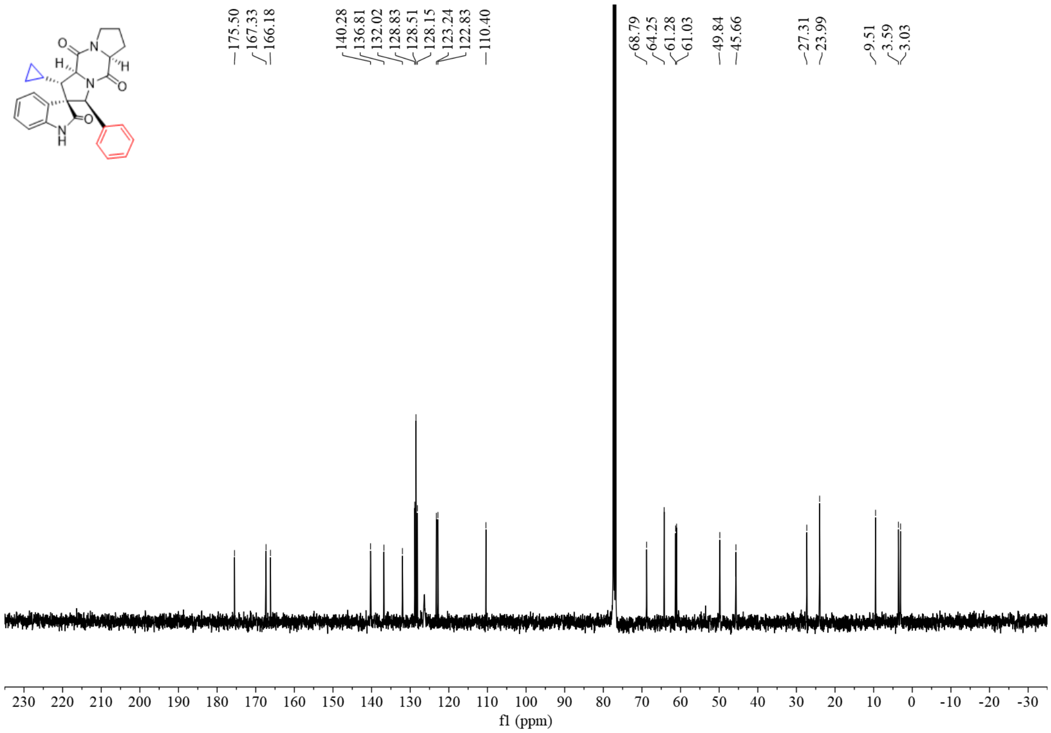

Figure S16. ^{13}C NMR spectrum of compound **4a**

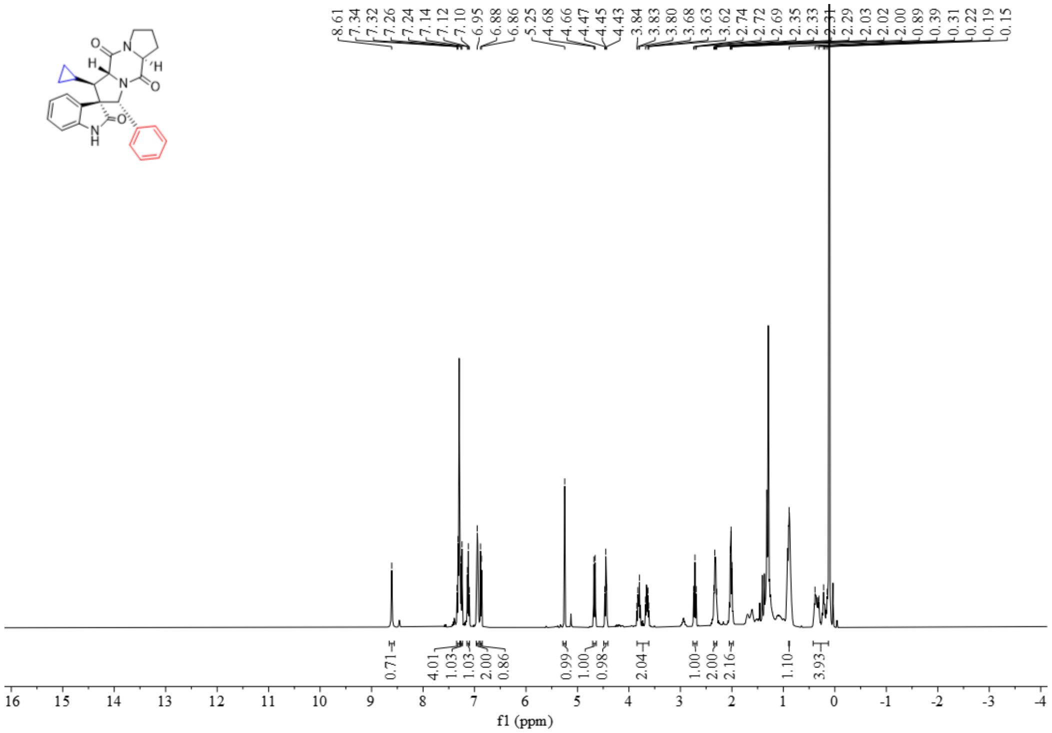

Figure S17. ^{1}H NMR spectrum of compound **4a'**

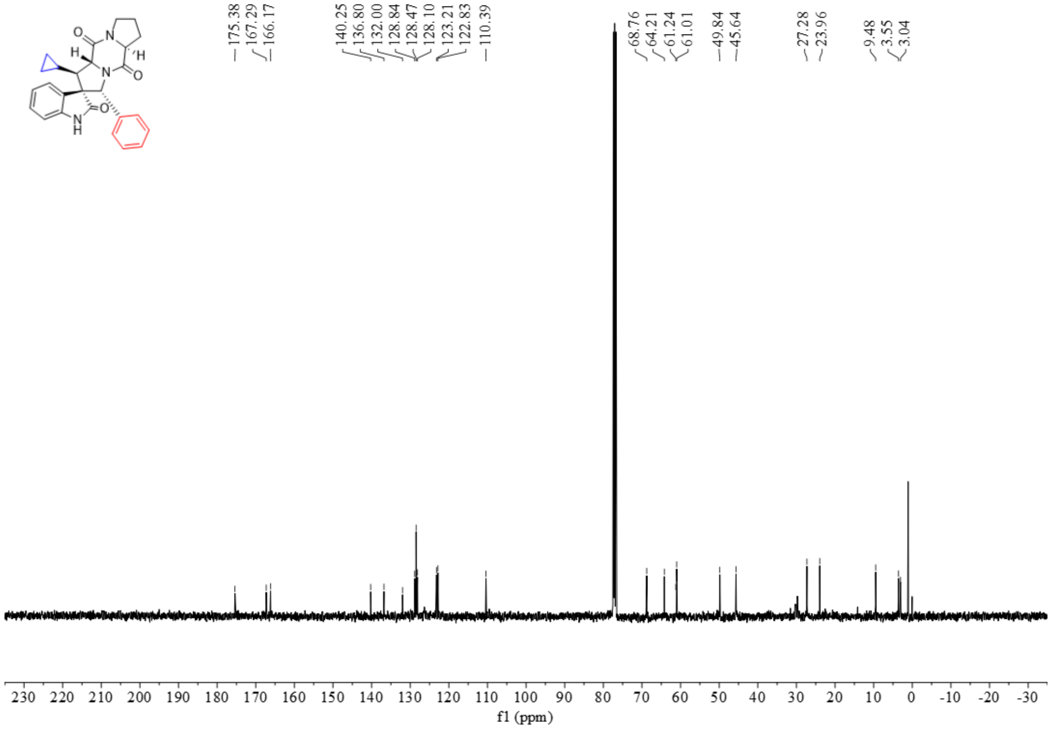

Figure S18. ^{13}C NMR spectrum of compound **4a'**

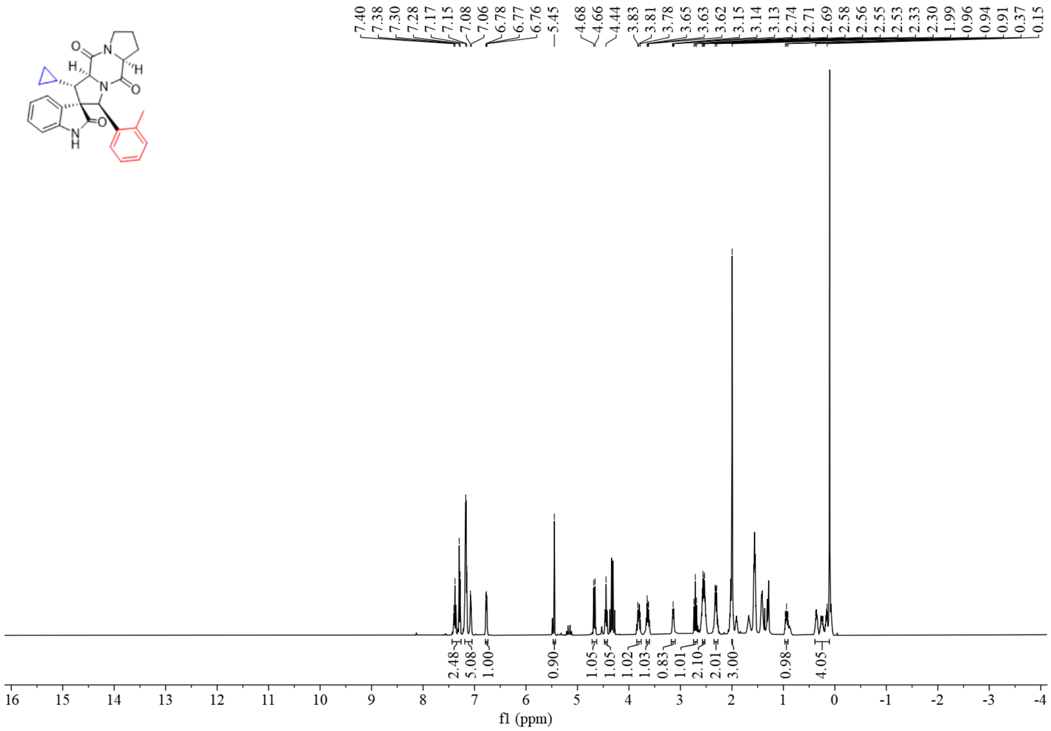

Figure S19. ^{1}H NMR spectrum of compound **4b**

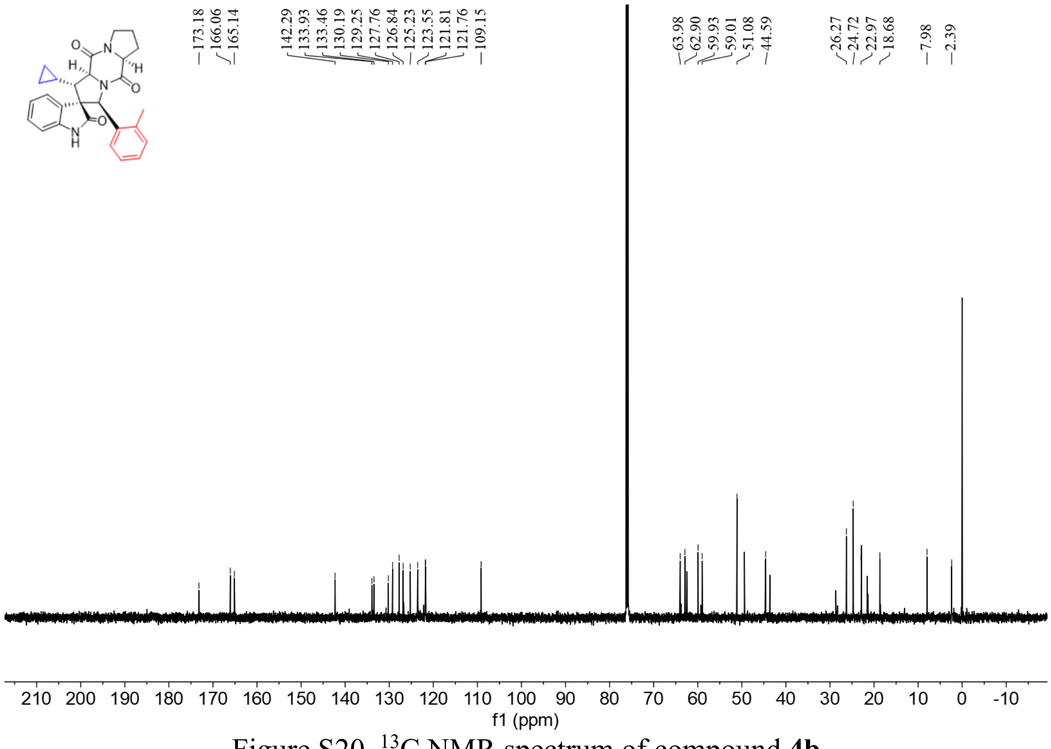

Figure S20. ^{13}C NMR spectrum of compound **4b**

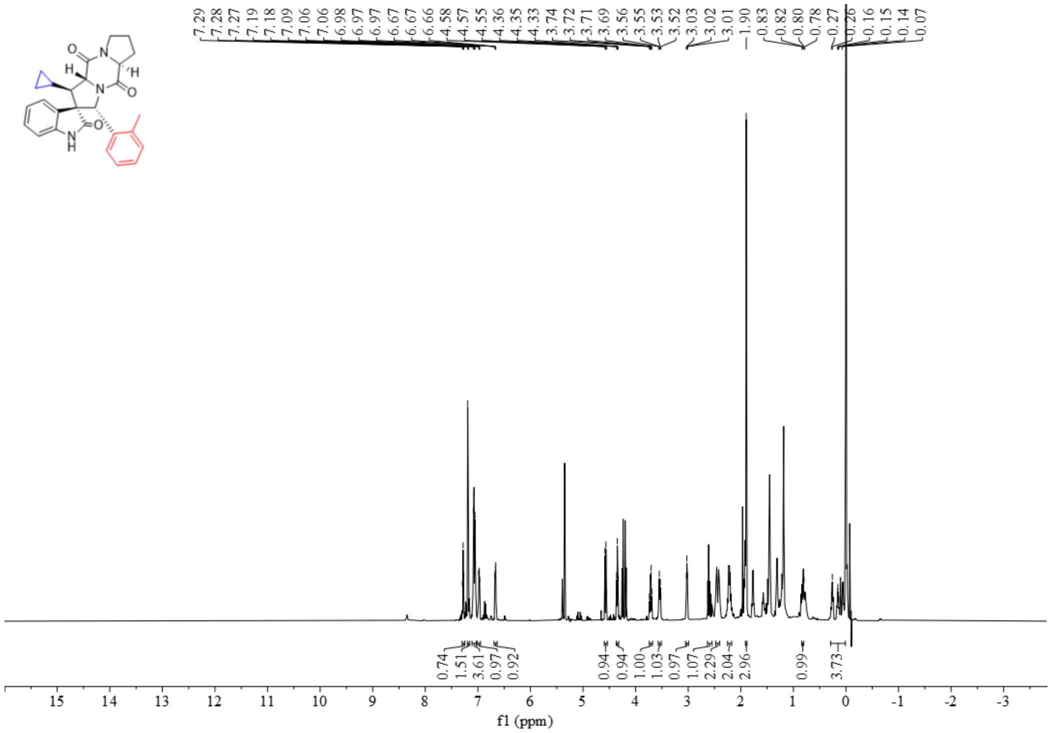

Figure S21. ^{1}H NMR spectrum of compound **4b'**

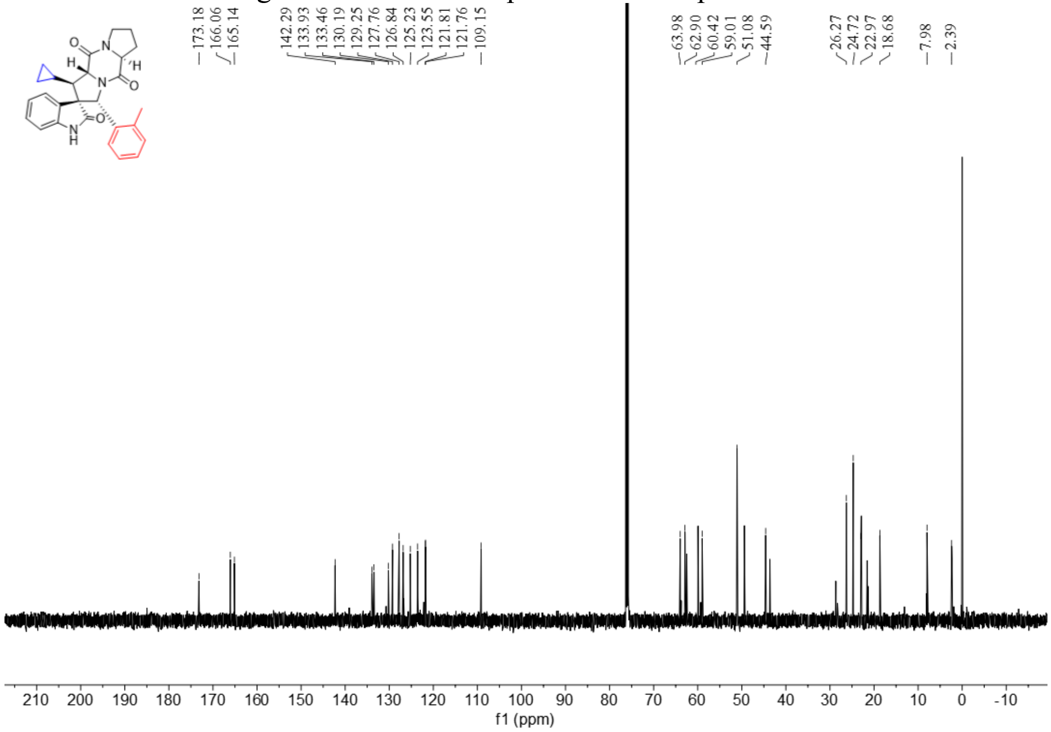

Figure S22. ^{13}C NMR spectrum of compound **4b'**

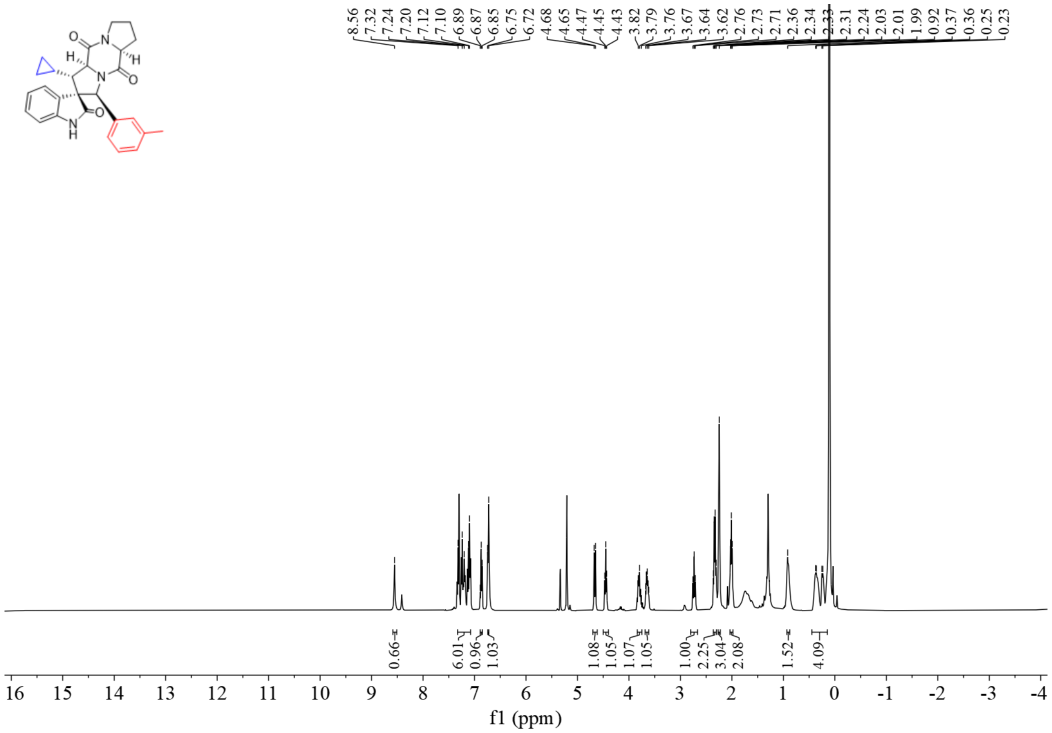

Figure S23. ^{1}H NMR spectrum of compound **4c**

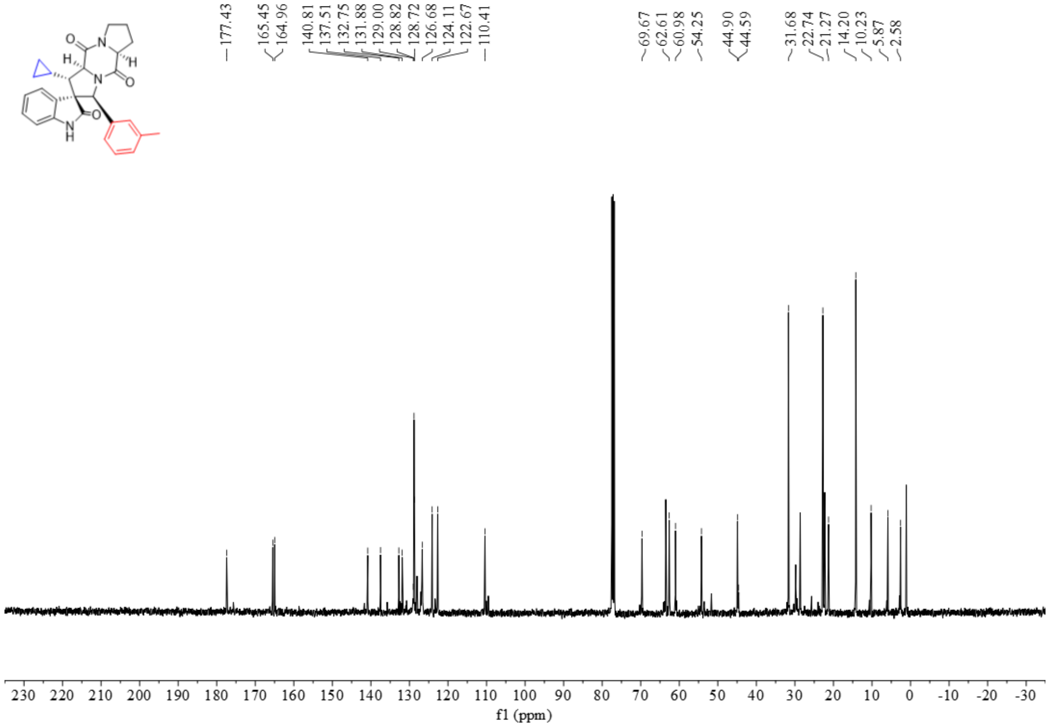

Figure S24. ^{13}C NMR spectrum of compound **4c**

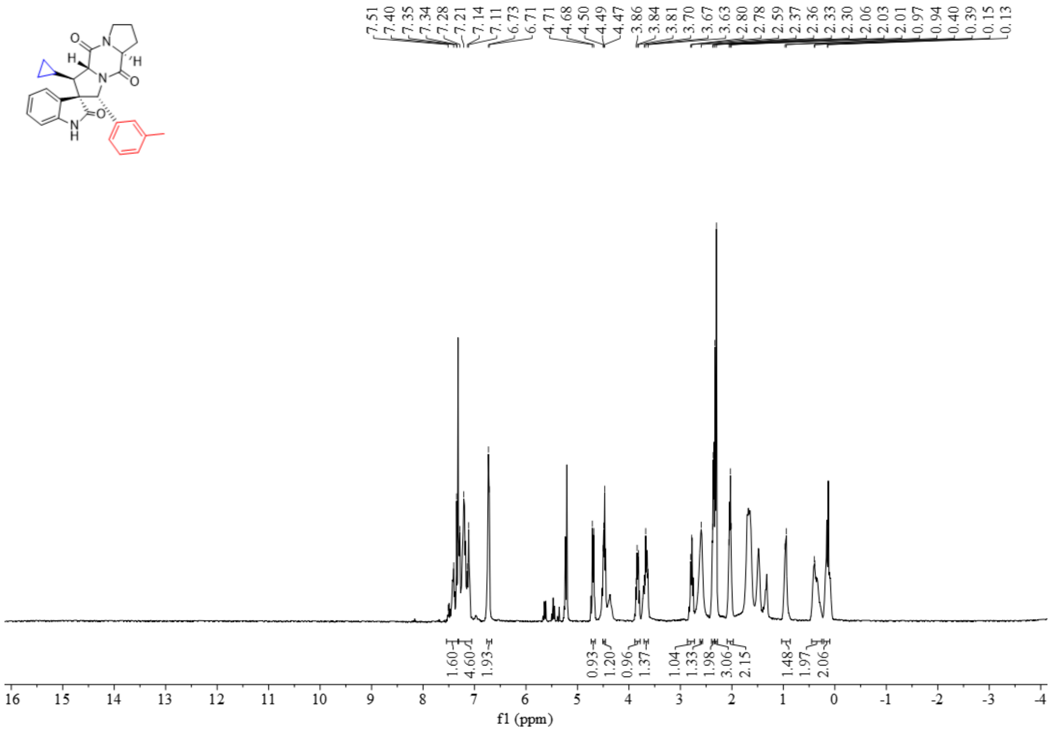

Figure S25. ^{1}H NMR spectrum of compound **4c'**

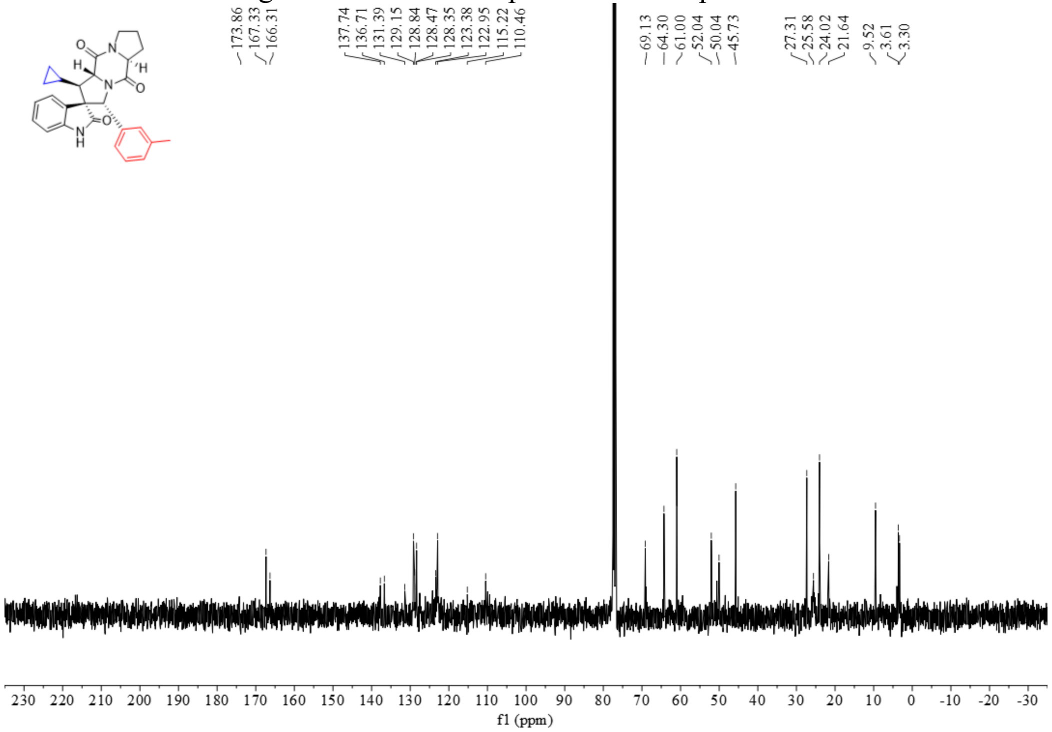

Figure S26. ^{13}C NMR spectrum of compound **4c'**

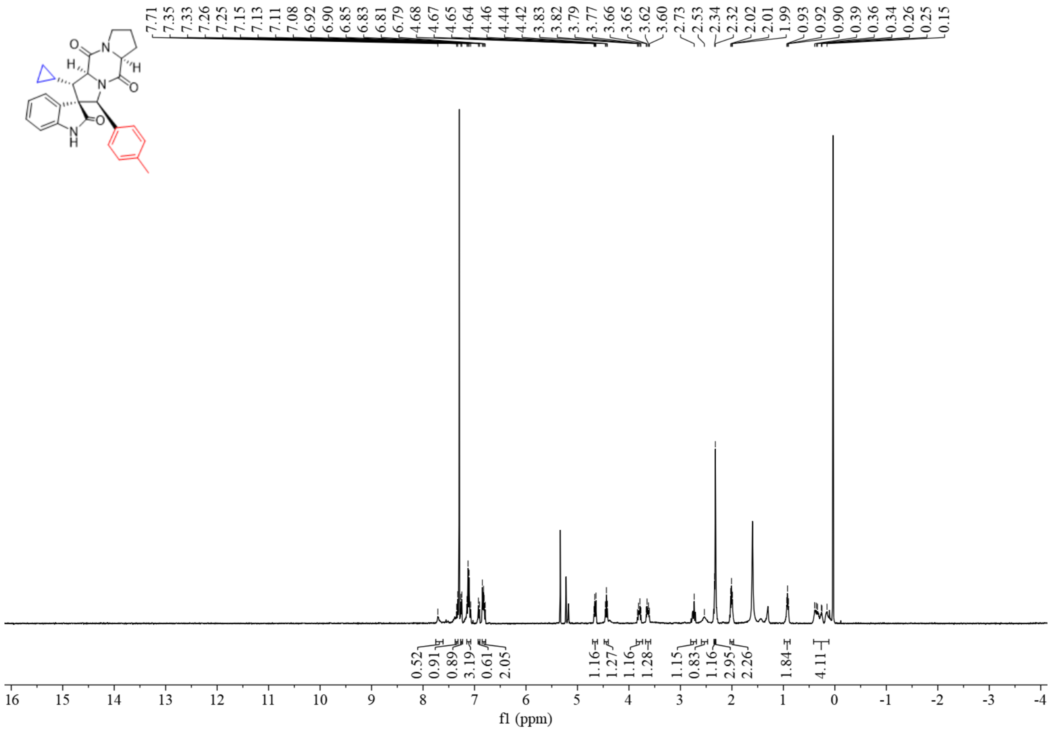

Figure S27. ^{1}H NMR spectrum of compound **4d**

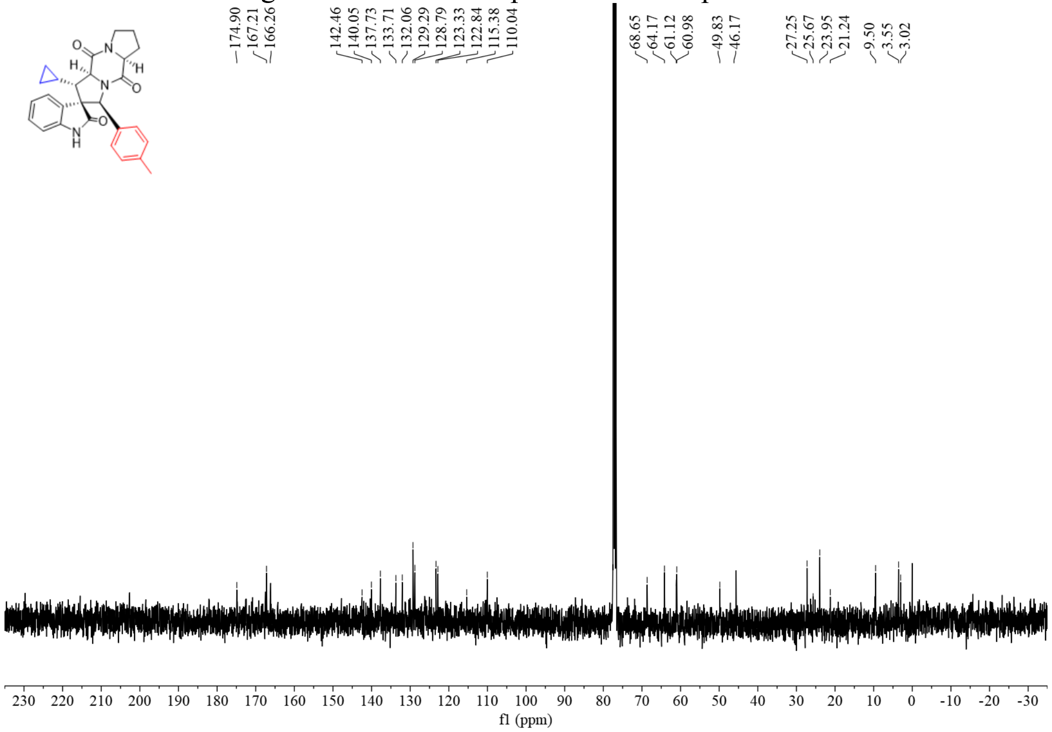

Figure S28. ^{13}C NMR spectrum of compound **4d**

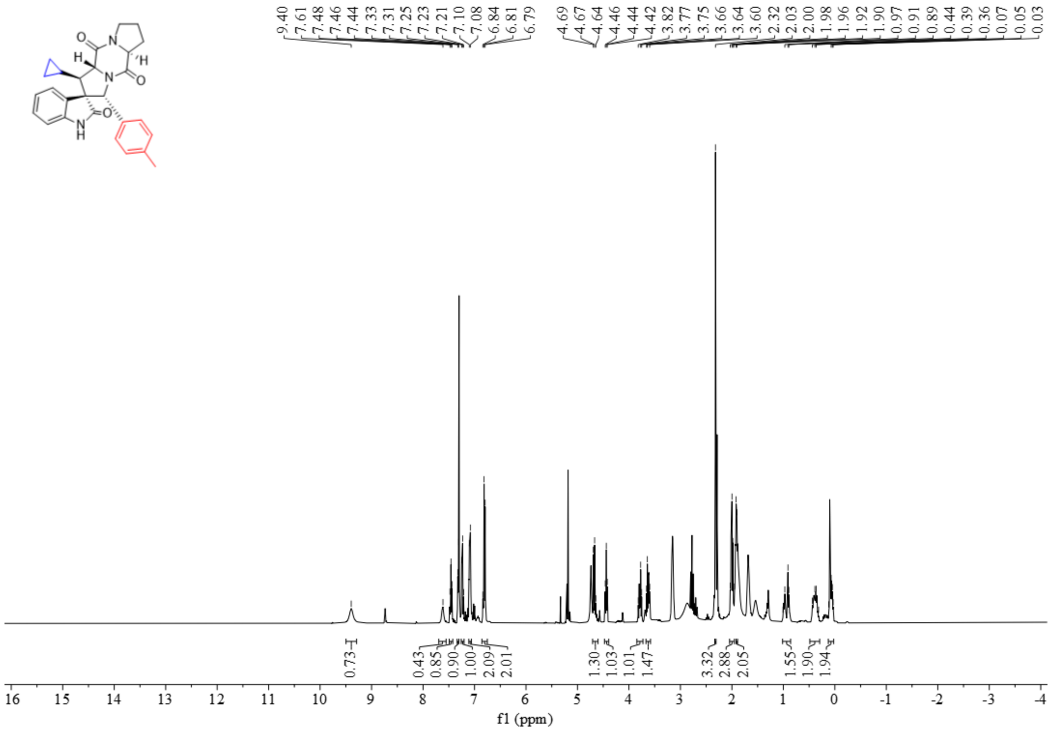

Figure S29. ^{1}H NMR spectrum of compound **4d'**

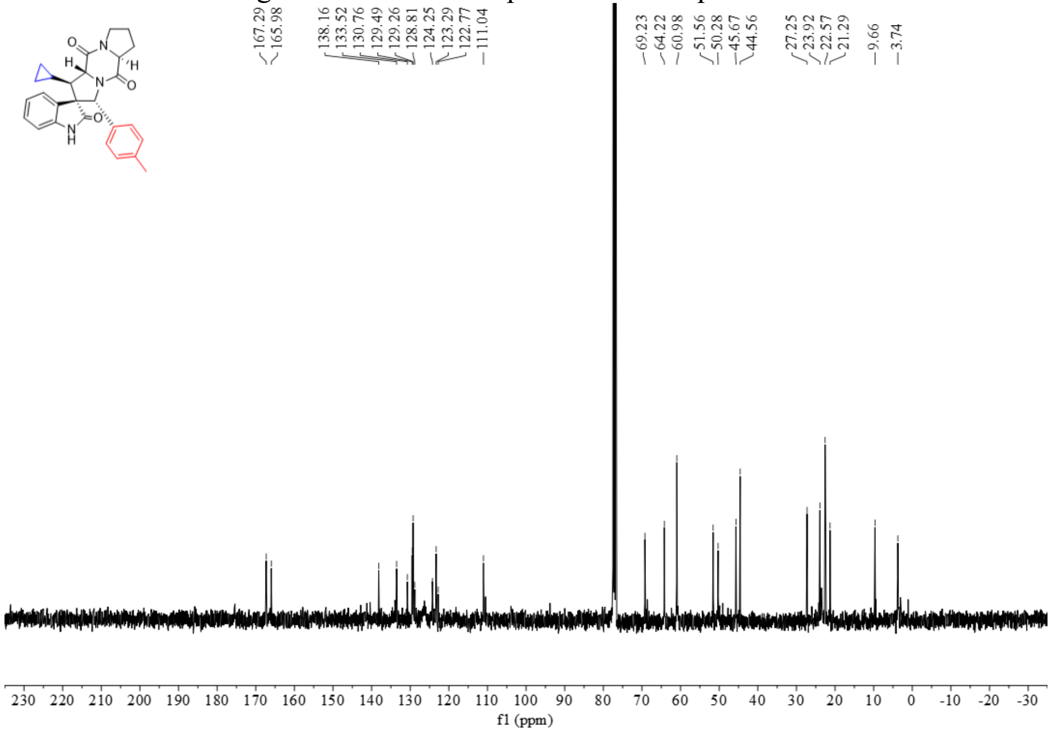

Figure S30. ^{13}C NMR spectrum of compound **4d'**

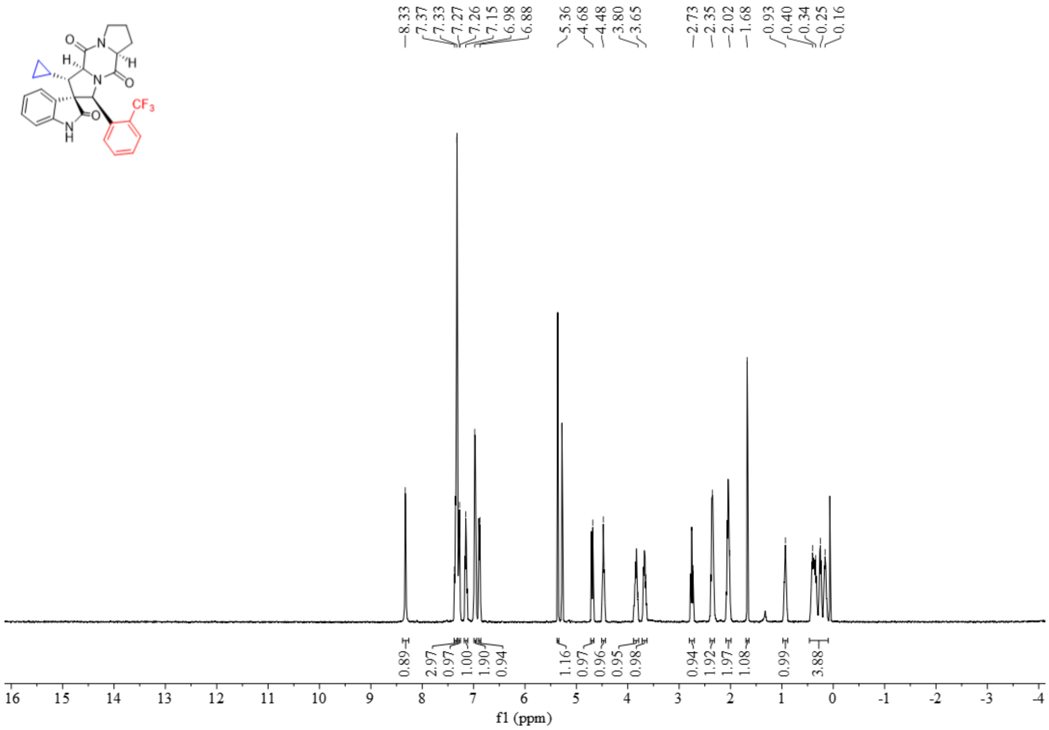

Figure S31. ^{1}H NMR spectrum of compound **4e**

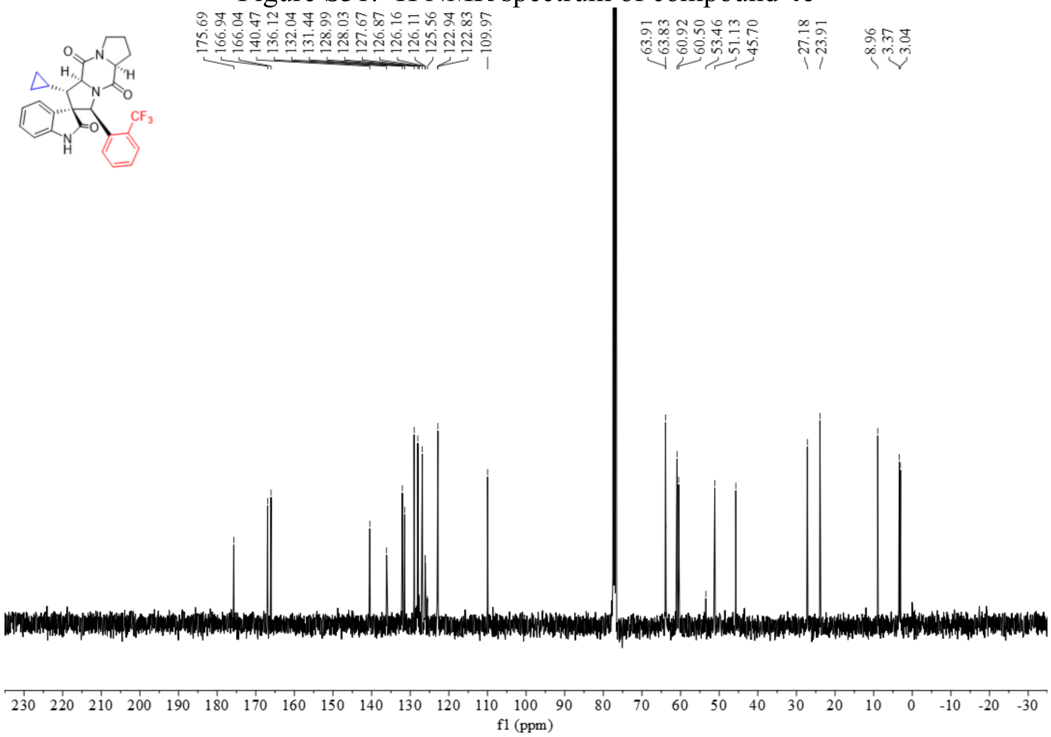

Figure S32. ^{13}C NMR spectrum of compound **4e**

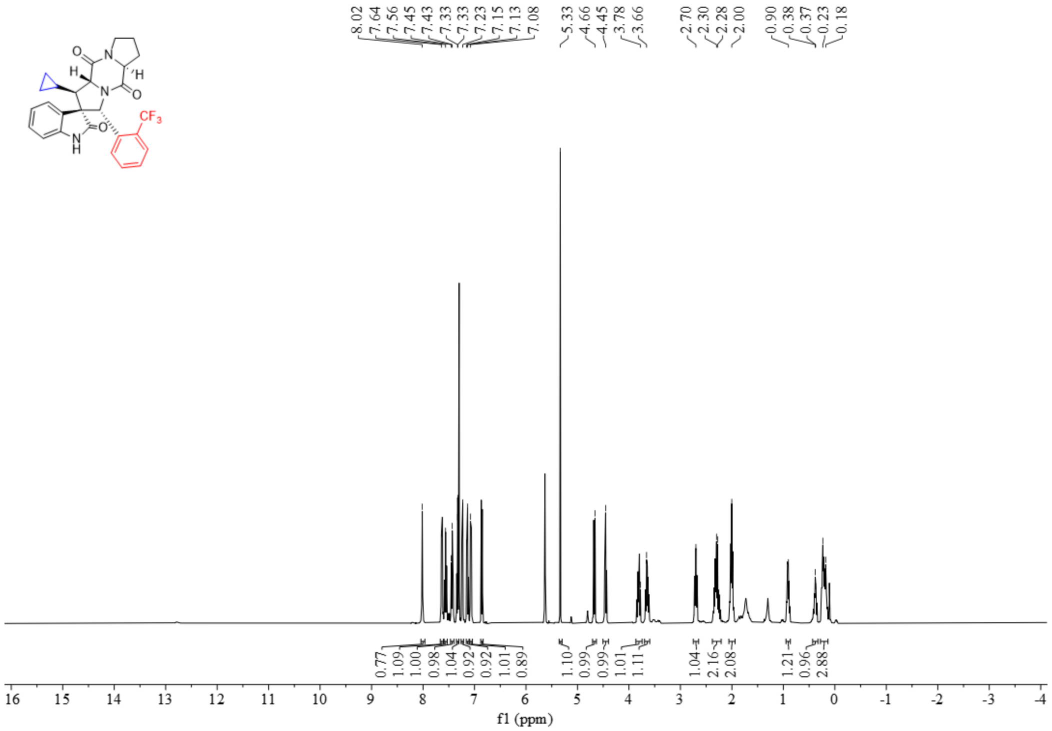

Figure S33. ^{1}H NMR spectrum of compound **4e'**

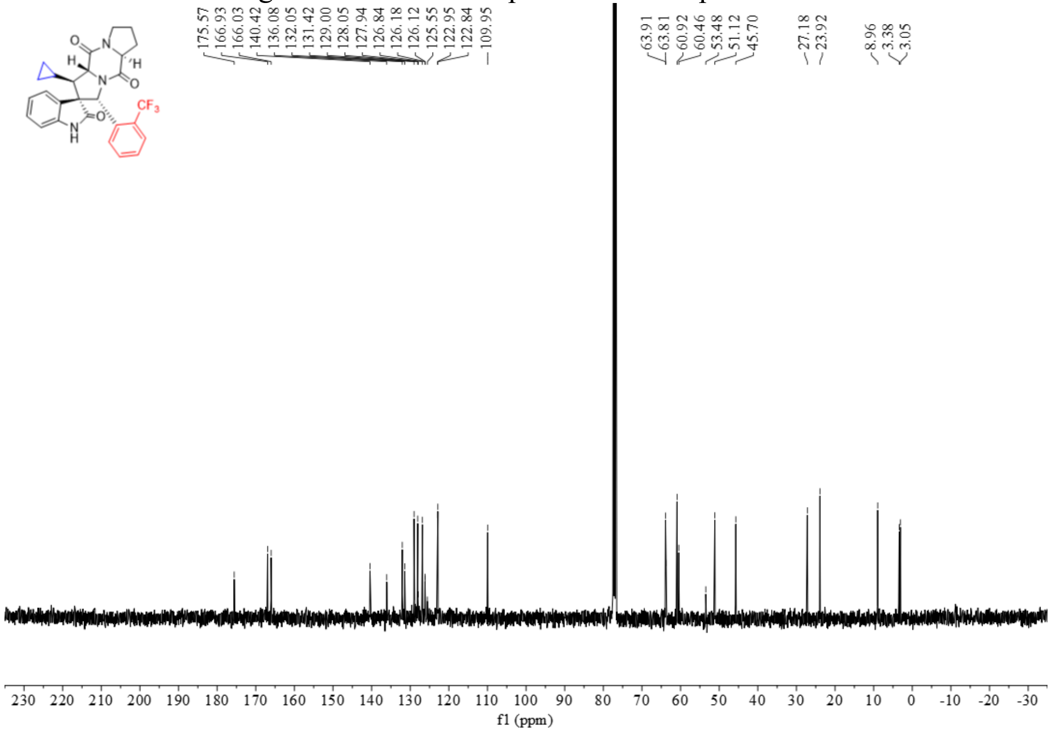

Figure S34. ^{13}C NMR spectrum of compound **4e'**

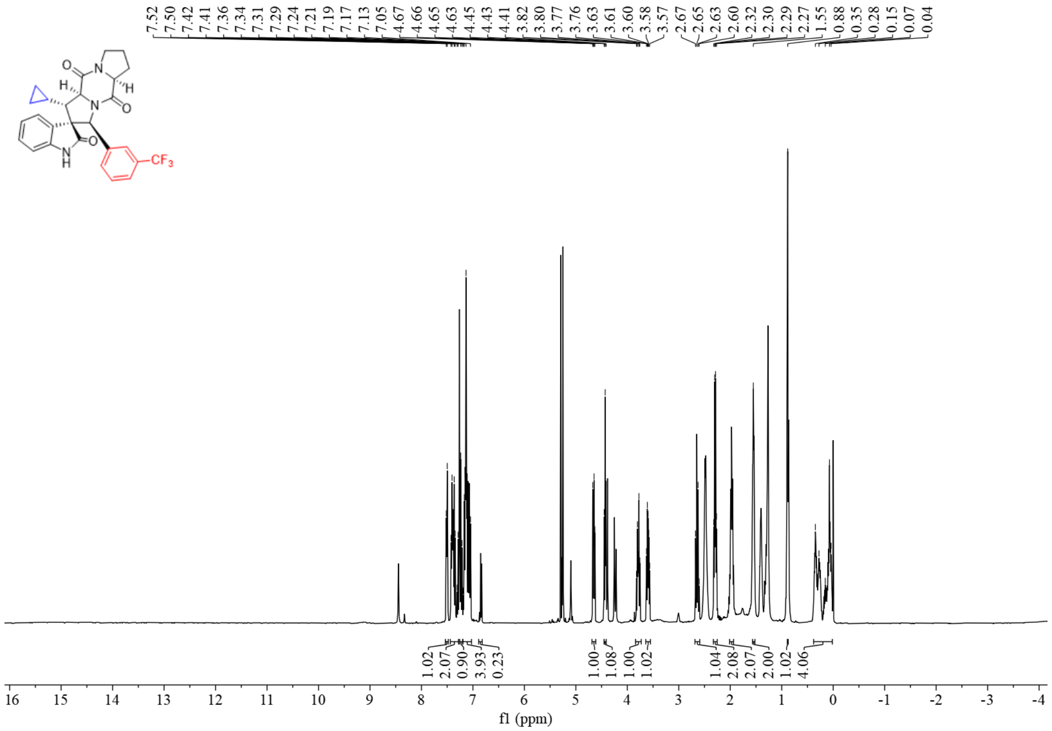

Figure S35. ^{1}H NMR spectrum of compound **4f**

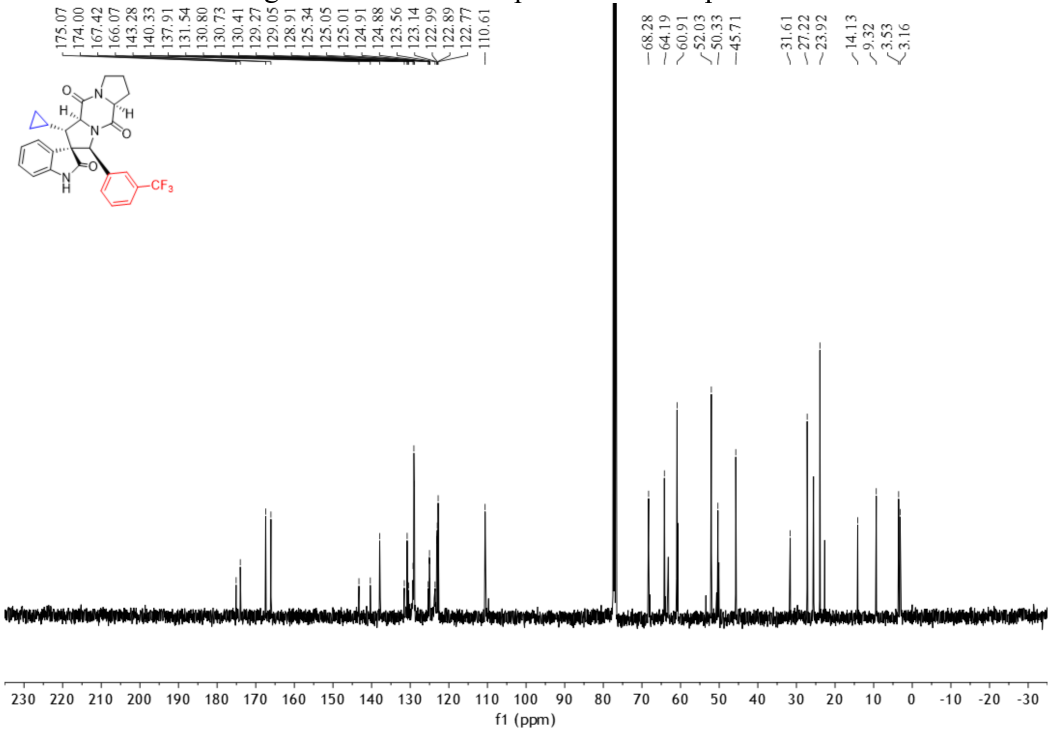

Figure S36. ^{13}C NMR spectrum of compound **4f**

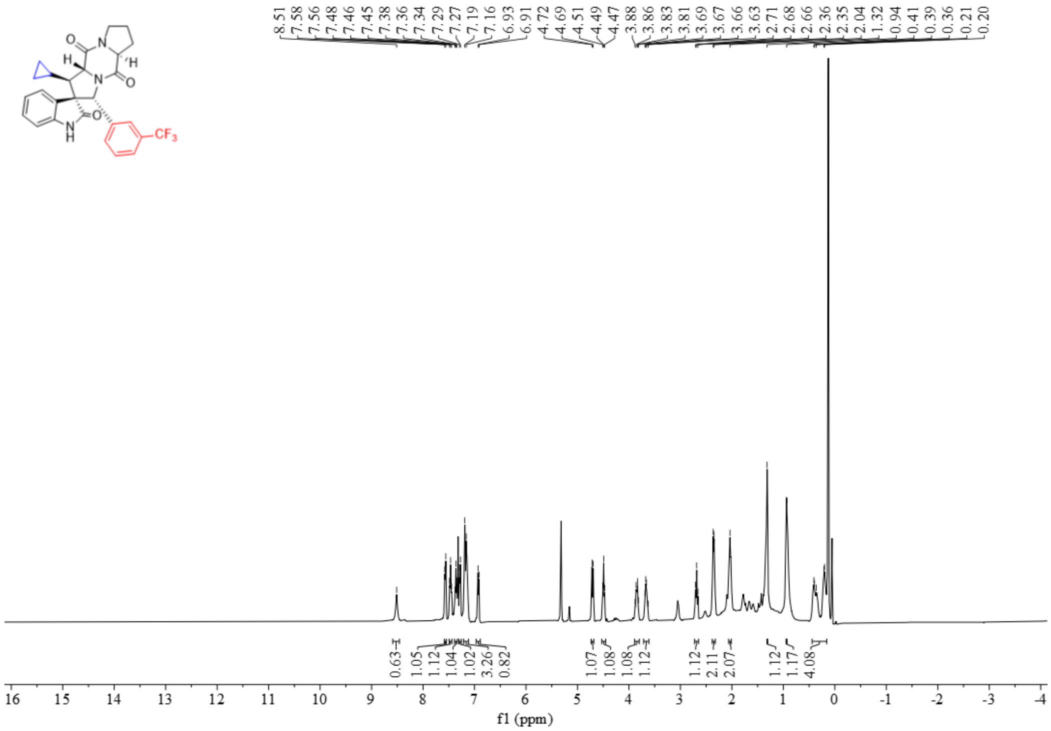

Figure S37. ^{1}H NMR spectrum of compound **4f'**

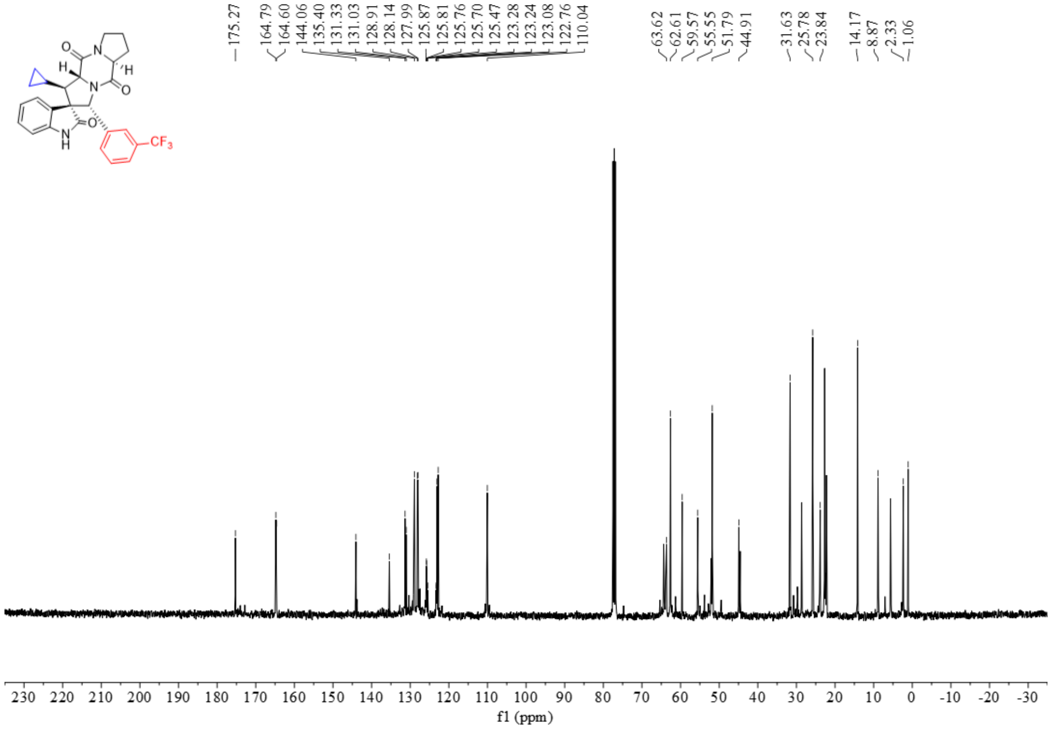

Figure S38. ^{13}C NMR spectrum of compound **4f'**

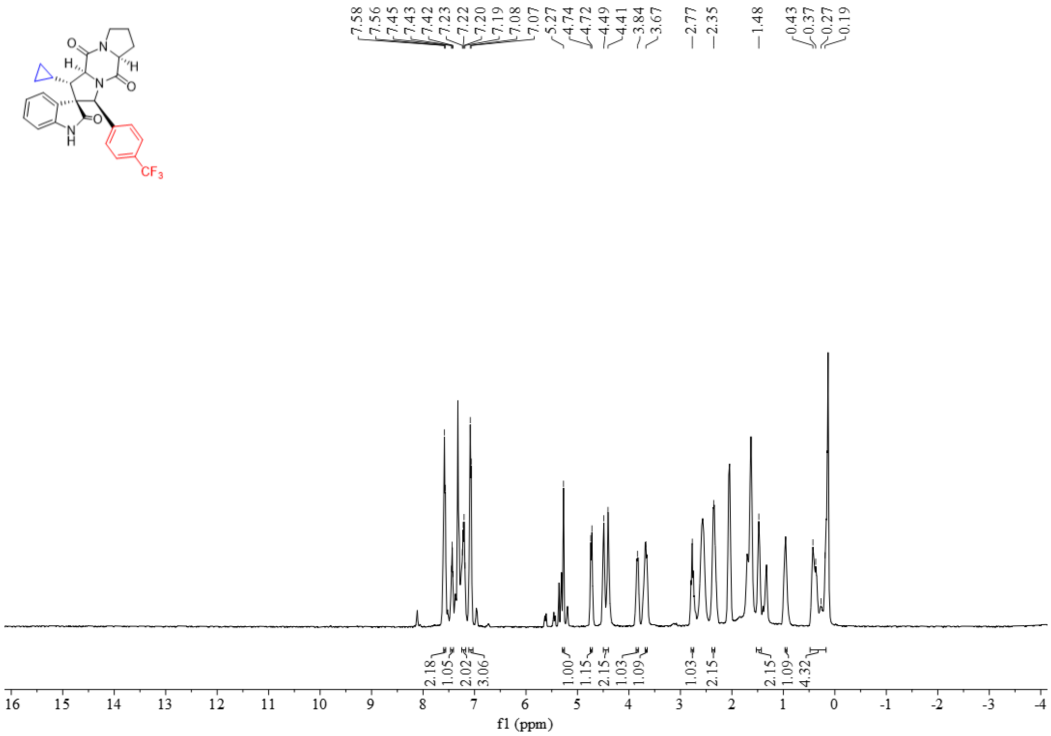

Figure S39. ^{1}H NMR spectrum of compound **4g**

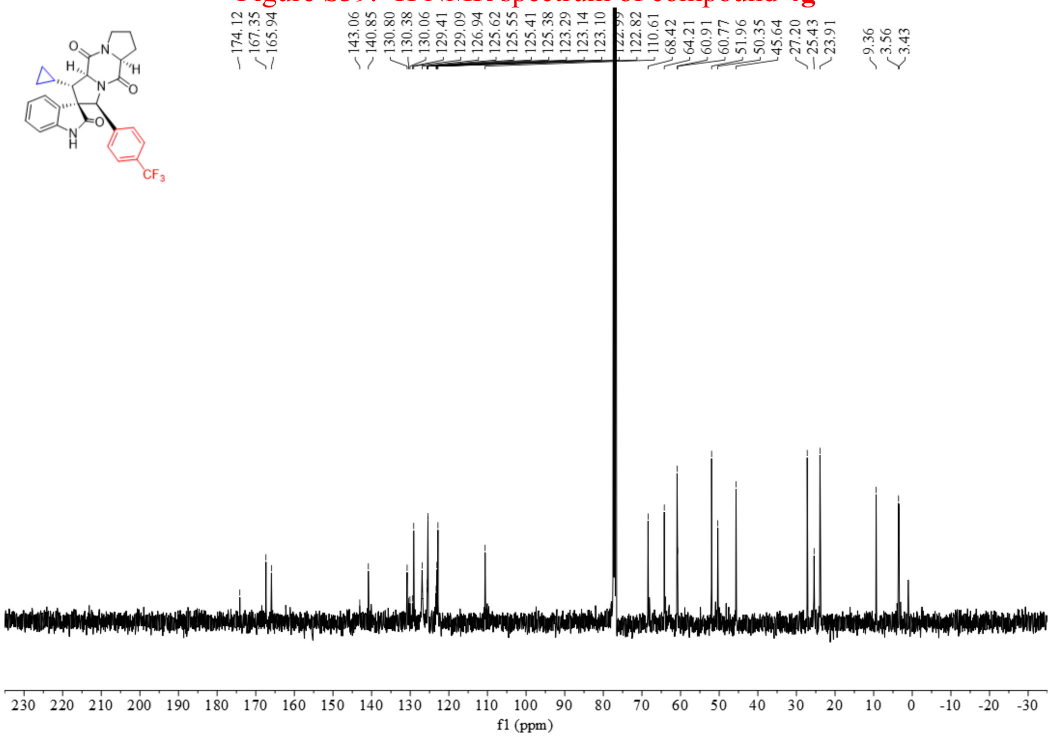

Figure S40. ^{13}C NMR spectrum of compound **4g**

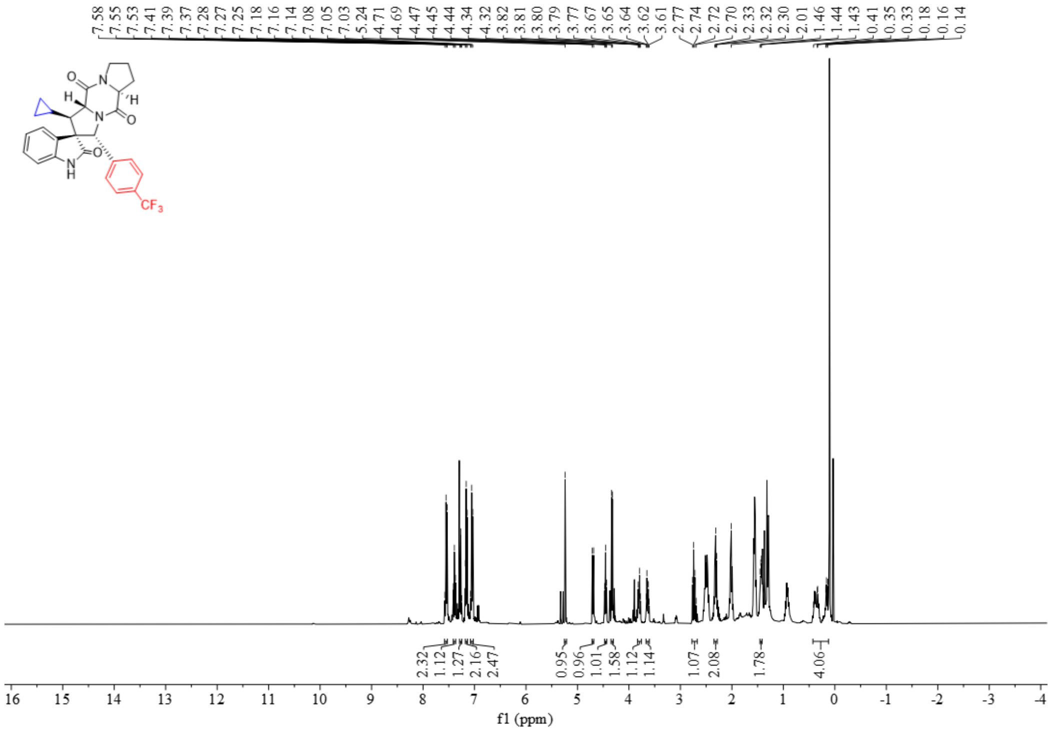

Figure S41. ^{1}H NMR spectrum of compound **4g'**

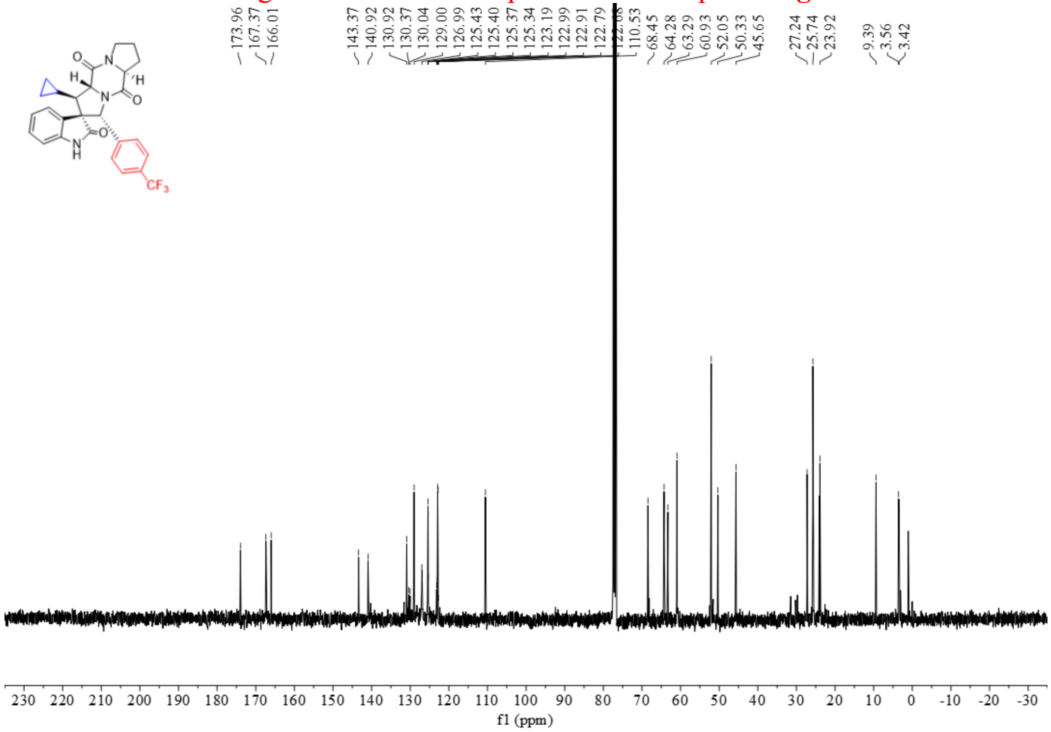

Figure S42. ^{13}C NMR spectrum of compound **4g'**

8. Heat map of the antifungal effect of different tested compounds on different pathogens

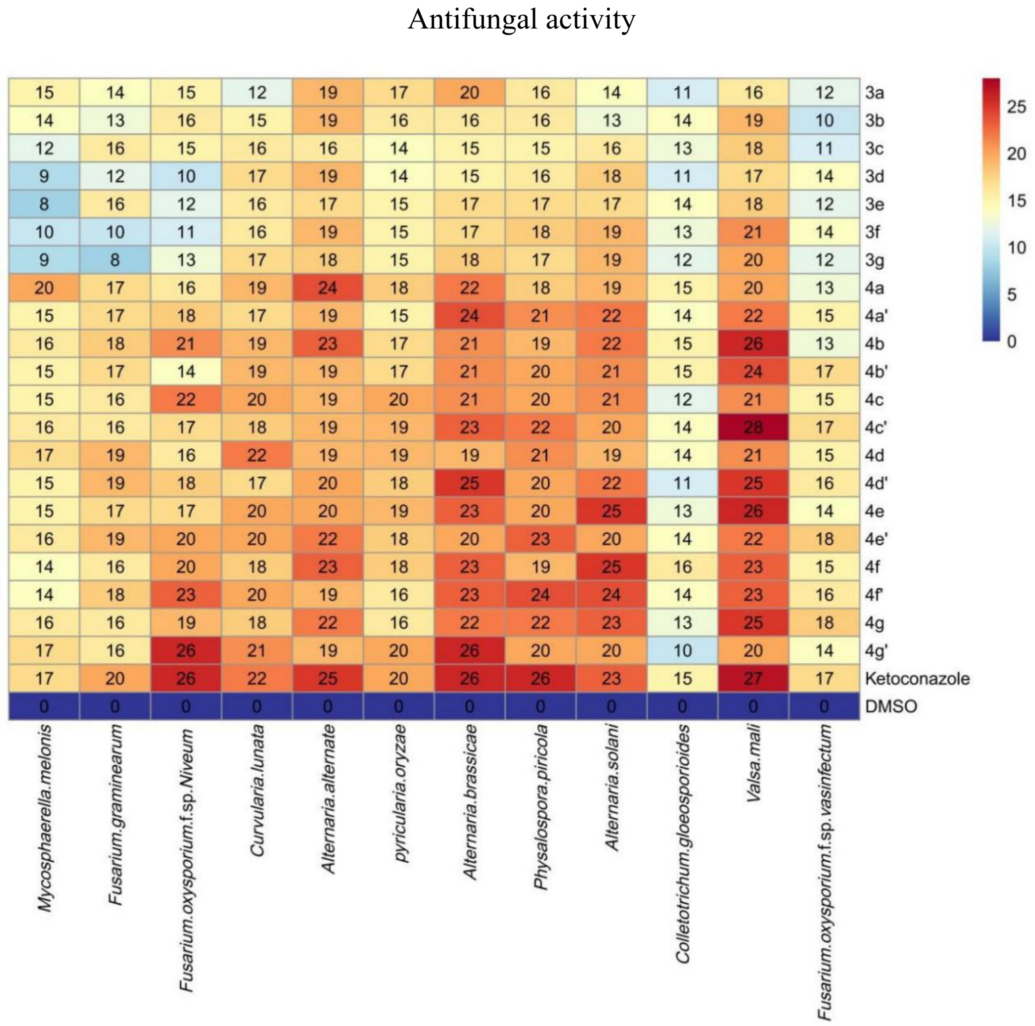

Figure S43. Heat map of the antifungal effect of different tested compounds on different pathogens

Table S1. Inhibition zone diameters (mm) of different tested compounds against phytopathogenic fungi.[a,b]

Comp-ound	*M.m.*	*F.g.*	*F.o.n.*	*C.l.*	*A.a.*	*P.o.*	*A.b.*	*P.p*	*A.s.*	*C.g.*	*V.m.*	*F.o.v.*
**3a**	**15±1.0**	**14±1.2**	**15±0.6**	**12±0.4**	**19±0.6**	**17±0.3**	**20±1.1**	**16±0.6**	**14±0.6**	**11±0.4**	**16±0.6**	**12±0.4**
**3b**	**14±0.6**	**13±0.6**	**16±0.0**	**15±1.1**	**19±1.0**	**16±1.1**	**16±0.6**	**16±1.1**	**13±1.0**	**14±1.0**	**19±0.6**	**10±1.0**
**3c**	**12±0.4**	**16±0.0**	**15±0.6**	**16±0.6**	**16±0.6**	**14±1.0**	**15±1.1**	**15±0.6**	**16±0.6**	**13±1.5**	**18±0.6**	**11±0.4**
**3d**	**9±0.4**	**12±0.4**	**10±1.7**	**17±0.6**	**19±1.5**	**14±0.6**	**15±0.6**	**16±0.6**	**18±0.6**	**11±1.5**	**17±0.3**	**14±1.1**
**3e**	**8±0.3**	**16±0.6**	**12±0.4**	**16±0.0**	**17±0.3**	**15±1.5**	**17±0.6**	**17±0.6**	**17±0.3**	**14±0.6**	**18±1.1**	**12±0.3**
**3f**	**10±0.4**	**10±0.6**	**11±1.0**	**16±0.6**	**19±0.6**	**15±0.6**	**17±0.3**	**18±0.6**	**19±0.6**	**13±0.6**	**21±1.5**	**14±1.0**
**3g**	**9±0.3**	**8±0.6**	**13±0.6**	**17±0.7**	**18±1.1**	**15±0.6**	**18±1.1**	**17±0.7**	**19±1.0**	**12±0.6**	**20±1.0**	**12±0.6**
**4a**	**20±0.6**	**17±0.7**	**16±0.6**	**19±1.5**	**24±1.7**	**18±1.1**	**22±0.6**	**18±1.0**	**19±0.6**	**15±1.1**	**20±1.5**	**13±0.4**
**4a'**	**15±0.6**	**17±1.5**	**18±1.1**	**17±0.0**	**19±0.6**	**15±0.4**	**24±1.7**	**21±0.6**	**22±1.5**	**14±0.6**	**22±0.6**	**15±1.1**
**4b**	**16±0.6**	**18±0.6**	**21±0.6**	**19±1.0**	**23±1.0**	**17±0.3**	**21±0.6**	**19±1.5**	**22±0.6**	**15±1.1**	**26±1.0**	**13±0.6**
**4b'**	**15±0.6**	**17±0.3**	**14±1.7**	**19±0.6**	**19±0.6**	**17±0.3**	**21±1.0**	**20±1.5**	**21±0.6**	**15±0.6**	**24±0.6**	**17±1.5**
**4c**	**15±0.6**	**16±0.6**	**22±0.6**	**20±1.5**	**19±0.6**	**20±1.5**	**21±0.4**	**20±1.1**	**21±0.6**	**12±0.6**	**21±0.6**	**15±1.5**
**4c'**	**16±0.3**	**16±1.1**	**17±0.3**	**18±1.1**	**19±1.0**	**19±1.0**	**23±1.5**	**22±1.0**	**20±0.6**	**14±1.2**	**28±1.5**	**17±0.3**
**4d**	**17±0.3**	**19±0.6**	**16±0.6**	**22±0.6**	**19±1.0**	**19±0.6**	**19±0.6**	**21±0.6**	**19±0.6**	**14±0.6**	**21±0.6**	**15±0.6**
**4d'**	**15±0.4**	**19±0.6**	**18±1.1**	**17±0.3**	**20±1.5**	**18±1.5**	**25±0.4**	**20±0.6**	**22±0.6**	**11±0.4**	**25±1.0**	**16±0.6**
**4e**	**15±0.6**	**17±0.3**	**17±0.7**	**20±1.5**	**20±0.6**	**19±1.0**	**23±1.5**	**20±1.1**	**25±2.1**	**13±1.0**	**26±1.5**	**14±0.6**
**4e'**	**16±0.4**	**19±1.0**	**20±1.1**	**20±1.1**	**22±1.5**	**18±0.6**	**20±0.6**	**23±1.5**	**20±0.6**	**14±0.6**	**22±0.6**	**18±1.1**
**4f**	**14±1.1**	**16±0.6**	**20±0.6**	**18±1.1**	**23±1.5**	**18±1.1**	**23±1.5**	**19±1.0**	**25±1.5**	**16±0.6**	**23±1.5**	**15±0.6**
**4f'**	**14±0.6**	**18±1.1**	**23±1.5**	**20±0.6**	**19±0.6**	**16±0.6**	**23±1.5**	**24±0.4**	**24±1.0**	**14±1.0**	**23±1.0**	**16±1.1**
**4g**	**16±0.0**	**16±0.6**	**19±1.5**	**18±1.1**	**22±1.5**	**16±1.1**	**22±0.6**	**22±1.5**	**23±0.6**	**13±1.5**	**25±0.6**	**18±0.6**
**4g'**	**17±0.3**	**16±0.0**	**26±0.4**	**21±0.6**	**19±0.4**	**20±0.6**	**26±1.0**	**20±1.5**	**20±1.1**	**10±0.6**	**20±0.6**	**14±1.0**
**Ketoconazole**	**17±0.7**	**20±1.0**	**26±0.6**	**22±1.7**	**25±0.6**	**20±1.1**	**26±1.5**	**26±0.6**	**23±1.0**	**15±1.1**	**27±1.0**	**17±0.7**
**DMSO**	**0**	**0**	**0**	**0**	**0**	**0**	**0**	**0**	**0**	**0**	**0**	**0**

[a]***M. m.***, *Mycosphaerella melonis*; ***F. g.***, *Fusarium graminearum*; ***F. o. n.***, *Fusarium oxysporum* f. sp. *niveum*; ***C. l.***, *Curvularia lunata*; ***A. a.***, *Alternaria alternata*; ***P. o.***, *Pyricularia oryzae*; ***A. b.***, *Alternaria brassicae*; ***P. p.***, *Physalospora piricola*; ***A. s.***, *Alternaria solani*; ***C. g.***, *Colletotrichum gloeosporioides*; ***V. m.***, *Valsa mali*; ***F. o. v.***, *Fusarium oxysporum* f. sp. *vasinfectum*.

[b]Values are presented as mean ± standard deviation (SD) of three independent experiments. Inhibition zone diameters are expressed in mm.

9. X-ray crystallography data for 4a and 4a'

X-ray crystallography data for 4a

Identification code	cu_hengqw240822_1
Empirical formula	$C_{53}H_{52}N_6O_6Cl_2$
Formula weight	939.90
Temperature/K	173.0
Crystal system	orthorhombic
Space group	$P2_12_12_1$

a/Å	13.1019(3)
b/Å	17.1656(4)
c/Å	20.9666(5)
α/°	90
β/°	90
γ/°	90
Volume/$Å^3$	4715.43(19)
Z	4
ρ_{calc}g/cm^3	1.324
μ/mm^{-1}	1.708
F(000)	1976.0
Crystal size/mm^3	0.32 × 0.26 × 0.11
Radiation	CuKα (λ = 1.54178)
2Θ range for data collection/°	6.654 to 127.4
Index ranges	$-15 \le h \le 13$, $-15 \le k \le 19$, $-24 \le l \le 23$
Reflections collected	43973
Independent reflections	7686 [R_{int} = 0.0377, R_{sigma} = 0.0307]
Data/restraints/parameters	7686/0/604
Goodness-of-fit on F^2	1.054
Final R indexes [I>=2σ (I)]	R_1 = 0.0401, wR_2 = 0.0989
Final R indexes [all data]	R_1 = 0.0425, wR_2 = 0.1008
Largest diff. peak/hole / e $Å^{-3}$	0.49/-0.41
Flack parameter	0.035(5)

Table S2 Fractional Atomic Coordinates (×10^4) and Equivalent Isotropic Displacement Parameters ($Å^2$×10^3) for cu_hengqw240822_1. U_{eq} is defined as 1/3 of the trace of the orthogonalised U_{IJ} tensor.

Atom	*x*	*y*	*z*	U(eq)
Cl2	4942.9(10)	6293.2(8)	-418.7(6)	70.5(4)
Cl1	4079.4(17)	4915.0(12)	161.3(9)	123.0(7)
O2	4465.7(19)	5645.7(12)	7498.4(10)	31.3(5)
O3	4168.2(18)	4816.6(12)	5162.8(10)	30.4(5)
O1	5918.0(16)	3021.4(13)	7295.1(10)	28.7(5)
O5	5162.5(19)	6828.8(14)	1346.6(11)	36.6(6)
O6	6931.1(18)	8711.4(13)	2944.1(11)	35.0(6)
O4	3943.4(18)	6202.4(15)	3673.7(12)	37.7(6)
N1	3931.6(19)	4448.6(13)	6193.0(11)	21.3(5)
N2	3921(2)	5961.1(14)	6501.8(12)	26.8(6)
N3	4815(2)	2047.9(15)	6993.5(13)	28.6(6)
N5	6329(2)	7794.4(16)	1491.7(13)	30.5(6)
N4	6286(2)	7503.8(15)	2745.4(12)	26.4(6)
N6	5275(2)	5699.2(16)	4251.4(13)	31.4(6)
C53	4211(4)	5906(4)	213(2)	73.7(16)

Table S2 Fractional Atomic Coordinates (×10⁴) and Equivalent Isotropic Displacement Parameters (Å²×10³) for cu_hengqw240822_1. U_{eq} is defined as 1/3 of the trace of the orthogonalised U_{IJ} tensor.

Atom	*x*	*y*	*z*	U(eq)
C23	4392(5)	6853(3)	5729(2)	65.1(14)
C24	3510(3)	6418(2)	5471.9(18)	44.1(10)
C25	3374(3)	5720.1(19)	5919.0(15)	29.0(7)
C26	3853(2)	4958.2(17)	5705.1(14)	24.0(7)
C7	4546(2)	3732.8(17)	6151.9(14)	21.6(6)
C8	4259(2)	3322.2(16)	6796.2(14)	20.9(6)
C9	3412(2)	2742.2(17)	6676.6(14)	23.9(7)
C14	3806(3)	1997.2(18)	6774.6(15)	26.3(7)
C13	3250(3)	1330(2)	6654.6(18)	39.3(9)
C12	2263(3)	1422(2)	6426(2)	44.4(10)
C21	4037(2)	5475.7(17)	6990.9(14)	23.1(6)
C20	3623(2)	4660.7(16)	6846.2(14)	21.5(6)
C1	5673(2)	3888.1(18)	6044.6(15)	27.2(7)
C6	6196(3)	3414(2)	5620.8(18)	39.2(9)
C5	7244(3)	3517(3)	5531(2)	53.3(12)
C4	7759(3)	4087(3)	5861(2)	56.1(13)
C3	7242(3)	4560(2)	6276(2)	46.0(10)
C2	6199(3)	4471(2)	6368.8(17)	34.7(8)
C15	5118(2)	2799.4(17)	7058.1(14)	23.3(7)
C11	1857(3)	2153(2)	6328.2(18)	40.2(9)
C10	2431(3)	2826(2)	6449.8(16)	30.7(7)
C16	4047(2)	4007.4(16)	7267.3(13)	20.2(6)
C17	3376(3)	3799.4(18)	7822.4(14)	25.0(7)
C19	3503(3)	4220.6(19)	8446.7(15)	29.8(7)
C18	3844(3)	3385.6(19)	8388.7(15)	33.1(8)
C22	4341(3)	6750.3(19)	6452.9(17)	41.0(9)
C49	7076(4)	8745(3)	881(2)	71.6(16)
C48	6159(3)	8241(2)	906.5(18)	42.3(9)
C47	5839(2)	7143.3(18)	1659.7(14)	26.8(7)
C46	6221(2)	6840.9(17)	2298.9(14)	24.2(6)
C42	5540(2)	6257.3(18)	2645.1(15)	25.8(7)
C34	5734(2)	6444.3(18)	3358.3(15)	25.7(7)
C35	6664(3)	6079.1(18)	3663.2(15)	26.7(7)
C40	6340(3)	5657.8(18)	4199.2(15)	28.0(7)
C39	7013(3)	5285.2(19)	4602.5(17)	35.1(8)
C38	8042(3)	5332(2)	4452.3(18)	38.6(9)
C50	7370(3)	8908(2)	1567.8(18)	39.4(9)
C51	7137(2)	8130.4(19)	1889.5(15)	28.4(7)
C52	6783(2)	8166.3(18)	2578.7(16)	27.9(7)
C33	5832(3)	7357.8(17)	3372.0(14)	25.9(7)

Table S2 Fractional Atomic Coordinates (×10⁴) and Equivalent Isotropic Displacement Parameters (Å²×10³) for cu_hengqw240822_1. U_{eq} is defined as 1/3 of the trace of the orthogonalised U_{IJ} tensor.

Atom	*x*	*y*	*z*	U(eq)
C27	4846(3)	7780.6(18)	3493.9(15)	29.6(7)
C32	4610(3)	7986(2)	4118.7(18)	42.8(9)
C31	3667(4)	8298(2)	4271(2)	55.5(12)
C30	2951(4)	8409(2)	3794(2)	53.5(11)
C29	3175(3)	8225(2)	3179(2)	46.1(10)
C28	4125(3)	7911(2)	3023.1(17)	34.8(8)
C36	7686(3)	6120.4(19)	3524.1(16)	32.0(8)
C37	8382(3)	5740(2)	3923.8(18)	36.3(8)
C41	4855(3)	6120.6(18)	3770.0(15)	28.8(7)
C43	5736(3)	5416.0(19)	2474.1(16)	33.1(8)
C44	4924(4)	4827(2)	2621(2)	53.7(11)
C45	5133(3)	5044(2)	1942.7(19)	46.2(9)

Table S3 Anisotropic Displacement Parameters (Å²×10³) for cu_hengqw240822_1. The Anisotropic displacement factor exponent takes the form: $-2\pi^2[h^2a^{*2}U_{11}+2hka^*b^*U_{12}+\ldots]$.

Atom	U_{11}	U_{22}	U_{33}	U_{23}	U_{13}	U_{12}
Cl2	70.0(8)	78.1(8)	63.4(7)	15.3(6)	10.5(6)	17.9(7)
Cl1	137.4(16)	115.6(14)	116.0(14)	-7.8(11)	46.7(13)	-52.5(13)
O2	48.7(14)	20.5(11)	24.6(12)	-3.5(9)	-3.6(10)	-6.5(10)
O3	41.9(14)	27.2(12)	22.1(11)	0.8(9)	1.3(10)	-1.2(10)
O1	24.5(12)	28.9(11)	32.5(12)	0.6(9)	-3.6(10)	1.0(10)
O5	38.7(14)	39.7(13)	31.5(12)	-4.4(10)	-7.9(11)	-0.4(11)
O6	38.7(14)	24.7(12)	41.6(13)	-2.8(11)	-6.2(11)	-6.0(10)
O4	28.3(14)	41.4(14)	43.4(14)	6.8(11)	5.2(11)	0.9(11)
N1	27.1(14)	16.7(12)	20.1(12)	-0.4(9)	0.3(10)	3.6(10)
N2	38.9(16)	16.5(12)	25.0(13)	-1.1(10)	0.3(12)	-1.6(11)
N3	32.1(15)	17.1(12)	36.6(15)	-0.3(11)	-2.3(12)	5.9(11)
N5	29.8(15)	33.1(15)	28.7(14)	8.6(12)	-2.6(12)	1.4(12)
N4	32.4(15)	22.9(13)	23.9(13)	0.9(10)	0.9(11)	-3.9(12)
N6	33.3(16)	32.8(15)	28.2(14)	8.0(12)	7.1(12)	-0.3(13)
C53	52(3)	129(5)	40(2)	-20(3)	2(2)	9(3)
C23	112(4)	41(2)	42(2)	13.1(19)	-6(2)	-24(3)
C24	74(3)	25.4(18)	32.8(19)	7.4(15)	-4.3(19)	11.6(18)
C25	35.6(19)	25.2(16)	26.1(16)	1.4(13)	-3.5(14)	4.6(14)
C26	26.1(17)	22.0(15)	23.9(16)	1.3(12)	-3.9(13)	-2.0(13)
C7	26.1(16)	17.0(14)	21.6(15)	-1.3(12)	0.0(12)	0.9(12)
C8	23.3(16)	15.4(14)	24.0(15)	0.9(11)	-0.9(12)	-0.7(12)

Table S3 Anisotropic Displacement Parameters (Å^2×10^3) for cu_hengqw240822_1. The Anisotropic displacement factor exponent takes the form: $-2\pi^2[h^2a^{*2}U_{11}+2hka^*b^*U_{12}+\ldots]$.

Atom	U_{11}	U_{22}	U_{33}	U_{23}	U_{13}	U_{12}
C9	26.8(17)	19.8(15)	25.2(15)	-2.2(12)	2.3(13)	-2.3(12)
C14	32.8(18)	18.0(15)	28.1(16)	-3.0(12)	4.7(13)	-1.9(14)
C13	47(2)	21.9(17)	49(2)	-7.0(15)	7.2(18)	-4.3(16)
C12	41(2)	34(2)	58(2)	-17.7(18)	6.9(19)	-15.7(17)
C21	27.6(17)	17.2(14)	24.6(16)	-0.6(12)	3.9(13)	2.1(13)
C20	24.7(16)	17.5(14)	22.2(15)	-1.9(11)	1.8(12)	1.1(13)
C1	28.7(18)	25.5(16)	27.6(16)	9.7(13)	2.2(13)	2.4(14)
C6	43(2)	28.4(18)	46(2)	8.6(15)	12.4(17)	8.6(16)
C5	41(2)	54(3)	64(3)	24(2)	23(2)	22(2)
C4	29(2)	68(3)	72(3)	44(3)	2(2)	7(2)
C3	33(2)	53(2)	53(2)	27(2)	-9.4(18)	-11.2(19)
C2	30.3(19)	35.4(18)	38.3(19)	8.2(15)	-3.8(15)	-7.6(15)
C15	27.0(18)	19.4(15)	23.5(15)	-0.1(12)	3.2(13)	2.0(13)
C11	29.1(19)	45(2)	47(2)	-14.4(17)	-0.9(16)	-8.2(17)
C10	29.2(18)	28.3(17)	34.8(18)	-7.0(14)	-2.7(14)	-0.3(14)
C16	24.8(16)	15.1(14)	20.7(14)	-0.6(11)	-0.5(13)	-0.4(12)
C17	31.0(18)	19.9(15)	24.1(16)	0.9(12)	2.5(13)	-1.0(13)
C19	39(2)	23.2(16)	27.2(17)	-2.0(13)	3.2(15)	0.8(14)
C18	47(2)	25.3(17)	27.0(16)	4.7(13)	3.0(15)	0.6(15)
C22	70(3)	18.5(16)	34.3(19)	1.4(14)	1.4(18)	-7.1(17)
C49	62(3)	82(4)	71(3)	52(3)	-15(3)	-19(3)
C48	44(2)	47(2)	35.4(19)	20.9(17)	-1.9(17)	4.9(18)
C47	27.6(17)	28.9(17)	23.9(16)	-0.8(13)	3.0(14)	1.9(14)
C46	25.3(16)	21.0(15)	26.4(15)	-0.4(12)	1.9(13)	0.0(13)
C42	27.7(17)	23.2(16)	26.3(16)	1.3(13)	2.7(13)	-2.7(13)
C34	27.7(17)	22.9(16)	26.5(16)	2.6(12)	-0.2(13)	0.0(13)
C35	30.9(18)	22.1(15)	27.2(16)	0.4(13)	-2.3(14)	1.2(13)
C40	34.9(19)	21.2(15)	27.8(16)	1.1(13)	0.6(14)	0.6(14)
C39	47(2)	28.5(17)	29.9(18)	4.4(14)	-0.2(16)	3.1(16)
C38	43(2)	31.3(18)	41(2)	0.6(15)	-11.6(17)	8.8(17)
C50	31.8(19)	34.8(19)	52(2)	16.9(17)	4.9(17)	0.3(16)
C51	23.7(16)	27.5(16)	33.9(18)	7.2(13)	0.2(14)	2.0(13)
C52	23.0(17)	24.3(17)	36.4(18)	7.6(14)	-3.9(14)	-0.5(13)
C33	30.0(17)	24.2(16)	23.3(15)	2.3(12)	0.4(13)	-2.8(13)
C27	37.9(19)	22.4(16)	28.4(17)	0.3(13)	2.5(15)	-0.6(14)
C32	59(3)	37(2)	31.8(19)	-1.8(16)	5.4(17)	9.9(18)
C31	72(3)	44(2)	51(2)	-5.4(19)	23(2)	17(2)
C30	51(3)	36(2)	73(3)	5(2)	24(2)	12.9(19)
C29	38(2)	36(2)	65(3)	9.0(19)	-2.5(19)	5.2(17)
C28	40(2)	29.0(17)	35.8(18)	2.2(14)	0.5(16)	1.0(16)

Table S3 Anisotropic Displacement Parameters (Å^2×10^3) for cu_hengqw240822_1. The Anisotropic displacement factor exponent takes the form: $-2\pi^2[h^2a^{*2}U_{11}+2hka^*b^*U_{12}+\ldots]$.

Atom	U_{11}	U_{22}	U_{33}	U_{23}	U_{13}	U_{12}
C36	34.1(19)	26.8(17)	35.3(18)	2.2(14)	3.7(15)	-0.1(15)
C37	29.1(19)	33.1(18)	47(2)	-4.3(16)	-3.3(16)	3.9(15)
C41	33(2)	23.3(16)	29.9(17)	0.7(13)	2.8(15)	-1.0(14)
C43	39(2)	24.9(17)	35.3(18)	-1.2(14)	6.0(15)	-4.5(15)
C44	65(3)	28(2)	69(3)	-4.5(18)	19(2)	-15.9(19)
C45	52(2)	33.0(19)	54(2)	-13.5(17)	-1(2)	-10.7(18)

Table S4 Bond Lengths for cu_hengqw240822_1.

Atom	Atom	Length/Å	Atom	Atom	Length/Å
Cl2	C53	1.764(5)	C20	C16	1.531(4)
Cl1	C53	1.713(7)	C1	C6	1.386(5)
O2	C21	1.238(4)	C1	C2	1.392(5)
O3	C26	1.234(4)	C6	C5	1.397(6)
O1	C15	1.221(4)	C5	C4	1.376(7)
O5	C47	1.228(4)	C4	C3	1.370(7)
O6	C52	1.225(4)	C3	C2	1.389(5)
O4	C41	1.220(4)	C11	C10	1.402(5)
N1	C26	1.350(4)	C16	C17	1.502(4)
N1	C7	1.471(4)	C17	C19	1.505(4)
N1	C20	1.474(4)	C17	C18	1.513(4)
N2	C25	1.476(4)	C19	C18	1.506(5)
N2	C21	1.330(4)	C49	C48	1.481(6)
N2	C22	1.465(4)	C49	C50	1.517(7)
N3	C14	1.402(4)	C47	C46	1.522(4)
N3	C15	1.357(4)	C46	C42	1.525(4)
N5	C48	1.463(4)	C42	C34	1.550(4)
N5	C47	1.336(4)	C42	C43	1.510(5)
N5	C51	1.466(4)	C34	C35	1.512(5)
N4	C46	1.476(4)	C34	C33	1.573(4)
N4	C52	1.356(4)	C34	C41	1.542(4)
N4	C33	1.464(4)	C35	C40	1.402(4)
N6	C40	1.401(4)	C35	C36	1.373(5)
N6	C41	1.358(4)	C40	C39	1.378(5)
C23	C24	1.477(6)	C39	C38	1.387(5)
C23	C22	1.529(6)	C38	C37	1.384(5)
C24	C25	1.532(5)	C50	C51	1.526(5)
C25	C26	1.518(4)	C51	C52	1.519(5)
C7	C8	1.569(4)	C33	C27	1.503(5)

Table S4 Bond Lengths for cu_hengqw240822_1.					
Atom	Atom	Length/Å	Atom	Atom	Length/Å
C7	C1	1.518(4)	C27	C32	1.391(5)
C8	C9	1.512(4)	C27	C28	1.385(5)
C8	C15	1.541(4)	C32	C31	1.384(6)
C8	C16	1.561(4)	C31	C30	1.384(7)
C9	C14	1.395(4)	C30	C29	1.361(6)
C9	C10	1.378(5)	C29	C28	1.396(5)
C14	C13	1.381(5)	C36	C37	1.400(5)
C13	C12	1.389(6)	C43	C44	1.499(5)
C12	C11	1.377(6)	C43	C45	1.507(5)
C21	C20	1.531(4)	C44	C45	1.495(6)

Table S5 Bond Angles for cu_hengqw240822_1.							
Atom	Atom	Atom	Angle/°	Atom	Atom	Atom	Angle/°
C26	N1	C7	122.6(2)	C17	C16	C20	114.1(3)
C26	N1	C20	121.6(2)	C16	C17	C19	119.7(3)
C7	N1	C20	114.2(2)	C16	C17	C18	118.8(3)
C21	N2	C25	121.2(3)	C19	C17	C18	59.9(2)
C21	N2	C22	126.2(3)	C17	C19	C18	60.3(2)
C22	N2	C25	112.5(3)	C19	C18	C17	59.8(2)
C15	N3	C14	111.6(3)	N2	C22	C23	101.1(3)
C48	N5	C51	112.4(3)	C48	C49	C50	106.2(3)
C47	N5	C48	125.9(3)	N5	C48	C49	102.3(3)
C47	N5	C51	121.7(3)	O5	C47	N5	125.0(3)
C52	N4	C46	120.7(3)	O5	C47	C46	123.9(3)
C52	N4	C33	124.8(3)	N5	C47	C46	111.1(3)
C33	N4	C46	114.4(2)	N4	C46	C47	108.3(2)
C41	N6	C40	111.9(3)	N4	C46	C42	103.8(2)
Cl1	C53	Cl2	112.4(3)	C47	C46	C42	116.8(3)
C24	C23	C22	105.6(4)	C46	C42	C34	103.2(2)
C23	C24	C25	105.2(3)	C43	C42	C46	114.6(3)
N2	C25	C24	103.3(3)	C43	C42	C34	113.5(3)
N2	C25	C26	106.6(3)	C42	C34	C33	103.7(2)
C26	C25	C24	116.4(3)	C35	C34	C42	117.0(3)
O3	C26	N1	123.1(3)	C35	C34	C33	109.9(3)
O3	C26	C25	125.5(3)	C35	C34	C41	102.4(2)
N1	C26	C25	111.5(3)	C41	C34	C42	110.1(3)
N1	C7	C8	101.2(2)	C41	C34	C33	114.2(3)
N1	C7	C1	113.2(2)	C40	C35	C34	108.0(3)
C1	C7	C8	116.1(2)	C36	C35	C34	132.5(3)
C9	C8	C7	109.2(2)	C36	C35	C40	119.5(3)

Table S5 Bond Angles for cu_hengqw240822_1.

Atom	Atom	Atom	Angle/°	Atom	Atom	Atom	Angle/°
C9	C8	C15	102.2(2)	N6	C40	C35	109.7(3)
C9	C8	C16	118.1(3)	C39	C40	N6	127.7(3)
C15	C8	C7	113.2(2)	C39	C40	C35	122.5(3)
C15	C8	C16	110.1(2)	C40	C39	C38	117.1(3)
C16	C8	C7	104.4(2)	C37	C38	C39	121.6(3)
C14	C9	C8	107.9(3)	C49	C50	C51	102.0(3)
C10	C9	C8	132.3(3)	N5	C51	C50	103.7(3)
C10	C9	C14	119.5(3)	N5	C51	C52	109.7(3)
C9	C14	N3	109.9(3)	C52	C51	C50	116.5(3)
C13	C14	N3	127.5(3)	O6	C52	N4	123.7(3)
C13	C14	C9	122.6(3)	O6	C52	C51	125.3(3)
C14	C13	C12	117.3(3)	N4	C52	C51	111.0(3)
C11	C12	C13	121.0(3)	N4	C33	C34	100.8(2)
O2	C21	N2	124.5(3)	N4	C33	C27	114.8(3)
O2	C21	C20	123.1(3)	C27	C33	C34	114.5(3)
N2	C21	C20	112.3(3)	C32	C27	C33	118.3(3)
N1	C20	C21	108.2(2)	C28	C27	C33	122.9(3)
N1	C20	C16	104.8(2)	C28	C27	C32	118.6(3)
C21	C20	C16	115.2(2)	C31	C32	C27	120.9(4)
C6	C1	C7	118.2(3)	C30	C31	C32	119.5(4)
C6	C1	C2	119.4(3)	C29	C30	C31	120.4(4)
C2	C1	C7	122.4(3)	C30	C29	C28	120.3(4)
C1	C6	C5	119.9(4)	C27	C28	C29	120.3(3)
C4	C5	C6	120.3(4)	C35	C36	C37	118.9(3)
C3	C4	C5	119.9(4)	C38	C37	C36	120.4(3)
C4	C3	C2	120.7(4)	O4	C41	N6	125.5(3)
C3	C2	C1	119.9(4)	O4	C41	C34	126.6(3)
O1	C15	N3	126.1(3)	N6	C41	C34	107.8(3)
O1	C15	C8	126.2(3)	C44	C43	C42	118.4(3)
N3	C15	C8	107.7(3)	C44	C43	C45	59.6(3)
C12	C11	C10	121.1(3)	C45	C43	C42	119.4(3)
C9	C10	C11	118.5(3)	C45	C44	C43	60.5(2)
C20	C16	C8	104.6(2)	C44	C45	C43	59.9(3)
C17	C16	C8	114.5(2)				

Table S6 Torsion Angles for cu_hengqw240822_1.

A	B	C	D	Angle/°	A	B	C	D	Angle/°
O2	C21	C20	N1	-134.9(3)	C16	C8	C9	C14	-128.2(3)
O2	C21	C20	C16	-18.0(4)	C16	C8	C9	C10	58.3(4)
O5	C47	C46	N4	-133.7(3)	C16	C8	C15	O1	-44.3(4)

Table S6 Torsion Angles for cu_hengqw240822_1.

A	B	C	D	Angle/˚	A	B	C	D	Angle/˚
O5	C47	C46	C42	-17.0(4)	C16	C8	C15	N3	134.2(3)
N1	C7	C8	C9	95.9(3)	C16	C17	C19	C18	-108.1(3)
N1	C7	C8	C15	-151.0(2)	C16	C17	C18	C19	109.5(3)
N1	C7	C8	C16	-31.3(3)	C22	N2	C25	C24	2.1(4)
N1	C7	C1	C6	-139.1(3)	C22	N2	C25	C26	125.3(3)
N1	C7	C1	C2	42.8(4)	C22	N2	C21	O2	5.1(5)
N1	C20	C16	C8	-18.5(3)	C22	N2	C21	C20	-172.2(3)
N1	C20	C16	C17	-144.4(3)	C22	C23	C24	C25	-33.7(5)
N2	C25	C26	O3	-128.9(3)	C49	C50	C51	N5	-27.2(4)
N2	C25	C26	N1	48.9(3)	C49	C50	C51	C52	-147.8(3)
N2	C21	C20	N1	42.4(3)	C48	N5	C47	O5	-0.8(5)
N2	C21	C20	C16	159.3(3)	C48	N5	C47	C46	179.8(3)
N3	C14	C13	C12	-178.7(3)	C48	N5	C51	C50	8.7(4)
N5	C47	C46	N4	45.7(3)	C48	N5	C51	C52	133.7(3)
N5	C47	C46	C42	162.4(3)	C48	C49	C50	C51	37.3(5)
N5	C51	C52	O6	-138.5(3)	C47	N5	C48	C49	-164.5(4)
N5	C51	C52	N4	42.3(3)	C47	N5	C51	C50	-172.6(3)
N4	C46	C42	C34	-28.0(3)	C47	N5	C51	C52	-47.5(4)
N4	C46	C42	C43	-151.9(3)	C47	C46	C42	C34	-147.1(3)
N4	C33	C27	C32	-150.1(3)	C47	C46	C42	C43	89.0(3)
N4	C33	C27	C28	35.9(4)	C46	N4	C52	O6	-174.2(3)
N6	C40	C39	C38	-179.5(3)	C46	N4	C52	C51	5.0(4)
C23	C24	C25	N2	19.6(4)	C46	N4	C33	C34	15.4(3)
C23	C24	C25	C26	-96.8(4)	C46	N4	C33	C27	-108.1(3)
C24	C23	C22	N2	33.7(4)	C46	C42	C34	C35	-83.3(3)
C24	C25	C26	O3	-14.3(5)	C46	C42	C34	C33	37.8(3)
C24	C25	C26	N1	163.5(3)	C46	C42	C34	C41	160.3(2)
C25	N2	C21	O2	-178.2(3)	C46	C42	C43	C44	-161.2(3)
C25	N2	C21	C20	4.5(4)	C46	C42	C43	C45	-92.0(4)
C25	N2	C22	C23	-21.8(4)	C42	C34	C35	C40	-124.2(3)
C26	N1	C7	C8	-173.2(3)	C42	C34	C35	C36	58.7(5)
C26	N1	C7	C1	61.9(4)	C42	C34	C33	N4	-32.1(3)
C26	N1	C20	C21	-44.3(4)	C42	C34	C33	C27	91.7(3)
C26	N1	C20	C16	-167.8(3)	C42	C34	C41	O4	-48.4(4)
C7	N1	C26	O3	10.3(5)	C42	C34	C41	N6	129.4(3)
C7	N1	C26	C25	-167.5(3)	C42	C43	C44	C45	109.3(4)
C7	N1	C20	C21	121.5(3)	C42	C43	C45	C44	-107.6(4)
C7	N1	C20	C16	-1.9(3)	C34	C42	C43	C44	80.6(4)
C7	C8	C9	C14	112.8(3)	C34	C42	C43	C45	149.8(3)
C7	C8	C9	C10	-60.7(4)	C34	C35	C40	N6	2.1(4)
C7	C8	C15	O1	72.1(4)	C34	C35	C40	C39	-176.7(3)
C7	C8	C15	N3	-109.4(3)	C34	C35	C36	C37	176.6(3)

Table S6 Torsion Angles for cu_hengqw240822_1.

A	B	C	D	Angle/˚	A	B	C	D	Angle/˚
C7	C8	C16	C20	31.3(3)	C34	C33	C27	C32	93.9(4)
C7	C8	C16	C17	156.9(3)	C34	C33	C27	C28	-80.0(4)
C7	C1	C6	C5	-177.2(3)	C35	C34	C33	N4	93.7(3)
C7	C1	C2	C3	176.6(3)	C35	C34	C33	C27	-142.6(3)
C8	C7	C1	C6	104.5(3)	C35	C34	C41	O4	-173.5(3)
C8	C7	C1	C2	-73.6(4)	C35	C34	C41	N6	4.3(3)
C8	C9	C14	N3	4.4(3)	C35	C40	C39	C38	-0.9(5)
C8	C9	C14	C13	-174.7(3)	C35	C36	C37	C38	-0.3(5)
C8	C9	C10	C11	173.3(3)	C40	N6	C41	O4	174.4(3)
C8	C16	C17	C19	150.4(3)	C40	N6	C41	C34	-3.4(4)
C8	C16	C17	C18	80.7(3)	C40	C35	C36	C37	-0.1(5)
C9	C8	C15	O1	-170.6(3)	C40	C39	C38	C37	0.5(5)
C9	C8	C15	N3	7.9(3)	C39	C38	C37	C36	0.1(5)
C9	C8	C16	C20	-90.1(3)	C50	C49	C48	N5	-31.7(5)
C9	C8	C16	C17	35.5(4)	C50	C51	C52	O6	-21.2(5)
C9	C14	C13	C12	0.2(5)	C50	C51	C52	N4	159.6(3)
C14	N3	C15	O1	172.7(3)	C51	N5	C48	C49	14.2(4)
C14	N3	C15	C8	-5.8(3)	C51	N5	C47	O5	-179.4(3)
C14	C9	C10	C11	0.4(5)	C51	N5	C47	C46	1.2(4)
C14	C13	C12	C11	-0.5(6)	C52	N4	C46	C47	-50.8(4)
C13	C12	C11	C10	0.7(6)	C52	N4	C46	C42	-175.6(3)
C12	C11	C10	C9	-0.6(5)	C52	N4	C33	C34	-161.0(3)
C21	N2	C25	C24	-175.0(3)	C52	N4	C33	C27	75.4(4)
C21	N2	C25	C26	-51.8(4)	C33	N4	C46	C47	132.6(3)
C21	N2	C22	C23	155.1(4)	C33	N4	C46	C42	7.8(3)
C21	C20	C16	C8	-137.4(3)	C33	N4	C52	O6	2.1(5)
C21	C20	C16	C17	96.8(3)	C33	N4	C52	C51	-178.8(3)
C20	N1	C26	O3	175.0(3)	C33	C34	C35	C40	117.9(3)
C20	N1	C26	C25	-2.9(4)	C33	C34	C35	C36	-59.1(5)
C20	N1	C7	C8	21.1(3)	C33	C34	C41	O4	67.8(4)
C20	N1	C7	C1	-103.8(3)	C33	C34	C41	N6	-114.4(3)
C20	C16	C17	C19	-89.1(3)	C33	C27	C32	C31	-172.8(3)
C20	C16	C17	C18	-158.9(3)	C33	C27	C28	C29	172.5(3)
C1	C7	C8	C9	-141.2(3)	C27	C32	C31	C30	-0.2(6)
C1	C7	C8	C15	-28.0(4)	C32	C27	C28	C29	-1.5(5)
C1	C7	C8	C16	91.6(3)	C32	C31	C30	C29	-1.0(6)
C1	C6	C5	C4	0.1(6)	C31	C30	C29	C28	0.9(6)
C6	C1	C2	C3	-1.5(5)	C30	C29	C28	C27	0.3(6)
C6	C5	C4	C3	-0.6(6)	C28	C27	C32	C31	1.4(5)
C5	C4	C3	C2	0.1(6)	C36	C35	C40	N6	179.5(3)
C4	C3	C2	C1	0.9(5)	C36	C35	C40	C39	0.8(5)
C2	C1	C6	C5	1.0(5)	C41	N6	C40	C35	0.9(4)

Table S6 Torsion Angles for cu_hengqw240822_1.

A	B	C	D	Angle/˚	A	B	C	D	Angle/˚
C15	N3	C14	C9	1.0(4)	C41	N6	C40	C39	179.6(3)
C15	N3	C14	C13	180.0(3)	C41	C34	C35	C40	-3.8(3)
C15	C8	C9	C14	-7.3(3)	C41	C34	C35	C36	179.2(4)
C15	C8	C9	C10	179.2(3)	C41	C34	C33	N4	-151.9(3)
C15	C8	C16	C20	153.1(2)	C41	C34	C33	C27	-28.2(4)
C15	C8	C16	C17	-81.3(3)	C43	C42	C34	C35	41.3(4)
C10	C9	C14	N3	178.9(3)	C43	C42	C34	C33	162.4(3)
C10	C9	C14	C13	-0.2(5)	C43	C42	C34	C41	-75.0(3)

Table S7 Hydrogen Atom Coordinates (Å×10^4) and Isotropic Displacement Parameters (Å^2×10^3) for cu_hengqw240822_1.

Atom	*x*	*y*	*z*	U(eq)
H3	5203.46	1642.68	7078.43	34
H6A	4921.41	5478.74	4559.06	38
H53A	4542.09	6038.92	623.03	88
H53B	3526.35	6149.67	208.92	88
H23A	4347.01	7410.79	5613.32	78
H23B	5039.66	6639.28	5559.47	78
H24A	3647.75	6241.64	5030.7	53
H24B	2890.63	6748.2	5470.89	53
H25	2633.86	5640.47	6014.94	35
H7	4283	3409.29	5790.78	26
H13	3531.56	826.94	6725.69	47
H12	1860.91	974.92	6335.28	53
H20	2861.59	4665.96	6875.63	26
H6	5842.34	3019.69	5392.02	47
H5	7601.83	3192.66	5239.8	64
H4	8473.07	4151.75	5801.6	67
H3A	7600.55	4953.45	6503.07	55
H2	5844.54	4806.69	6652.92	42
H11	1176.98	2201.36	6175.83	48
H10	2149.58	3328.63	6377.62	37
H16	4718.51	4185.66	7441.26	24
H17	2662.12	3642.43	7712.56	30
H19A	2880.72	4328.43	8699.91	36
H19B	4036.98	4627.18	8473.03	36
H18A	3430	2982.04	8605.26	40
H18B	4586.71	3280.9	8378.29	40
H22A	5025.41	6785.77	6650.21	49
H22B	3882.74	7139.52	6651.82	49

Table S7 Hydrogen Atom Coordinates (Å×10^4) and Isotropic Displacement Parameters (Å^2×10^3) for cu_hengqw240822_1.				
**Atom**	***x***	***y***	***z***	**U(eq)**
H49A	6920.84	9236.72	654.59	86
H49B	7638.85	8476.59	655.38	86
H48A	5526.65	8553.94	933.6	51
H48B	6120.67	7894.55	529.5	51
H46	6915.34	6609.97	2241.66	29
H42	4812.57	6381.94	2543.93	31
H39	6781.3	5008.47	4967.25	42
H38	8525.22	5078.01	4718.52	46
H50A	6953.53	9333.75	1750.96	47
H50B	8101.94	9043.28	1605	47
H51	7755.47	7790.35	1862.99	34
H33	6334.89	7507.16	3709.06	31
H32	5102.46	7910.52	4445.4	51
H31	3512.64	8434.93	4699.04	67
H30	2298.63	8615.94	3897.79	64
H29	2682.12	8310.48	2853.96	55
H28	4277.99	7786.28	2592.09	42
H36	7917.98	6402.73	3162.37	38
H37	9090.99	5761.74	3831.76	44
H43	6456.24	5232.74	2522.65	40
H44A	5145.73	4305.25	2766.16	64
H44B	4282.14	5017.08	2815.12	64
H45A	4621.03	5369.28	1719.46	55
H45B	5484.95	4657.19	1670.48	55

X-ray crystallography data for 4a'	
Identification code	cu_hengqw240724_1
Empirical formula	$C_{26}H_{27}N_3O_4$
Formula weight	445.50
Temperature/K	173.02
Crystal system	trigonal
Space group	R3
a/Å	16.9221(8)
b/Å	16.9221(8)
c/Å	19.7296(17)
α/°	90
β/°	90
γ/°	120
Volume/Å^3	4892.8(6)
Z	9
ρ_{calc}g/cm^3	1.361

μ/mm^{-1}	0.093
F(000)	2124.0
Crystal size/mm^3	0.24 × 0.2 × 0.15
Radiation	Mo Kα (λ = 1.54178)
2Θ range for data collection/°	7.514 to 127.292
Index ranges	-19 ≤ h ≤ 19, -19 ≤ k ≤ 19, -22 ≤ l ≤ 22
Reflections collected	27429
Independent reflections	3544 [R_{int} = 0.0480, R_{sigma} = 0.0303]
Data/restraints/parameters	3544/37/291
Goodness-of-fit on F^2	1.077
Final R indexes [I>=2σ (I)]	R_1 = 0.0319, wR_2 = 0.0747
Final R indexes [all data]	R_1 = 0.0339, wR_2 = 0.0763
Largest diff. peak/hole / e $Å^{-3}$	0.21/-0.17
Flack parameter	0.1(3)

Table S8 Fractional Atomic Coordinates (×10^4) and Equivalent Isotropic Displacement Parameters ($Å^2$×10^3) for cu_hengqw240724_1. U_{eq} is defined as 1/3 of the trace of the orthogonalised U_{IJ} tensor.

Atom	*x*	*y*	*z*	U(eq)
O1	1975.2(14)	662.4(13)	4929.1(12)	52.5(6)
O2	5272.8(13)	3460.1(15)	4248.2(11)	45.6(5)
O3	3546.0(14)	3802.6(14)	6595.4(11)	46.1(5)
N1	3427.7(15)	1155.1(15)	4619.1(12)	37.1(5)
N2	3920.3(14)	2937.9(14)	4791.4(11)	30.8(5)
N3	3025.3(15)	4666.7(16)	6077.0(12)	37.8(5)
C2	4001(2)	474(2)	3864.5(18)	56.0(9)
C1	3228(2)	253(2)	4370.2(17)	47.4(7)
C6	2747.2(18)	1292.3(18)	4808.1(14)	35.6(6)
C7	2944.0(16)	2269.2(16)	4810.3(13)	29.6(5)
C8	2610.2(16)	2583.6(17)	5414.3(13)	28.4(6)
C9	3200.7(16)	3645.0(16)	5371.9(13)	28.6(6)
C12	2785.2(16)	4122.5(16)	4990.0(13)	30.0(6)
C13	2531.4(17)	4083.0(19)	4319.8(14)	36.9(6)
C14	2172.1(19)	4628(2)	4108.2(15)	44.0(7)
C17	2091.4(19)	5210(2)	4558.8(18)	46.6(8)
C5	4533.9(17)	2818.2(19)	4440.7(13)	35.2(6)
C4	4341.8(18)	1859(2)	4390.3(14)	37.1(6)
C3	4411(2)	1490(2)	3703.7(16)	48.5(8)
C10	4131.3(17)	3836.8(18)	5045.8(14)	33.6(6)
C15	2352(2)	5266.3(19)	5226.9(16)	42.9(7)
C16	2696.5(17)	4717.7(17)	5434.8(14)	33.9(6)

Table S8 Fractional Atomic Coordinates (×10⁴) and Equivalent Isotropic Displacement Parameters (Å²×10³) for cu_hengqw240724_1. U_{eq} is defined as 1/3 of the trace of the orthogonalised U_{IJ} tensor.

Atom	*x*	*y*	*z*	U(eq)
C11	3297.1(17)	4035.7(18)	6091.6(14)	33.7(6)
C24	1595.0(17)	2230.0(17)	5431.4(14)	32.0(6)
C26	981.7(19)	1440.3(19)	5875.3(15)	38.6(6)
C25	1173(2)	2384(2)	6052.0(16)	45.4(7)
C18	4955.5(19)	4238(2)	5510.1(16)	43.8(7)
C23	5540(2)	5168(2)	5511.5(19)	56.8(8)
C22	6316(3)	5553(3)	5913(2)	79.0(11)
C21	6490(3)	5002(4)	6320(3)	87.1(12)
C20	5903(3)	4080(4)	6334(3)	92.1(14)
C19	5139(3)	3699(3)	5926(2)	67.5(10)

Table S9 Anisotropic Displacement Parameters (Å²×10³) for cu_hengqw240724_1. The Anisotropic displacement factor exponent takes the form: $-2\pi^2[h^2a^{*2}U_{11}+2hka^*b^*U_{12}+\ldots]$.

Atom	U_{11}	U_{22}	U_{33}	U_{23}	U_{13}	U_{12}
O1	42.6(12)	30.5(10)	78.8(15)	-0.6(10)	19.7(11)	14.2(10)
O2	28.5(10)	55.7(13)	50.3(12)	3.1(10)	8.4(9)	19.4(10)
O3	52.1(12)	49.2(12)	39.4(11)	-2.4(9)	-8.0(9)	27.2(10)
N1	35.2(12)	35.2(12)	46.3(13)	-6.4(10)	-1.0(10)	21.8(11)
N2	25.2(11)	29.5(11)	36.5(12)	-0.4(9)	4.0(9)	12.8(9)
N3	39.3(13)	38.4(13)	39.6(12)	-8.0(10)	-2.4(10)	22.4(11)
C2	54(2)	69(2)	64(2)	-23.3(17)	-10.8(17)	45.3(19)
C1	43.9(17)	43.6(17)	63.4(19)	-17.1(14)	-11.2(14)	28.4(14)
C6	33.7(15)	30.3(14)	42.4(15)	-1.1(11)	4.7(12)	15.7(12)
C7	22.7(13)	30.2(13)	35.2(14)	2.7(11)	3.9(10)	12.7(11)
C8	25.8(12)	27.4(13)	34.0(14)	2.2(10)	1.8(10)	14.7(11)
C9	25.8(13)	26.9(13)	32.3(13)	-0.7(10)	0.2(10)	12.4(10)
C12	22.2(12)	25.8(12)	40.0(15)	4.1(11)	2.4(10)	10.6(10)
C13	33.0(14)	36.7(15)	39.7(15)	7.1(12)	3.6(12)	16.3(12)
C14	34.9(15)	47.0(17)	47.0(17)	17.7(13)	1.7(12)	18.2(14)
C17	33.8(16)	38.3(16)	72(2)	17.1(15)	6.2(15)	21.3(13)
C5	27.8(14)	44.8(16)	34.5(14)	-1.0(12)	0.8(11)	19.2(13)
C4	29.9(14)	48.7(16)	38.3(15)	-5.9(12)	-0.5(11)	24.0(13)
C3	44.3(17)	61(2)	45.7(17)	-14.8(15)	1.4(13)	30.8(16)
C10	27.8(13)	29.5(14)	43.4(16)	1.6(11)	4.5(11)	14.2(11)
C15	37.5(15)	34.5(15)	62.0(19)	5.1(13)	6.2(13)	21.8(13)
C16	25.6(13)	27.1(13)	47.5(16)	4.0(11)	5.4(11)	12.1(11)
C11	29.2(13)	30.8(14)	39.9(16)	-1.1(11)	-1.5(11)	14.0(11)

Table S9 Anisotropic Displacement Parameters (Å^2×10^3) for cu_hengqw240724_1. The Anisotropic displacement factor exponent takes the form: -2π^2[h^{2}a*^{2}U$_{11}$+2hka*b*U$_{12}$+…].

Atom	U$_{11}$	U$_{22}$	U$_{33}$	U$_{23}$	U$_{13}$	U$_{12}$
C24	28.5(13)	32.4(14)	37.7(14)	2.5(11)	4.0(11)	17.1(11)
C26	30.5(14)	34.4(14)	47.7(16)	7.2(12)	8.2(12)	13.7(12)
C25	33.0(15)	44.6(17)	55.6(19)	-6.8(13)	11.5(13)	17.3(13)
C18	27.2(14)	45.8(14)	60.3(18)	-12.9(13)	-1.3(12)	19.8(12)
C23	35.7(15)	49.9(15)	65(2)	-20.1(15)	11.7(13)	6.6(13)
C22	39.4(18)	85(3)	88(3)	-45.7(19)	2.8(16)	12.9(18)
C21	46(2)	126(3)	96(3)	-68(2)	-25(2)	49(2)
C20	82(3)	117(3)	109(3)	-52(3)	-54(2)	73(2)
C19	66(2)	70(2)	82(3)	-24.3(18)	-35.2(19)	45.2(19)

Table S10 Bond Lengths for cu_hengqw240724_1.

Atom	Atom	Length/Å	Atom	Atom	Length/Å
O1	C6	1.228(3)	C9	C11	1.540(4)
O2	C5	1.237(3)	C12	C13	1.382(4)
O3	C11	1.220(3)	C12	C16	1.399(4)
N1	C1	1.473(4)	C13	C14	1.399(4)
N1	C6	1.337(3)	C14	C17	1.383(5)
N1	C4	1.474(4)	C17	C15	1.378(5)
N2	C7	1.464(3)	C5	C4	1.492(4)
N2	C5	1.345(3)	C4	C3	1.520(4)
N2	C10	1.466(3)	C10	C18	1.516(4)
N3	C16	1.404(4)	C15	C16	1.383(4)
N3	C11	1.358(4)	C24	C26	1.498(4)
C2	C1	1.535(5)	C24	C25	1.505(4)
C2	C3	1.531(5)	C26	C25	1.503(4)
C6	C7	1.514(4)	C18	C23	1.378(4)
C7	C8	1.524(3)	C18	C19	1.372(5)
C8	C9	1.561(3)	C23	C22	1.385(6)
C8	C24	1.511(3)	C22	C21	1.370(7)
C9	C12	1.511(4)	C21	C20	1.368(7)
C9	C10	1.577(3)	C20	C19	1.378(5)

Table S11 Bond Angles for cu_hengqw240724_1.

Atom	Atom	Atom	Angle/°	Atom	Atom	Atom	Angle/°
C1	N1	C4	109.8(2)	O2	C5	N2	122.9(3)
C6	N1	C1	120.1(2)	O2	C5	C4	120.7(2)

Table S11 Bond Angles for cu_hengqw240724_1.

Atom	Atom	Atom	Angle/°	Atom	Atom	Atom	Angle/°
C6	N1	C4	126.3(2)	N2	C5	C4	115.7(2)
C7	N2	C10	112.86(19)	N1	C4	C5	115.4(2)
C5	N2	C7	123.7(2)	N1	C4	C3	101.9(2)
C5	N2	C10	121.8(2)	C5	C4	C3	119.1(2)
C11	N3	C16	111.6(2)	C4	C3	C2	101.0(3)
C3	C2	C1	105.1(2)	N2	C10	C9	103.06(19)
N1	C1	C2	103.8(2)	N2	C10	C18	111.3(2)
O1	C6	N1	122.6(2)	C18	C10	C9	116.5(2)
O1	C6	C7	120.3(2)	C17	C15	C16	117.7(3)
N1	C6	C7	116.9(2)	C12	C16	N3	109.5(2)
N2	C7	C6	113.0(2)	C15	C16	N3	128.5(3)
N2	C7	C8	102.83(19)	C15	C16	C12	122.0(3)
C6	C7	C8	117.8(2)	O3	C11	N3	125.4(3)
C7	C8	C9	102.94(19)	O3	C11	C9	126.3(2)
C24	C8	C7	115.2(2)	N3	C11	C9	108.3(2)
C24	C8	C9	113.8(2)	C26	C24	C8	120.6(2)
C8	C9	C10	104.92(19)	C26	C24	C25	60.08(19)
C12	C9	C8	116.1(2)	C25	C24	C8	119.6(2)
C12	C9	C10	111.7(2)	C24	C26	C25	60.18(18)
C12	C9	C11	102.0(2)	C26	C25	C24	59.74(18)
C11	C9	C8	108.3(2)	C23	C18	C10	119.1(3)
C11	C9	C10	114.0(2)	C19	C18	C10	121.8(3)
C13	C12	C9	131.9(2)	C19	C18	C23	119.0(3)
C13	C12	C16	119.5(2)	C18	C23	C22	120.6(4)
C16	C12	C9	108.6(2)	C21	C22	C23	119.3(4)
C12	C13	C14	118.7(3)	C20	C21	C22	120.5(4)
C17	C14	C13	120.5(3)	C21	C20	C19	119.8(5)
C15	C17	C14	121.5(3)	C18	C19	C20	120.6(4)

Table S12 Torsion Angles for cu_hengqw240724_1.

A	B	C	D	Angle/°	A	B	C	D	Angle/°
O1	C6	C7	N2	168.5(3)	C12	C9	C10	C18	124.2(3)
O1	C6	C7	C8	48.8(4)	C12	C9	C11	O3	174.5(3)
O2	C5	C4	N1	-178.4(3)	C12	C9	C11	N3	-3.3(3)
O2	C5	C4	C3	-56.6(4)	C12	C13	C14	C17	1.4(4)
N1	C6	C7	N2	-16.5(3)	C13	C12	C16	N3	-177.8(2)
N1	C6	C7	C8	-136.2(2)	C13	C12	C16	C15	0.7(4)
N1	C4	C3	C2	-42.0(3)	C13	C14	C17	C15	-0.6(4)
N2	C7	C8	C9	36.2(2)	C14	C17	C15	C16	-0.2(4)
N2	C7	C8	C24	160.7(2)	C17	C15	C16	N3	178.3(3)

Table S12 Torsion Angles for cu_hengqw240724_1.

A	B	C	D	Angle/˚	A	B	C	D	Angle/˚
N2	C5	C4	N1	10.9(3)	C17	C15	C16	C12	0.1(4)
N2	C5	C4	C3	132.6(3)	C5	N2	C7	C6	36.2(3)
N2	C10	C18	C23	145.7(3)	C5	N2	C7	C8	164.2(2)
N2	C10	C18	C19	-33.9(4)	C5	N2	C10	C9	176.4(2)
C1	N1	C6	O1	16.6(4)	C5	N2	C10	C18	-58.0(3)
C1	N1	C6	C7	-158.3(3)	C5	C4	C3	C2	-170.2(2)
C1	N1	C4	C5	164.1(2)	C4	N1	C1	C2	-10.3(3)
C1	N1	C4	C3	33.5(3)	C4	N1	C6	O1	172.6(3)
C1	C2	C3	C4	36.6(3)	C4	N1	C6	C7	-2.2(4)
C6	N1	C1	C2	149.3(3)	C3	C2	C1	N1	-16.9(3)
C6	N1	C4	C5	6.0(4)	C10	N2	C7	C6	-158.3(2)
C6	N1	C4	C3	-124.6(3)	C10	N2	C7	C8	-30.3(3)
C6	C7	C8	C9	161.2(2)	C10	N2	C5	O2	-7.8(4)
C6	C7	C8	C24	-74.3(3)	C10	N2	C5	C4	162.7(2)
C7	N2	C5	O2	156.4(2)	C10	C9	C12	C13	57.3(4)
C7	N2	C5	C4	-33.1(4)	C10	C9	C12	C16	-120.4(2)
C7	N2	C10	C9	10.6(3)	C10	C9	C11	O3	-64.8(3)
C7	N2	C10	C18	136.2(2)	C10	C9	C11	N3	117.3(2)
C7	C8	C9	C12	93.3(2)	C10	C18	C23	C22	-177.9(3)
C7	C8	C9	C10	-30.6(2)	C10	C18	C19	C20	178.8(4)
C7	C8	C9	C11	-152.7(2)	C16	N3	C11	O3	-174.2(3)
C7	C8	C24	C26	100.5(3)	C16	N3	C11	C9	3.7(3)
C7	C8	C24	C25	171.2(2)	C16	C12	C13	C14	-1.4(4)
C8	C9	C12	C13	-63.0(3)	C11	N3	C16	C12	-2.5(3)
C8	C9	C12	C16	119.3(2)	C11	N3	C16	C15	179.1(3)
C8	C9	C10	N2	13.0(2)	C11	C9	C12	C13	179.5(3)
C8	C9	C10	C18	-109.2(3)	C11	C9	C12	C16	1.8(3)
C8	C9	C11	O3	51.5(3)	C11	C9	C10	N2	131.3(2)
C8	C9	C11	N3	-126.3(2)	C11	C9	C10	C18	9.2(3)
C8	C24	C26	C25	108.7(3)	C24	C8	C9	C12	-32.0(3)
C8	C24	C25	C26	-110.5(3)	C24	C8	C9	C10	-155.9(2)
C9	C8	C24	C26	-141.0(2)	C24	C8	C9	C11	81.9(3)
C9	C8	C24	C25	-70.3(3)	C18	C23	C22	C21	-1.2(5)
C9	C12	C13	C14	-178.9(3)	C23	C18	C19	C20	-0.8(6)
C9	C12	C16	N3	0.2(3)	C23	C22	C21	C20	-0.3(6)
C9	C12	C16	C15	178.7(2)	C22	C21	C20	C19	1.3(7)
C9	C10	C18	C23	-96.6(3)	C21	C20	C19	C18	-0.7(7)
C9	C10	C18	C19	83.8(4)	C19	C18	C23	C22	1.7(5)
C12	C9	C10	N2	-113.7(2)					

Table S13 Hydrogen Atom Coordinates (Å×10⁴) and Isotropic Displacement Parameters (Å²×10³) for cu_hengqw240724_1.

Atom	*x*	*y*	*z*	U(eq)
H3	3051.98	5001.98	6426.69	45
H2A	4465.25	353.91	4067.82	67
H2B	3759.5	101.95	3448.42	67
H1A	2625.54	-67.61	4143.63	57
H1B	3235.03	-130.81	4746.09	57
H7	2661.8	2358.75	4393.38	36
H8	2780.95	2380.16	5837.97	34
H13	2599.62	3692.88	4008.51	44
H14	1981.98	4599	3651.27	53
H17	1851.02	5578.96	4404.29	56
H4	4790.16	1812.25	4693.88	44
H3A	5052.85	1766.17	3551.7	58
H3B	4051.6	1592.55	3354.64	58
H10	4268.71	4255.79	4650.58	40
H15	2296.18	5668.19	5534.11	52
H24	1321.4	2263.15	4990.49	38
H26A	1265.98	1168.41	6160.12	46
H26B	367.01	1000.91	5700.71	46
H25A	1575.22	2691.71	6444.5	54
H25B	675.98	2524.15	5984.96	54
H23	5410.3	5547.89	5234.56	68
H22	6722.71	6193.45	5905.74	95
H21	7022.15	5261.62	6594.24	105
H20	6021.15	3703.14	6624.04	111
H19	4735.83	3058.24	5933.28	81
